# SmI_2_-mediated dimerization of indolylbutenones and synthesis of the myxobacterial natural product indiacen B

**DOI:** 10.3762/bjoc.11.184

**Published:** 2015-09-21

**Authors:** Nils Marsch, Peter G Jones, Thomas Lindel

**Affiliations:** 1 Institute of Organic Chemistry, TU Braunschweig, Hagenring 30, 38106 Braunschweig, Germany, Fax: (+49) 531-391-7744,; 2 Institute of Inorganic and Analytical Chemistry, TU Braunschweig, Hagenring 30, 38106 Braunschweig, Germany

**Keywords:** biological evaluation, heterocycles, indoles, natural products, total synthesis

## Abstract

The synthesis and reactivity of indole derivatives substituted in the benzene section was studied. Starting materials 4- and 6-iodoindole were conveniently prepared via the Batcho–Leimgruber route and purified by sublimation. Novel vicinally indolyl-substituted cyclopentanols with unexpected *cis*-configuration were formed by SmI_2_-mediated reductive dimerization of a 4-(indol-6-yl)butenone, obtained by Heck reaction. The two indolyl units appear to chelate Sm(II)/(III) leading to a *gauche*-type arrangement at the newly formed bond between the two β-carbons. Through a sequence of Sonogashira cross coupling and Meyer–Schuster rearrangement 6-prenoylindole was synthesized and reductively dimerized to a cyclopentane in a [3 + 2] cycloaddition by treatment with SmI_2_ in THF. From 4-iodoindole, the natural product indiacen B from the myxobacterium *Sandaracinus amylolyticus* was synthesized for the first time, confirming its antimicrobial activity. The *E*-configuration of the chloroalkene moiety of indiacen B was confirmed by X-ray analysis.

## Introduction

**Figure 1:**
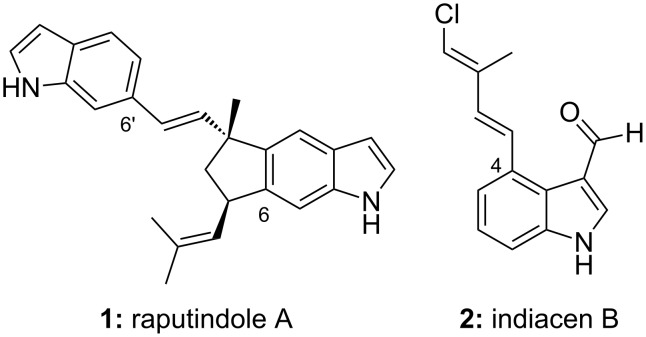
Prenylated indole alkaloids raputindole A from the rutaceous tree *Raputia simulans* and indiacen B from the myxobacterium *Sandaracinus amylolyticus*.

Indole alkaloids prenylated at the benzene ring are found in tropical plants [1–3] and also in fungi [4] and myxobacteria [5–6]. One example is raputindole A (**1**), isolated in 2010 from the Rutaceous tree *Raputia simulans* Kallunki [1], which exhibits a unique tetrahydrocyclopenta[*f*]indole partial structure (Figure 1) probably formed by dimerization of (*E*)-6-(3-methylbuta-1,3-dien-1-yl)indole [7]. All enamine positions of the indole units are unsubstituted. As part of our program on the total synthesis of prenylated indole alkaloids [8–11], we considered it interesting to access indoles substituted only at the benzene ring and to conduct initial studies on their reductive dimerization. We also describe the synthesis of the antimicrobial natural product indiacen B (**2**) from the myxobacterium *Sandaracinus amylolyticus* [5], bearing a unique isoprene moiety chlorinated at the methylene group.

## Results and Discussion

### SmI_2_-induced reductive dimerizations

**Scheme 1:**
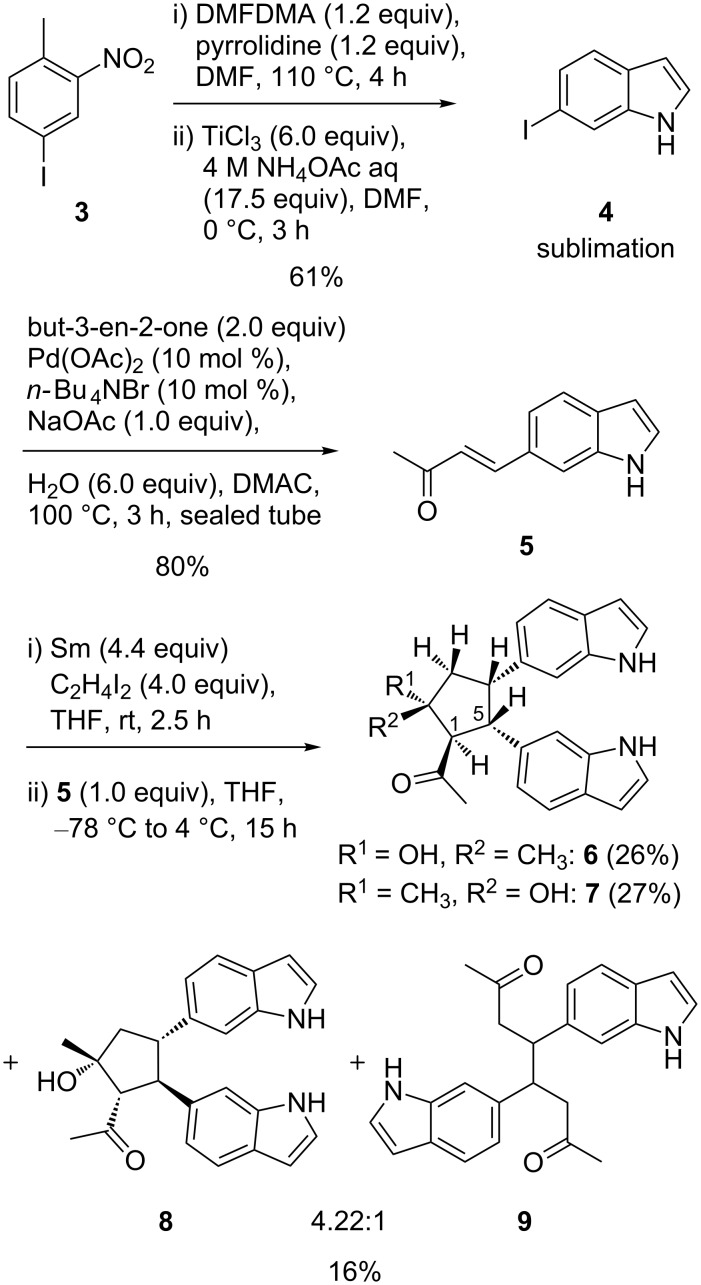
Synthesis and SmI_2_-mediated reductive dimerization of natural product **5**.

6-Iodoindole (**4**) was prepared from 1-iodo-4-methyl-3-nitrobenzene (**3**, accessible from the corresponding nitroaniline via Sandmeyer reaction) via the Batcho–Leimgruber protocol employing dimethylformamide dimethyl acetal (DMFDMA) and TiCl_3_ in one step. It should be noted that, after initial column filtration of the crude material, 6-iodoindole was purified conveniently by sublimation in high vacuum affording a colorless product. The natural product (*E*)-4-(1*H*-indol-6-yl)but-3-en-2-one (**5**) isolated from *Monodora angolensis* [12] was obtained from 6-iodoindole (**4**) in one step via Heck reaction with methyl vinyl ketone. Protection of the indole nitrogen was not necessary and **5** was obtained as a bright yellow solid in excellent yield (Scheme 1). The route appears more efficient than earlier procedures via 6-formylindoles [13–15]. There have been only few Pd-catalyzed cross-coupling reactions of unprotected 6- or 4-iodinated indoles reported [16–20].

Having natural product **5** in hand, we wondered whether and which intra or intermolecular C–C coupling would occur under reductive conditions and whether or not such a reaction could run without participation of the indole enamine moiety. After treatment of the indolylbutenone **5** with samarium diiodide in THF (formed from Sm and diiodoethane), the dimeric bisindolylcyclopentanes **6** (26%), **7** (27%) and a mixture of **8** and the open-chain dimer **9** (16%, 4:1) were isolated by semipreparative HPLC. The structures were elucidated by 2D NMR spectroscopy and assignment of the relative configuration of compounds **6**–**8** was possible on the basis of NOESY spectra. The relative configuration of flexible compound **9** could not be determined. Since NOESY cross peaks may result from different conformations in equilibrium, we first identified positions which were likely to be too distant from each other to show an nOe. In any cyclopentane conformation this would be the case for hydrogen atoms located in 1,3-*trans*-position. Thus, the presence of a NOESY cross peak would exclude 1,3-*trans*-orientation of the respective hydrogens. In diastereomers **6**, **7**, and **8** there were five 1,3-pairs to be analyzed. In compound **6**, methine hydrogens 5-H (4.10 ppm) and 1-H (3.88 ppm) show NOESY correlations to the diastereotopic methylene hydrogens 3-H^2^ (2.35 ppm) and 3-H^1^ (2.67 ppm), respectively. Thus, 5-H and 1-H have to be 1,2-*trans*-located. Since methylene hydrogen 3-H^1^ shows a NOESY correlation only with 1-H, the distance of 3-H^1^ to the vicinal hydrogen 4-H (3.73 ppm) must be greater than to 1,3-*cis*-located 1-H, implying that 4-H is 1,2-*trans*-located to 3-H^1^. The methyl substituent (1.37 ppm) shows NOESY correlations only to 5-H, 4-H, and 3-H^2^, putting it on the same side. Differing from compound **6**, there is no NOESY correlation in compound **7** between 5-H (4.39 ppm) and any of the methylene hydrogens 3-H^1^ (2.50 ppm) or 3-H^2^ (2.31 ppm). Thus, positioning of 5-H 1,2-*cis* to 4-H has to rely on the more intense NOESY correlation of 5-H to 4-H than to 3-H. This is corroborated by the NOESY correlations of the methyl group (1.69 ppm) to 1-H and to 3-H^1^, but not to 5-H. Hydrogen 1-H (3.44 ppm) shows a NOESY correlation to methylene hydrogen 3-H^1^. Thus, 1-H and 3-H^1^ have to be 1,3-*cis*-located. The intensity of the second NOESY correlation of 3-H^1^ with hydrogen 4-H is weaker, which means that 4-H is 1,2-*trans*-located to 3-H^1^. As in compound **6**, 5-H (3.98 ppm) and 1-H (3.28 ppm) of compound **8** show NOESY correlations to only one of the diastereotopic hydrogens 3-H^2^ (2.21 ppm) and 3-H^1^ (2.46 ppm), respectively. Thus, 5-H and 1-H have to be 1,2-*trans*-located. Differing from compound **6**, the NOESY correlation between 3-H^1^ and 4-H (3.43 ppm) is stronger than between 3-H^1^ and 1-H, which places 4-H in the 1,2-*cis*-position to 3-H^1^. Further NOESY correlations place the methyl group (1.59 ppm) on the side of 1-H and 3-H^2^.

The major bisindolylcyclopentanes **6** and **7** carry the indolyl substituents in a *cis* arrangement and differ regarding the configuration of the tertiary alcohol moiety. Minor bisindolylcyclopentane **8** exhibits a *trans* arrangement of the indolyl substituents. For the great majority of intermolecular reductive dimerizations of α,β-unsaturated β-arylketones induced by SmI_2_, exclusive *trans*-arrangement of aryl substituents at the cyclopentane ring had been observed [21–24]. Aryl substituents generally prefer the antiperiplanar arrangement, as observed by Zhou and Zhang, who reductively dimerized chalcone derivatives to *trans*-diarylated cyclopentanols [23]. *Cis* isomers had been obtained in the case of intramolecular reactions where the β-positions of the enone systems were covalently tethered. Shinohara et al. have also reported a reductive dimerization of methyl cinnamate resulting in a *cis-*orientation of the phenyl rings [25]. SmI_2_-induced reductive dimerization of 1,1'-dicinnamoylferrocenes had also afforded *cis*-phenyl-disubstituted cyclopentanols, presumably as a consequence of parallel orientation of the enone systems by fixation of the carbonyl oxygens by Sm(III) and tethering by the ferrocene unit [26].

**Scheme 2:**
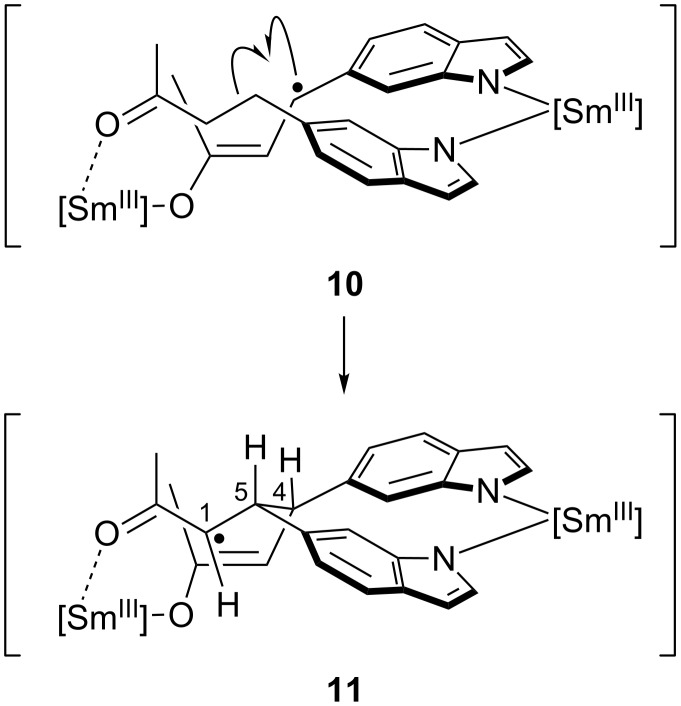
Model visualizing the stereochemical course of the cyclopentanol formation leading to product **6**. Position numbering corresponds to products **6** and **7**.

A possible reason for our findings could be the capability of a deprotonated indole ring to engage into complexation with Sm(III) formed by reduction of the enone moiety (Scheme 2), leading to a *gauche*-type orientation of the indole moieties, as shown in structures **10** and **11**. It is known that anionic indolide can form complexes with Sm(II) and Sm(III) [27–28]. Thus, the indole NH group (p*K*_a_ about 21 in DMSO) should be able to serve as a stoichiometric proton source that, after β,β'-coupling, would convert one of the Sm(III) enolates to the ketone. Attack of the remaining Sm(III) enolate would lead to Dieckmann-type ring closure, followed by protonation of the Sm(III) alcoholate by the second indole equivalent.

We did not observe addition of the intermediate oxycarbonyl radicals to the 2- or 3-position of the unsubstituted indole, which could have occurred as intermolecular reaction. This is in accordance with results by Reissig et al. who observed an intermolecular reaction with ketones only if the 3-position was substituted with an electron-withdrawing group [29]. There are also several examples of SmI_2_-induced cyclization of indolylketones [30]. Further applications of samarium reagents in organic synthesis can be found in reviews by Nicolaou and Procter [31–32].

**Scheme 3:**
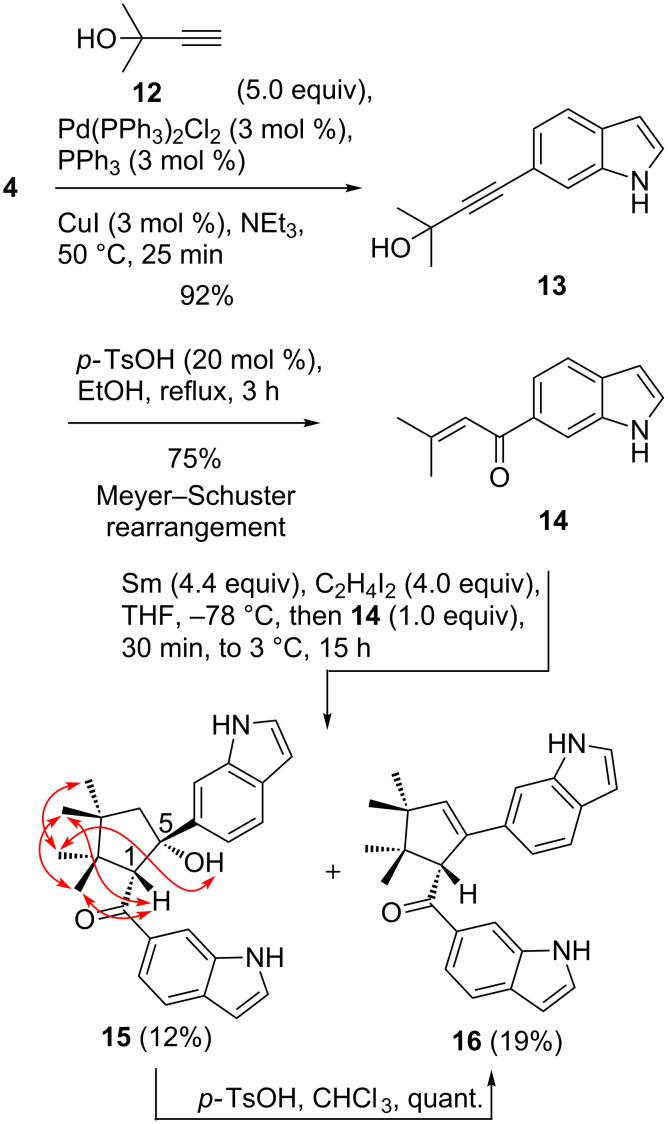
Meyer–Schuster rearrangement of **13** and SmI_2_-mediated reductive [3 + 2] cycloaddition, followed by elimination to cyclopentene **16**. Selected NOESY correlations for compound **15** are given.

The pattern of geometrical [3 + 2] cycloaddition was also obtained with inverted enone moieties. 6-Prenoylindole (**14**) was synthesized in two steps from 6-iodoindole (**4**) by Sonogashira coupling with propargylic alcohol **12** and subsequent Meyer–Schuster rearrangement [33]. Treatment of indole **14** with SmI_2_ afforded the cyclic bisindole **15** and, after silica column chromatography, its elimination product cyclopentene **16**. On the TLC only the tertiary alcohol **15** and full consumption of the starting material were observed. Product **15** was converted quantitatively to cyclopentene **16** by treatment with *p-*TsOH (20 mol %) in chloroform (Scheme 3). As a consequence of smaller ring strain, the double bond is formed in β,γ-position to the carbonyl group.

The relative configuration of tertiary alcohol **15** was determined on the basis of NOESY correlations, which revealed that 1-H (4.58 ppm) was located on the same side of the cyclopentane ring as the methyl groups 2-CH_3_ and 3-CH_3_ with chemical shifts at 0.88 and 1.34 ppm, respectively. The other diastereotopic methyl group (1.25 ppm) at C2 correlates with the hydroxy proton (5-OH, 6.55 ppm), which does not correlate with the methyl group at 0.88 ppm. We conclude that the hydroxy group and 1-H are most probably situated on opposite sides of the cyclopentane ring.

**Scheme 4:**
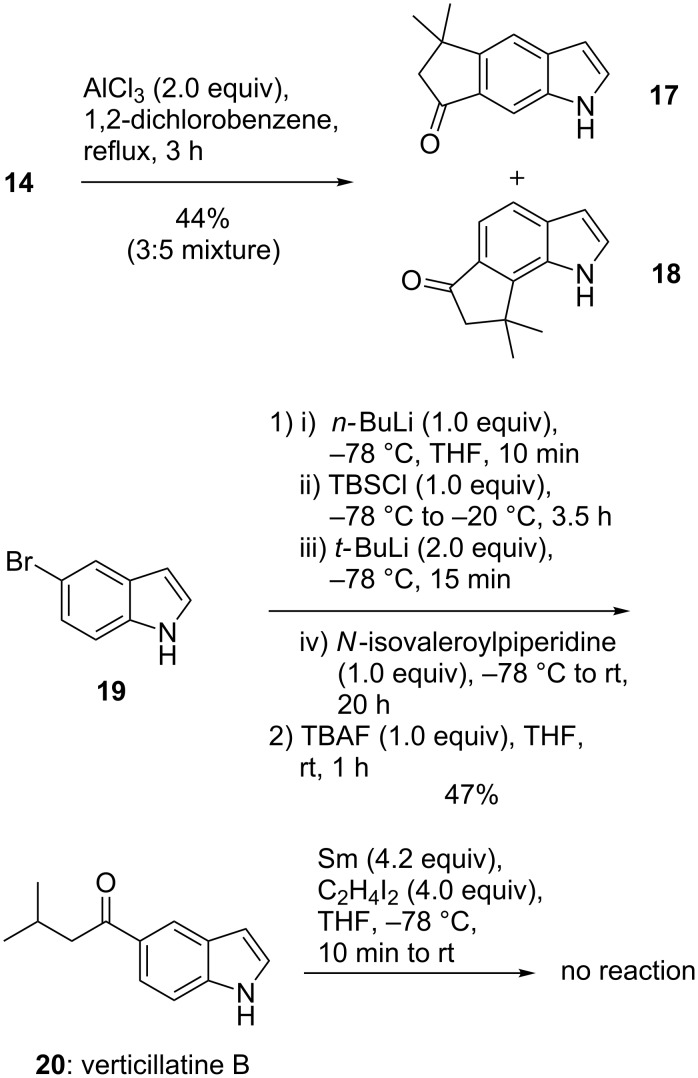
Nazarov-type cyclization of **14** to cyclopentanones **17** and **18**; synthesis of verticillatine B (**20**).

The only case where we observed the indole nucleus participating in a cyclization was a Nazarov-type reaction of indole **14**. We treated 6-prenoylindole (**14**) with AlCl_3_ in dichlorobenzene at 150 °C to obtain a 3:5 mixture of the tricyclic cyclopentanones **17** and **18** (Scheme 4) [34]. Almost no regioselectivity between the 5- and 7-positions was observed. Our ongoing studies investigate the influence of bulky N-protecting groups.

In the absence of the α,β-double bond, SmI_2_ in THF did not effect any reaction, as we learned when trying to dimerize the natural product verticillatine B (**20**) from the Brazilian plants *Borreria verticillata* and *Raputia simulans* [2–3]. We had speculated on the participation of the benzenoid double bond in dimerization reactions which would have functionalized the indole 6-position. Verticillatine B (**20**) was synthesized in a short sequence from commercially available 5-bromoindole (**19**) via halogen–metal exchange (Scheme 4). The indole nitrogen was first protected with a TBS group, followed by bromine–lithium exchange with *tert*-butyllithium [35]. The aliphatic side chain was introduced via *N-*isovaleroylpiperidine [36]. Removal of the TBS group by treatment with TBAF gave the natural product **20** in 47% yield as a colorless solid.

### Synthesis and biological activity of indiacen B

**Scheme 5:**
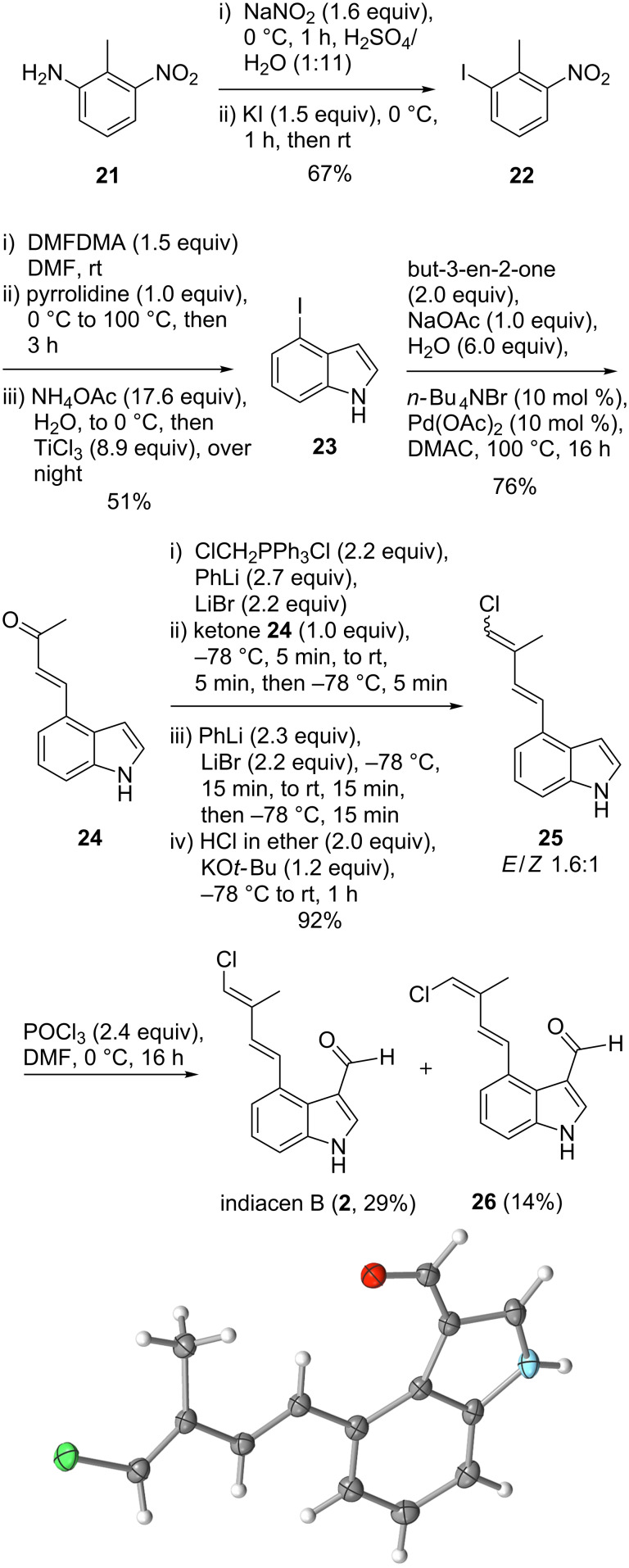
Synthesis and X-ray analysis of indiacen B (**2**, ORTEP drawing with ellipsoides at 50% probability).

The successful Heck reaction at unprotected 6-iodoindole prompted us to check the conversion of free 4-iodoindole to the natural product indiacen B (**2**), which had been isolated from the myxobacterium *Sandaracinus amylolyticus* (Scheme 5) [5]. 4-Iodoindole (**23**) was conveniently synthesized from 2-methyl-3-nitroaniline (**21**). Enone **24** was obtained without difficulties via Heck reaction of **23** with but-3-en-2-one in dimethylacetamide at 100 °C (76%). Enone **24** is a yellow solid, which had been obtained as an intermediate in the total syntheses of clavicipitic acid and 6,7-secoagroclavine in a four-step sequence starting from indole, but never in one step [37].

For achieving *E*-selectivity we attempted to adapt Schlosser's procedure for the olefination of aldehydes to the chloromethylenation of ketone **24** [38]. Selectivity towards the *E*-isomer was rather poor with an *E*/*Z*-ratio of 1.6:1 (Scheme 5). Separation of indiacen B (**2**) and its *Z*-isomer **26** was possible by semipreparative normal phase HPLC. While the natural product **2** was stable under normal conditions as a colorless solid, the *Z*-isomer **26** proved to be rather unstable. To our delight, indiacen B (**2**) crystallized from a DCM/*n*-hexane mixture. Thus, we were able to confirm the *E*-configuration of the chloroalkene moiety in the natural product via X-ray analysis.

Indiacen B (**2**) was tested against the Gram-positive bacteria *Nocardioides simplex* (1.7 µg/mL), *Mycobacterium diernhoferi* (no inhibition), and *Mycobacterium* sp. (8.3 µg/mL), and against the Gram-negative bacteria *Escherichia coli* TOL C (8.3 µg/mL), *Chromobacterium violaceum* (67 µg/mL), and *Pseudomonas stutzeri* (no inhibition), and also against the fungus *Mucor hiemalis* (8.3 µg/mL). The antimicrobial activity of indiacen B (**2**) against *M. diernhoferi*, which was described by Müller et al. [5] in course of the isolation from the myxobacterium *Sandaracinus amylolyticus*, was confirmed. Against *N. simplex* and *M. hiemalis* the activity was twofold and against *E. coli* TOL C fourfold higher than observed for the isolated sample. Against *C. violaceum* a slightly weaker activity was observed. Overall, the antimicrobial activity of indiacen B (**2**) can be regarded as confirmed.

## Conclusion

Indole derivatives prenylated at the benzene section could become important for the biomimetic synthesis of dimeric natural products such as the raputindoles from the plant *Raputia simulans*. Characteristically, the three enamine positions of the indole moieties of the raputindoles are unsubstituted. As a step towards the exploration of their chemistry, we achieved the synthesis of potential monomers with oxygenated side chains, some of which occur as natural products themselves (**5**, **20**). For the first time, the SmI_2_-mediated reductive dimerization of (indol-6-yl)butenone derivatives was studied, affording novel vicinally indolyl-substituted cyclopentane derivatives. The aryl groups in products **6** and **7** are located in the *cis*-position, thus differing from their phenyl analogs. The two indolyl units appear to chelate Sm(II)/(III) leading to a *gauche*-type arrangement at the newly formed bond between the two β-carbons. When the enone unit was connected to the indole 6-position via the carbonyl moiety, a deconjugated cyclopentene (**16**) became accessible. In the absence of the α,β-double bond as in the natural product verticillatine B (**20**), SmI_2_ in THF was not able to induce a dimerization and no pinacol products were found. Anellation of a cyclopentane ring at the benzene section of 1,2,3-unsubstituted indole was possible by Nazarov-type cyclization, albeit with no regioselectivity regarding the 5- and 7-positions. The *E*-configuration of the chloroalkene moiety in the myxobacterial natural product indiacen B (**2**), which was synthesized for the first time, was confirmed by X-ray analysis. The antimicrobial activity of synthetic indiacen B (**2**) was in the same range as that originally determined for the isolated natural product.

## Supporting Information

File 1 Experimental procedures, bioactivity tables, X-ray figures and tables, HPLC chromatograms and NMR spectra for all compounds, NOESY analyses.

## References

[1] Vougogiannopoulou K, Fokialakis N, Aligiannis N, Cantrell C, Skaltsounis A-L (2010). Org Lett.

[2] Vougogiannopoulou K, Fokialakis N, Aligiannis N, Cantrell C, Skaltsounis A-L (2011). Planta Med.

[3] Moreira V F, Oliveira R R, Mathias L, Braz-Filho R, Curcino Vieira I J (2010). Helv Chim Acta.

[4] Li S-M (2010). Nat Prod Rep.

[5] Steinmetz H, Mohr K I, Zander W, Jansen R, Gerth K, Müller R (2012). J Nat Prod.

[6] Jansen R, Mohr K I, Bemecker S, Stadler M, Müller R (2014). J Nat Prod.

[7] Nwaji M N, Onyiriuka S O, Taylor D A H (1972). J Chem Soc, Chem Commun.

[8] Lindel T, Bräuchle L, Golz G, Böhrer P (2007). Org Lett.

[9] Adla S K, Golz G, Jones P G, Lindel T (2010). Synthesis.

[10] Adla S K, Sasse F, Kelter G, Fiebig H-H, Lindel T (2013). Org Biomol Chem.

[11] Lindel T, Marsch N, Adla S K (2012). Top Curr Chem.

[12] Nkunya M H H, Makangara J J, Jonker S A (2004). Nat Prod Res.

[13] Ishii H, Murakami Y, Furuse T, Takeda H, Ikeda N (1973). Tetrahedron Lett.

[14] Ishii H, Murakami Y (1975). Tetrahedron.

[15] Somei M (1986). Chem Pharm Bull.

[16] Benhida R, Lecubin F, Fourrey J-L, Castellanos L R, Quintero L (1999). Tetrahedron Lett.

[17] Falck J R, Patel P K, Bandyopadhyay A (2007). J Am Chem Soc.

[18] Goli M, He A, Falck J R (2011). Org Lett.

[19] Murase M, Watanabe K, Kurihara T, Tobinaga S (1998). Chem Pharm Bull.

[20] Sagadevan A, Hwang K C (2012). Adv Synth Catal.

[21] Kanemesa S, Yamamoto H, Kobayashi S (1996). Tetrahedron Lett.

[22] Jong S-J, Chen C-T, Fang J-M, Liu Y-H, Lee G-H, Wang Y (2001). C R Acad Sci, Ser IIc: Chim.

[23] Zhou L, Zhang Y (2000). Synth Commun.

[24] Cabrera A, Le Lagadec R, Sharma P, Arias J L, Toscano R A, Velasco L, Gaviño R, Alvarez C, Salmón V (1998). J Chem Soc, Perkin Trans 1.

[25] Shinohara I, Okue M, Yamada Y, Nagaoka H (2003). Tetrahedron Lett.

[26] Jong S-J, Fang J-M (2000). Org Lett.

[27] Evans W J, Brady J C, Ziller J W (2002). Inorg Chem.

[28] Zhu X, Wang S, Zhou S, Wei Y, Zhang L, Wang F, Feng Z, Guo L, Mu X (2012). Inorg Chem.

[29] Blot V, Reissig H-U (2006). Synlett.

[30] Beemelmanns C, Gross S, Reissig H-U (2013). Chem – Eur J.

[31] Nicolaou K C, Ellery S P, Chen J S (2009). Angew Chem, Int Ed.

[32] Szostak M, Procter D J (2012). Angew Chem, Int Ed.

[33] Jacubert M, Provot O, Peyrat J-F, Hamze A, Brion J-D, Alami M (2010). Tetrahedron.

[34] Kern J C, Terefenko E, Trybulski E, Berrodin T J, Cohen J, Winneker R C, Yudt M R, Zhang Z, Zhu Y, Zhang P (2009). Bioorg Med Chem Lett.

[35] Moyer M P, Shiurba J F, Rapoport H (1986). J Org Chem.

[36] Nakatsuji H, Morimoto M, Misaki T, Tanabe Y (2007). Tetrahedron.

[37] Semmelhack M F, Knochel P, Singleton T (1993). Tetrahedron Lett.

[38] Wang Q, Deredas D, Huyhn C, Schlosser M (2003). Chem – Eur J.

