# Supplementary material for: SmI2-mediated dimerization of indolylbutenones and synthesis of the myxobacterial natural product indiacen B
Source: Beilstein J Org Chem. 2015 Sep 21;11:1700–6. doi: 10.3762/bjoc.11.184 (PMC4660955; doi:10.3762/bjoc.11.184)

Supporting Information

for

SmI_2-mediated dimerization of indolylbutenones and synthesis of the myxobacterial natural product indiacen B

Nils Marsch[1], Peter G. Jones[2] and Thomas Lindel*[,1§]

Address: [1]Institute of Organic Chemistry, TU Braunschweig, Hagenring 30, 38106 Braunschweig, Germany, Fax: (+49) 531-391-7744, and [2]Institute of Inorganic and Analytical Chemistry, TU Braunschweig, Hagenring 30, 38106 Braunschweig, Germany

Email: Thomas Lindel - th.lindel@tu-braunschweig.de

[§]http://www.oc.tu-bs.de/lindel

*Corresponding author

Experimental procedures, bioactivity tables, X-ray figures and tables, HPLC chromatograms and NMR spectra for all compounds, NOESY analyses

1. Experimental procedures

2. Crystallographic figures and tables for indiacen B (**2**)

3. Antimicrobial activity of indiacen B (**2**)

4. ^{1}H and ^{13}C NMR spectra and HPLC chromatograms; NOESY analyses

1. Experimental procedures

NMR spectra were recorded on a Bruker AV II-300 (300 MHz for ^{1}H, 75 MHz for ^{13}C), a Bruker DRX 400 or a Bruker AV III-400 (400 MHz for ^{1}H, 100 MHz for ^{13}C, 376 MHz for ^{19}F) and a Bruker AV-II 600 (600 MHz for ^{1}H, 150 MHz for ^{13}C) spectrometer at 299 K. Chemical shifts are given in ppm (δ scale) and referenced to TMS or the residual solvent peak. For all compounds, assignments of NMR chemical shifts are based on 2D NMR experiments, which always included COSY, HSQC, and HMBC spectra. ^{13}C NMR chemical shifts are normally given with one decimal, unless two clearly separated signals would give identical shift values. Mass spectra were obtained with a LTQ Orbitrap Velos, a Thermo Finnigan LTQ FT, a Thermo Finnigan MAT95 and a Finnigan MAT 95 XLT. GC–MS was performed on an Agilent Technologies 6890 gas chromatograph using a Phenomenex ZB5-MS 0.25 μm column (internal diameter: 0.25 mm, length: 30 m) and a JMST100GC (GCAccuTOF, JEOL, Japan) apparatus at 70 eV (EI). IR spectra were recorded on a Bruker Tensor 27 spectrometer with diamond ATR, wave numbers are given in cm^{-1}. UV–vis spectra were measured with a Varian Cary 100 Bio UV–vis spectrometer, wavelengths are given in nm. Melting points were measured with a Büchi 530 melting point apparatus and are uncorrected. Chemicals were purchased from commercial suppliers and used without further purification. Solvents were dried prior to use using standard methods unless noted. Flash column chromatography was performed on Merck silica gel 60 (40–63 μm). Thin-layer chromatography (TLC) was done on Merck silica gel 60 F_{254} and Merck silica gel 60 RP-18 F_{254S} aluminum sheets. HPLC separation was carried out with a Merck Hitachi intelligent pump. The following abbreviations have been used: DMAC (dimethylacetamide), PE (petrol ether), EA (ethyl acetate), TBME (*tert*-butyl methyl ether), HV (high vacuum).

6-Iodoindole (4): Starting material **3** (5.00 g, 19.0 mmol, 1.0 equiv) was dissolved in DMF (50 mL). DMFDMA (3.05 mL, 22.8 mmol, 1.2 equiv) was added, followed by pyrrolidine (1.90 mL, 23.1 mmol, 1.2 equiv). The mixture was heated to 110 °C for 4 h until complete consumption of starting material as monitored by TLC, and allowed to cool to rt. DMF (100 mL) and 4 M aqueous NH_4OAc buffer (83 mL, 17.5 equiv) were added and the solution was cooled with an ice bath. An aqueous solution of 20% $TiCl_3$ in 3% HCl (73.3 mL, 114.06 mmol, 6.0 equiv) was added via a dropping funnel. The reaction mixture was stirred for 3 h followed by extraction with TBME (3 × 100 mL). The organic phase was washed with 2 M NaOH (100 mL), H_2O (100 mL) and dried with $MgSO_4$. After evaporation of the solvent the raw material was prepurified by short column chromatography [PE/EA (15:1)] to be sublimated in HV at 110–115 °C to give the product as a slightly yellowish solid (2.83 g, 11.6 mmol, 61%). TLC [PE/EA (10:1)]: R_f = 0.25. Mp.: 103-105 °C. ^{1}H NMR (400 MHz, $CDCl_3$): δ = 8.10 (s_{br}, 1H, N*H*), 7.75 (dd, 1H, *J* = 2.0 Hz, *J* = 1.0 Hz, 4-*H*), 7.40-7.39 (m, 2H, 5-*H* and 7-*H*), 7.13 (dd, 1H, *J* = 3.2 Hz, *J* = 2.5 Hz, 2-*H*), 6.52 (ddd, 1H, *J* = 3.1 Hz, *J* = 2.0 Hz, *J* = 0.9 Hz, 3-*H*). ^{13}C NMR (100 MHz, $CDCl_3$): δ = 137.1 (1C, *C*-7a), 128.7 (1C, *C*-7), 127.2 (1C, *C*-3a), 124.5 (1C, *C*-2), 122.4 (1C, *C*-5), 120.0 (1C, *C*-4), 102.9 (1C, *C*-3), 85.8 (1C, *C*-6). IR (ATR): $\tilde{\nu}$ = 3888 (w), 3410 (s), 3094 (w), 3073 (w), 2925 (w), 1884 (w), 1726 (w), 1624 (w), 1597 (w), 1563 (w), 1493 (w), 1449 (m), 1392 (m), 1331 (m), 1309 (m), 1270 (w), 1232 (w), 1199 (w), 1090 (m), 1059 (w), 1043 (w), 998 (m), 944 (w), 878 (m), 858 (m), 802 (s), 759 (m), 727 (s), 651 (w), 607 (m), 586 (m), 565 (w). UV-Vis (MeOH): λ_{max} (lg ε) = 202 (4.32), 227 (4.62), 279 (3.86), 285 (3.86). MS (EI, 70 eV): *m/z* (%) = 243 $[M]^+$ (100), 116 $[M–I]^+$ (56), 89 (26), 63 (12).

(*E*)-4-(1*H*-Indol-6-yl)-but-3-en-2-one (5): In a sealed tube 6-iodoindole (**4**, 500 mg, 2.06 mmol, 1.0 equiv), but-3-en-2-one (334 µL, 4.12 mmol, 2.0 equiv), NaOAc (169 mg, 2.06 mmol, 1.0 equiv), $Pd(OAc)_2$ (47 mg, 0.21 mmol, 10 mol %), *n*-Bu_4NBr (68 mg, 0.21 mmol, 10 mol %) and H_2O (223 µL, 12.36 mmol, 6.0 equiv) were dissolved in *N*,*N*-dimethylacetamide (10 mL) and heated to 100 °C for 3 h. After cooling to rt the suspension was filtered with Celite 545. The Celite was washed with EA (100 mL) and the organic phase was washed with H_2O, $NaHCO_3$ and brine (10 mL each) and dried with $MgSO_4$. Purification via column chromatography [PE/EA (2:1)] yielded natural product **5** as a yellow solid (305 mg, 1.65 mmol, 80%). TLC [PE/EA (2:1)]: R_f = 0.28. Mp.: 129-130 °C. ^{1}H NMR ($CDCl_3$): δ = 8.77 (s_{br}, 1H, N*H*), 7.66 (d, 1H, *J* = 16.1 Hz, C*H*CHCO), 7.63 (d, 1H, *J* = 8.3 Hz, 4-*H*), 7.57 (s, 1H, 7-*H*), 7.34 (dd, 1H, *J* = 8.3 Hz, *J* = 1.5 Hz, 5-*H*), 7.30 (dd, 1H, *J* = 2.8 Hz, 2-*H*), 6.76 (d, 1H, *J* = 16.1 Hz, CHC*H*CO), 6.58-6.55 (m, 1H, 3-*H*), 2.38 (s, 3H, CH_3). ^{13}C NMR ($CDCl_3$): δ = 198.9 (1C, CO), 145.6 (1C, *C*HCHCO), 136.0 (1C, *C*-7a), 130.2 (1C, *C*-3a), 128.2 (1C, *C*-6), 126.9 (1C, *C*-2), 124.9 (1C, CH*C*HCO) 121.1 (1C, *C*-4), 119.7 (1C, *C*-5), 112.3 (1C, *C*-7), 103.0 (1C, *C*-3), 27.4 (1C, *C*H_3). IR (ATR): $\tilde{\nu}$ = 3325 (s), 3100 (w), 3031 (w), 3004 (w), 2921 (w), 2853 (w), 1671 (s), 1635 (m), 1583 (s), 1505 (w), 1481 (w), 1457 (m), 1413 (m), 1360 (m), 1338 (m), 1272 (s), 1213 (m), 1184 (m), 1155 (m), 1126 (m), 1102 (m), 1060 (m), 1008 (w), 970 (s), 893 (m), 803 (s), 763 (s), 731 (s), 671 (m), 625 (s), 577 (s), 541 (m). UV-Vis (MeOH): λ_{max} (lg ε) = 203 (4.21), 258 (4.11), 343 (4.28). HRESIMS: calcd. for $[C_{12}H_{11}NO+Na]^+$: 208.07329, found: 208.07325 (0.19 ppm).

Bisindoles 6, 7, 8, and 9: Samarium powder (~60 mesh, 358 mg, 2.38 mmol, 4.4 equiv) was dispersed in dry THF (10 mL) under argon. Diiodoethane (~800 mg) was dissolved in TBME (30 mL) and washed with sat. $Na_2S_2O_3$ (30 mL). The organic

phase was dried with $MgSO_4$, evaporated and dried under high vacuum to afford diiodoethane as a colorless solid. The purified diiodoethane (609 mg, 2.16 mmol, 4.0 equiv) was added to the samarium dispersion and the mixture was stirred for 2.5 h at room temperature. The color changed to dark blue. The mixture was cooled to –78 °C and ketone **5** (100 mg, 0.54 mmol, 1.0 equiv) in dry THF (2 mL) was slowly added via a syringe pump within 30 min. Afterwards the mixture was allowed to warm to 4 °C within 15 h and quenched with H_2O (10 mL). TBME (5 mL) and brine (30 mL) were added and the aqueous phase was further extracted with TBME (20 mL). The combined organic phases were washed with sat. $NaHCO_3$ and H_2O (20 mL each) and dried with $MgSO_4$, followed by evaporation of the solvent. The crude mixture was dissolved in iPrOH/hexane (20:80, 2 mL) and subjected to semi-preparative HPLC (flow rate: 4.6 mL/min, column: Merck KGaA, LiChroCART® 250-10, packed with LiChrospher® Si60, 5 µM, eluent: iPrOH/hexane (20:80), isocratic) to yield dimers **6** (26 mg, 0.14 mmol, 26%), **7** (27 mg, 0.15 mmol, 27%) and a mixture of **8** and **9** (16 mg, 0.09 mol, 16%) as yellow oils. Compound **6:** R_t = 32.64 min. ^{1}H NMR (400 MHz, acetone-d_6): δ = 9.91 (s_{br}, 1H, 1''-N*H*), 9.82 (s_{br}, 1H, 1'-N*H*), 7.25-7-22 (m, 1H, 7''-*H*), 7.11 (d, 2H, *J* = 8.2 Hz, 4' and 4''-*H*), 7.10-7.04 (m, 2H, 2' and 2''-*H*), 6.96 (s_{br}, 1H, 7'-*H*), 6.70 (dd, 1H, *J* = 8.2 Hz, *J* = 1.5 Hz, 5''-*H*), 6.63 (dd, 1H, *J* = 8.2 Hz, *J* = 1.5 Hz, 5'-*H*), 6.22-6.17 (m, 2H, 3' and 3''-*H*), 4.57 (s_{br}, 1H, O*H*), 4.10 (dd, 1H, *J* = 11.0 Hz, *J* = 9.3 Hz, 5-*H*), 3.88 (d, 1H, *J* = 11.0 Hz, 1-*H*), 3.72 (dd, 1H, *J* = 16.9 Hz, *J* = 8.1 Hz, 4-*H*), 2.67 (dd, 1H, *J* = 13.8 Hz, *J* = 8.2 Hz, *anti*-3-*H*), 2.35 (dd, 1H, *J* = 13.1 Hz, *J* = 7.6 Hz, *syn*-3-*H*), 2.17 (s, 3H, C(=O)CH_3), 1.37 (s, 3H, C(OH)CH_3).

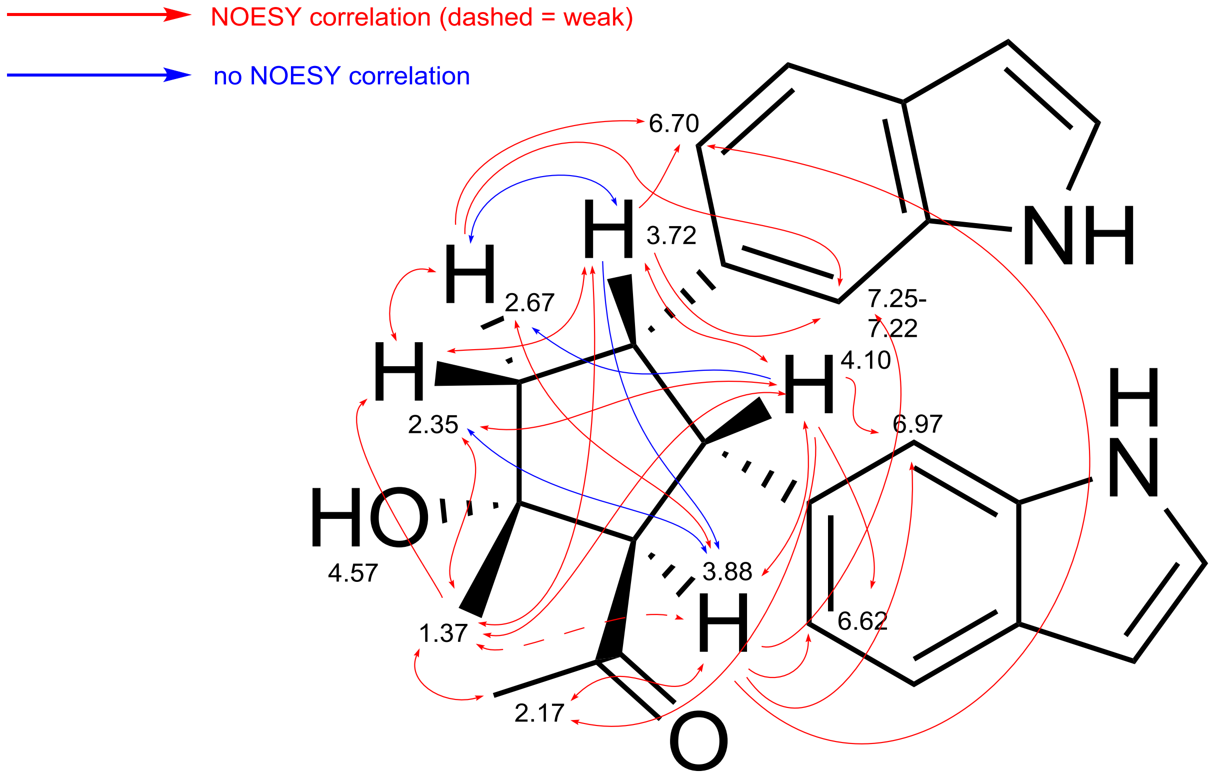

^{13}C NMR (100 MHz, acetone-d_6): δ = 208.5 (1C, *C*(=O)), 137.1 and 137.0 (2C, *C*-7a' and *C*-7a''), 136.9 (1C, *C*-6''), 135.9 (1C, C-6'), 126.9 (1C, *C*-3a'), 126.8 (1C, *C*-3a''), 124.7 and 124.5 (2C, *C*-2' and *C*-2''), 122.2 (1C, *C*-5''), 121.6 (1C, *C*-5'). 119.8 (1C, *C*-4'), 119.6 (1C, *C*-4''), 112.0 (1C, *C*-7''), 111.9 (1C, C-7'), 101.9 and 101.8 (2C, *C*-3' and *C*-3''), 79.4 (1C, *C*-1), 68.5 (1C, *C*-5), 50.8 (1C, *C*-4), 50.7 (1C, *C*-2), 47.9 (1C, *C*-3), 32.1 (1C, C(=O)CH_3), 26.4 (1C, C(OH)CH_3). IR (ATR): $\tilde{\nu}$ = 3401(m), 3101 (w), 3014 (w), 2959 (w), 2926 (w), 2856 (w), 2228 (w), 1695 (s), 1625 (w), 1510 (w), 1454 (m), 1412 (w), 1349 (s), 1263 (m), 1135 (m), 1091 (m), 1055 (m), 934 (w), 897 (m), 861 (w), 809 (s), 767 (s), 724 (s), 612 (m). UV-Vis (MeOH): λ_{max} (lg ε) = 216 (4.66), 272 (3.98), 281 (3.96), 292 (3.87). HRESIMS: calcd. for $[C_{24}H_{24}N_2O_2+Na]^+$: 395.17300, found: 395.17307 (0.18 ppm).

Compound **7**: R_t = 20.68 min. ^{1}H NMR (400 MHz, acetone-d_6): δ = 9.92-9.78 (s_{br}, 2H, 1' and 1''-N*H*), 7.17-7.13 (m, 1H, 4'-*H*), 7.15-7.11 (m, 1H, 4''-*H*), 7.08-7.05 (m, 2H, 2' and 2''-*H*), 7.07-7.03 (m, 1H, 7''-*H*), 7.03 (s, 1H, 7'-H), 6.69 (dd, 1H, *J* = 8.3 Hz, *J* = 1.6 Hz, 5'-*H*), 6.66 (dd, 1H, *J* = 8.3 Hz, *J* = 1.5 Hz, 5''-*H*), 6.22-6.18 (m, 1H, 3'-*H*), 6.19-6.15 (m, 1H, 3''-*H*), 4.39 (dd, 1H, *J* = 9.7 Hz, *J* = 9.7 Hz, 5-*H*), 4.21 (s_{br}, 1H, O*H*), 4.11 (ddd, 1H, *J* = 9.7 Hz, *J* = 9.6 Hz, *J* = 7.5 Hz, 4-*H*), 3.44 (d, 1H, *J* = 9.8 Hz, 1-*H*), 2.50 (dd, 1H, *J* = 13.5 Hz, *J* = 9.8 Hz, *anti*-3-C*H*), 2.31 (dd, 1H, *J* = 13.5 Hz, *J* = 7.4 Hz, *syn*-3-*H*), 2.06 (s, 3H, C(=O)CH_3), 1.69 (s, 3H, C(OH)CH_3).

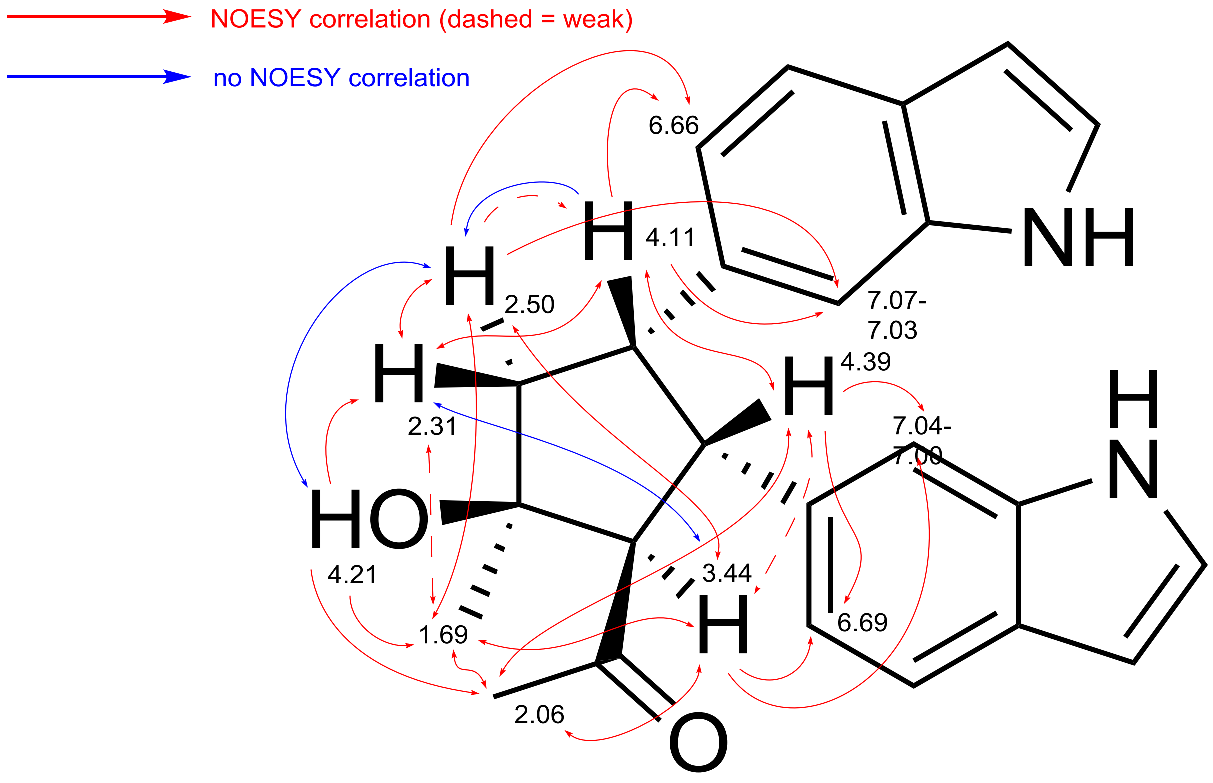

^{13}C NMR (100 MHz, acetone-d_6): δ = 210.3 (1C, *C*(=O)), 137.0 (1C, *C*-7a'') 136.9 (1C, *C*-7a'), 136.6 (1C, *C*-6''), 135.9 (1C, C-6'), 127.1 (1C, *C*-3a'), 126.9 (1C, *C*-3a''), 124.9 (1C, *C*-2'), 124.7 (1C, *C*-2''), 121.8 (1C, *C*-5''), 121.7 (1C, *C*-5'), 120.1 (1C, *C*-4'), 119.8 (1C, *C*-4''), 112.1 (1C, *C*-7'), 111.8 (1C, *C*-7''), 101.92 (1C, *C*-3''), 101.88 (1C, *C*-3'), 79.9 (1C, *C*-2), 68.3 (1C, *C*-1), 53.2 (1C, *C*-5), 49.8 (1C, *C*-3), 48.7 (1C, *C*-4), 31.3 (1C, C(=O)*C*H_3), 28.1 (1C, C(OH)*C*H_3). IR (ATR): $\tilde{\nu}$ = 3395 (w), 3015 (w),

2959 (w), 2925 (w), 2854 (w), 2228 (w), 1684 (s), 1624 (w), 1510 (w), 1454 (m), 1346 (s), 1250 (m), 1133 (m), 1090 (m), 1049 (m), 933 (m), 897 (w), 861 (m), 809 (s), 767 (s), 724 (s), 695 (s), 581 (s), 554 (s). UV-Vis (MeOH): λ_{max} (lg ε) = 216 (4.66), 272 (3.97), 281 (3.95), 292 (3.84). HRESIMS: calcd. for $[C_{24}H_{24}N_2O_2+Na]^+$: 395.17300, found: 395.17313 (0.33 ppm). Compounds **8** and **9**: R_t = 15.80 min. IR (ATR): $\tilde{\nu}$ = 3402 (m), 3014 (w), 2960 (w), 2926 (w), 2855 (w), 2228 (w), 1689 (s), 1624 (w), 1591 (w), 1511 (w), 1454 (m), 1406 (w), 1347 (s), 1252 (m), 1206 (w), 1148 (m), 1131 (m), 1091 (m), 1050 (m), 933 (w), 897 (w), 862 (w), 811 (s), 768 (s), 724 (s), 662 (m), 636 (m), 611 (m), 546 (s). UV-Vis (MeOH): λ_{max} (lg ε) = 218 (4.66), 272 (3.99), 280 (3.97), 291 (3.86). HRESIMS: calcd. for $[C_{24}H_{24}N_2O_2+Na]^+$: 395.17300, found: 395.17306 (0.15 ppm). Compound **8**: ^{1}H NMR (400 MHz, acetone-d_6): δ = 9.97 (s_{br}, 2H, 1' and 1''-N*H*), 7.40 (d, 1H, *J* = 8.1 Hz, 4'-*H*), 7.37 (d, 1H, *J* = 8.2 Hz, 4''-*H*), 7.23 (s, 1H, 7''-*H*), 7.17 (dd, 2H, *J* = 5.5 Hz, *J* = 3.0 Hz, 2' and 2''-*H*), 7.14 (s, 1H, 7'-*H*), 7.01 (dd, 1H, *J* = 8.2 Hz, *J* = 1.5 Hz, 5''-*H*), 6.96 (dd, 1H, *J* = 8.2 Hz, *J* = 1.5 Hz, 5'-*H*), 6.34-6.31 (m, 2H, 3' and 3''-*H*), 4.25 (s_{br}, 1H, O*H*), 3.98 (dd, 1H, *J* = 11.2 Hz, *J* = 11.2 Hz, 5-*H*), 3.43 (ddd, 1H, *J* = 10.8 Hz, *J* = 9.1 Hz, *J* = 9.1 Hz, 4-*H*), 3.28 (d, 1H, *J* = 11.6 Hz, 1-*H*), 2.46 (dd, 1H, *J* = 13.4 Hz, *J* = 9.4 Hz, *anti*-3-*H*), 2.21 (dd, 1H, *J* = 13.5 Hz, *J* = 8.9 Hz, *syn*-3-*H*), 1.98 (s, 3H, C(=O)CH_3), 1.59 (s, 3H, C(OH)CH_3).

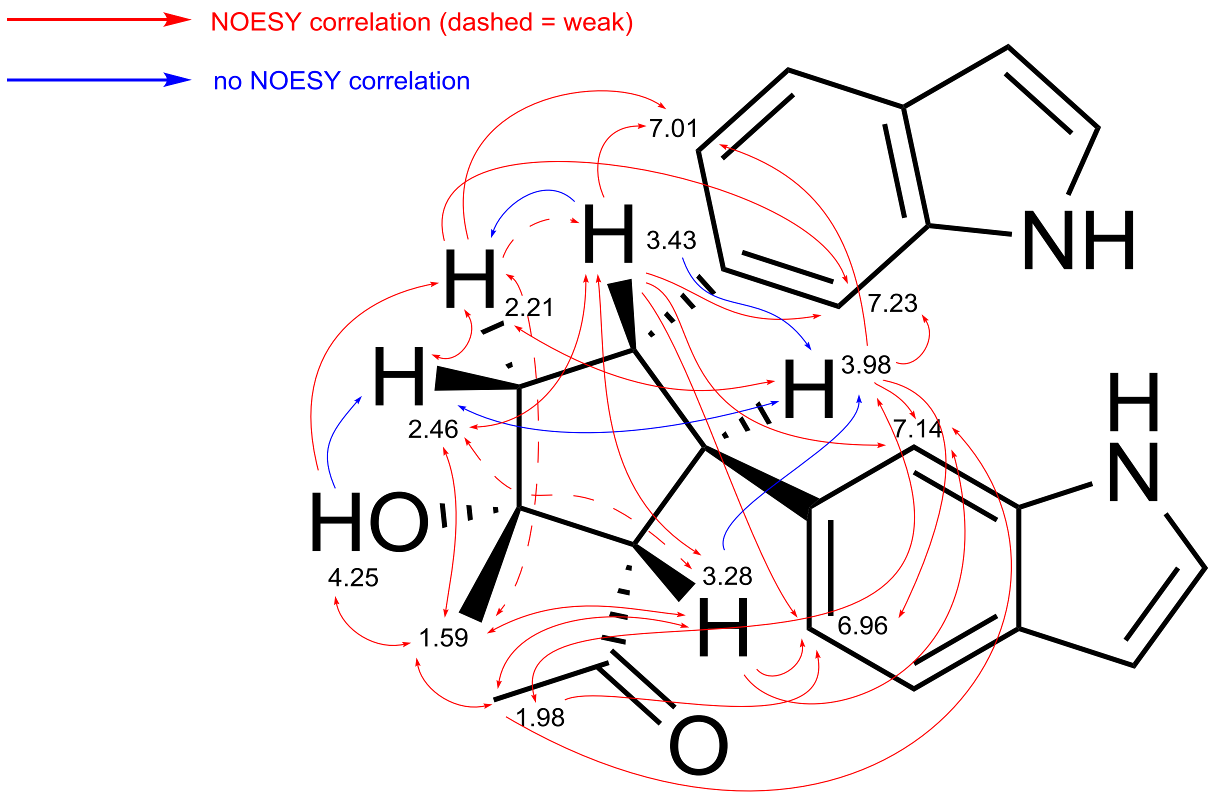

^{13}C NMR (100 MHz, acetone-d_6): δ = 210.4 (1C, *C*(=O)), 138.2 (1C, *C*-6'') 137.4 (1C, *C*-7a''), 137.3 (1C, *C*-7a'), 136.2 (1C, C-6'), 127.8 (1C, *C*-3a'), 127.5 (1C, *C*-3a''), 125.2 (1C, *C*-2''), 125.0 (1C, *C*-2'), 120.9 (1C, *C*-4'), 120.6 (1C, *C*-4''), 120.4 (1C, *C*-5''), 119.7 (1C, *C*-5'), 111.6 (1C, *C*-7'), 111.1 (1C, *C*-7''), 102.1 (1C, *C*-3'), 102.0 (1C, *C*-3''), 79.5 (1C, *C*-2), 70.3 (1C, *C*-1), 57.9 (1C, *C*-5), 52.9 (1C, *C*-4), 52.3 (1C, *C*-3), 32.3 (1C, C(=O)CH_3), 29.6 (1C, C(OH)CH_3). Compound **9**: ^{1}H NMR (400 MHz, acetone-d_6): δ = 10.18 (s_{br}, 2H, N*H*), 7.53 (d, 2H, *J* = 8.2 Hz, 4-*H*), 7.42-7.40 (m, 2H, 7-*H*), 7.29 (dd, 2H, *J* = 3.0 Hz, *J* = 2.5 Hz, 2-*H*), 7.08 (dd, 2H, *J* = 8.2 Hz, *J* = 1.5 Hz, 5-*H*), 6.43 (ddd, 2H, *J* = 3.0 Hz, *J* = 2.0 Hz, *J* = 0.9 Hz, 3-*H*), 3.51-3.47 (m, 2H, CH_2CH), 2.78 (ddd, 2H, *J* = 15.8 Hz, *J* = 7.8 Hz, *J* = 2.7 Hz, CH_2), 2.38 (dd, 2H, *J* = 15.8 Hz, *J* = 3.1 Hz, CH_2), 1.66 (s, 6H, CH_3). ^{13}C NMR (100 MHz, acetone-d_6): δ = 207.5 (2C, *C*(=O)), 137.4 (2C, *C*-7a), 137.3 (2C, *C*-6), 128.0 (2C, *C*-3a), 125.6 (2C, *C*-2), 121.1 (2C, *C*-4), 112.2 (2C, *C*-7), 102.2 (2C, *C*-3), 49.7 (2C, CH_2), 49.0 (2C, CH_2CH), 30.5 (2C, CH_3).

4-(1*H*-Indol-6-yl)-2-methylbut-3-yn-2-ol (13): Compound **4** (250 mg, 1.03 mmol, 1.0 equiv), $Pd(PPh_3)_2Cl_2$ (22 mg, 0.03 mmol, 3 mol %) and PPh_3 (8 mg, 0.03 mmol, 3 mol %) were dissolved in NEt_3 (4 mL) under inert conditions und degassed for 15 min. Separately, NEt_3 (2 mL) was degassed followed by addition of CuI (6 mg, 0.03 mmol, 3 mol %) and degassed again for 5 min. The CuI solution was added to the reaction mixture. Alkyne **12** (503 µL, 5.15 mmol, 5.0 equiv) was dissolved in NEt_3 (2 mL), degassed for 15 min and subsequently added to the reaction mixture followed by heating to 50 °C for 25 min. After completion of the reaction as monitored by TLC, the reaction mixture was filtered over Celite 545. The Celite had to be thoroughly washed with EA (50 mL). After evaporation of the solvent the crude product was subjected to column chromatography [PE/EA (3:1)] yielding the product as an orange solid (189 mg, 0.95 mmol, 92%). TLC [PE/EA (3:1)]: R_f = 0.19. Mp.: 97-98 °C. ^{1}H NMR ($CDCl_3$): δ = 8.30 (s_{br}, 1H, N*H*), 7.55 (d, 1H, *J* = 8.2 Hz, 4-*H*), 7.49-7.46 (m, 1H, 7-*H*), 7.22 (dd, 1H, *J* = 3.2 Hz, *J* = 2.5 Hz, 2-*H*), 7.17 (dd, 1H, *J* = 8.2 Hz, *J* = 1.4 Hz, 5-*H*), 6.54-6.51 (m, 1H, 3-*H*), 2.22 (s_{br}, 1H, O*H*), 1.64 (s, 6H, CH_3). ^{13}C NMR ($CDCl_3$): δ = 135.3 (1C, *C*-7a), 128.0 (1C, *C*-3a), 125.6 (1C, *C*-2,), 123.5 (1C, *C*-5), 120.5 (1C, *C*-4), 115.7 (1C, *C*-6), 114.6 (1C, *C*-7), 102.8 (1C, *C*-3), 92.1 (1C, $C(CH_3)_2C\textit{C}$), 83.6 (1C, $C(CH_3)_2\textit{C}C$), 65.8 (1C, $\textit{C}(CH_3)_2$), 31.6 (2C, $\textit{C}H_3$). IR (ATR): $\tilde{\nu}$ = 3455 (m), 3285 (m), 2976 (w), 2927 (w), 2218 (w), 1454 (w), 1402 (w), 1345 (m), 1316 (w), 1252 (m), 1223 (w), 1165 (m), 1147 (s), 1133 (s), 1062 (w), 974 (m), 941 (s), 873 (s), 854 (m), 819 (s), 797 (m), 769 (s), 740 (s), 708 (m), 664 (w), 631 (s), 579 (m), 553 (s). UV-Vis (MeOH): λ_{max} (lg ε) = 201 (4.21), 241 (4.58), 288 (4.19), 294 (4.18). MS (EI, 70 eV): *m/z* (%) = 199 $[M]^{\cdot+}$ (8), 181 $[M\text{-}H_2O]^{\cdot+}$ (100), 166 (29), 152 (10), 139 $[C_{10}H_5N]^{\cdot+}$ (14), 127 (3), 114 (4), 90 (5), 77 (8), 63 (5). HREIMS: calcd. for $[C_{13}H_{13}NO]^+$: 199.09971, found: 199.10014 (2.16 ppm).

1-(1*H*-Indol-6-yl)-3-methylbut-2-en-1-one (14): Alkyne **13** (150 mg, 0.75 mmol, 1.0 equiv) and *p*-TsOH (29 mg, 0.02 mmol, 0.2 equiv) were dissolved in ethanol (150 mL) and heated to reflux for 3 h. The reaction mixture was concentrated in vacuo, diluted with TBME (100 mL) and washed with H_2O (100 mL). The aqueous phase was further extracted with TBME (2 × 50 mL). The combined organic extracts were washed with H_2O (100 mL) and sat. $NaHCO_3$ (100 mL). After drying with $MgSO_4$ and filtration the solvent was removed in vacuo and the crude product was subjected to column chromatography with PE/EA [5:1] to give α,β-unsaturated ketone **14** as a colorless solid (112 mg, 0.56 mmol, 75%). TLC [PE/EA (3:1)]: R_f = 0.40. Mp.: 113 °C. ^{1}H NMR (400 MHz, $CDCl_3$): δ = 8.93 (s_{br}, 1H, N*H*), 8.14-8.12 (m, 1H, 7-*H*), 7.74 (dd, 1H, *J* = 8.4 Hz, *J* = 1.5 Hz, 5-*H*), 7.66 (1H, d, *J* = 8.4 Hz, 4-*H*), 7.36 (dd, 1H, *J* = 3.1 Hz, *J* = 2.6 Hz, 2-*H*), 6.83 (qq, 1H, *J* = 1.3 Hz, C*H*C(CH_3)$_2$), 6.58 (ddd, 1H, *J* = 3.0 Hz, *J* = 1.9 Hz, *J* = 0.9 Hz, 3-*H*), 2.21/2.01 (s, 6H, C(C*H*$_3$)$_2$). ^{13}C NMR (100 MHz, $CDCl_3$): δ = 192.3 (1C, *C*(=O)), 154.7 (1C, *C*(CH_3)$_2$), 135.5 (1C, *C*-7a), 133.3 (1C, *C*-6), 131.4 (1C, *C*-3a), 127.9 (1C, *C*-2), 122.0 (1C, *C*HC(CH_3)$_2$), 120.2 (1C, *C*-4), 120.1 (1C, *C*-5), 112.3 (1C, *C*-7), 102.8 (1C, *C*-3), 27.8/21.1 (2C, C(*C*H$_3$)$_2$). IR (ATR): $\tilde{\nu}$ = 3258 (s), 3193 (m), 3008 (w), 2968 (w), 2913 (w), 1647 (s), 1594 (s), 1560 (m), 1505 (m), 1440 (m), 1374 (m), 1351 (s), 1323 (m), 1285 (s), 1257 (m), 1219 (m), 1153 (s), 1134 (m), 1123 (m), 1107 (m), 1064 (m), 1027 (w), 977 (w), 928 (w), 894 (m), 876 (w), 848 (m), 815 (m), 799 (s), 769 (s), 734 (s), 716 (s), 656 (s), 599 (m), 574 (s). UV-Vis (MeOH): λ_{max} (lg ε) = 211 (4.31), 256 (4.27), 309 (4.15). MS (EI, 70 eV): *m/z* (%) = 199 $[M]^{\cdot+}$ (60), 184 $[M–(\cdot CH_3)]^+$ (100), 167 (14), 156 (17), 144 (43), 129 (6), 116 (43), 103 (1), 89 (23), 83 $[C_5H_7O]^+$ (9), 77 (3), 63 (7). HREIMS: calcd. for $[C_{13}H_{13}NO]^+$: 199.09917, found: 199.09865 (2.61 ppm).

Bisindoles 15 and 16: Samarium powder (357 mg, 2.38 mmol, 4.4 equiv) was put under HV and flushed with argon several times in a pre-dried Schlenk flask. Dry THF (10 mL) from a glove box was added. Diiodoethane (609 mg, 2.16 mmol, 4.0 equiv), which had been washed as above with a sat. $Na_2S_2O_3$ solution, was added in one portion. A vacuum was applied carefully to the mixture to remove traces of oxygen (until gas evolution from THF). After stirring for 2 h the now dark blue reaction mixture was cooled to –78 °C and the α,β-unsaturated ketone **14** (108 mg, 0.54 mmol, 1.0 equiv) in THF (2 mL) was added via a syringe pump within 30 min. The mixture was warmed to 3 °C within 15 h and then H_2O (10 mL) was added to quench the reaction (TLC showed complete consumption of the starting material). The mixture was diluted with TBME (50 mL) and washed with brine (30 mL). The aqueous phase was extracted with TBME (20 mL). The combined organic phases were washed with sat. $NaHCO_3$ and brine (20 mL each) to give the crude products. Column chromatography with PE/EA (3:1) yielded the products **15** (13 mg, 0.03 mmol, 12%) and **16** (20 mg, 0.05 mmol, 19%) as colorless oils. A fraction that contained both alcohol **15** and ketone **16** was dissolved in $CHCl_3$ (10 mL). Addition of *p*-TsOH (1 mg, 0.05 mmol) and stirring for 30 min showed complete conversion to the ketone **16**. After evaporation of the solvent the crude products was subjected to column chromatography [PE/EA (3:1)] to give ketone **16** as a colorless oil (20 mg, 0.05 mmol, 19%) which results in a combined yield of 38% for ketone **16**. Alcohol **15**: TLC [PE/EA (3:1)]: R_f = 0.15. ^{1}H NMR (600 MHz, acetone-d_6): δ = 10.61 (s_{br}, 1H, N*H'*), 10.09 (s_{br}, 1H, N*H*), 8.11-8.07 (m, 1H, 7-*H'*), 7.69-7.66 (m, 1H, 7-*H*), 7.62 (dd, 1H, *J* = 8.5 Hz, *J* = 1.6 Hz, 5-*H'*), 7.57 (dd, 1H, *J* = 8.5 Hz, *J* = 0.6 Hz, 4-*H'*), 7.56-7.55 (m, 1H, 2-*H'*), 7.46 (d, 1H, *J* = 8.4 Hz, 4-*H*), 7.24 (dd, 1H, *J* = 8.4 Hz, *J* = 1.7 Hz, 5-*H*), 7.21-7.18 (m, 1H, 2-*H*), 6.55 (s, 1H, O*H*), 6.51 (ddd, 1H, *J* = 3.0 Hz, *J* = 2.0 Hz, *J* = 0.9 Hz, 3-*H'*), 6.32 (ddd, 1H, *J* = 3.0 Hz, *J* = 2.0 Hz, *J* = 0.9 Hz, 3-*H*), 4.58 (s, 1H,

C(=O)C*H*), 2.37-2.34 (m, 2H, CH_2), 1.34 (s, 3H, CH_3), 1.25 (s, 3H, CH_3), 1.12 (s, 3H, CH_3), 0.88 (s, 3H, CH_3). ^{13}C NMR (150 MHz, acetone-d_6): δ = 207.2 (1C, *C*(=O)), 143.6 (1C, *C*-6), 137.1 (1C, *C*-7a), 136.4 (1C, *C*-7a'), 133.7 (1C, *C*-6'), 133.2 (1C, *C*-3a'), 130.5 (1C, *C*-2'), 127.3 (1C, *C*-3a), 125.5 (1C, *C*-2), 120.8 (1C, *C*-4'), 120.7 (1C, *C*-4), 120.3 (1C, *C*-5'), 117.6 (1C, *C*-5), 114.0 (1C, *C*-7'), 108.8 (1C, *C*-7), 102.8 (1C, *C*-3'), 101.9 (1C, *C*-3), 83.9 (1C, *C*OH), 62.4 (1C, C(=O)*C*H), 60.9 (1C, *C*H_2), 49.5 (1C, C(=O)CH*C*$(CH_3)_2$), 44.1 (CH_2*C*$(CH_3)_2$), 28.9 (1C, *C*H_3), 25.5 (1C, CH_3), 25.4 (1C, CH_3), 23.2 (1C, CH_3). IR (diamond-ATR): $\tilde{\nu}$ = 3360 (m), 3109 (w), 2965 (m), 2870 (w), 1705 (w), 1627 (m), 1602 (s), 1561 (w), 1503 (m), 1456 (m), 1392 (m), 1372 (s), 1349 (s), 1322 (m), 1290 (s), 1248 (m), 1223 (m), 1182 (s), 1151 (m), 1119 (m), 1093 (m), 1065 (m), 1008 (w), 990 (w), 942 (w), 899 (m), 871 (m), 821 (m), 802 (m), 767 (s), 725 (s), 643 (s), 548 (s). UV-Vis (MeOH): λ_{max} (lg ε) = 203 (4.20), 221 (4.34), 251 (3.93), 306 (3.80). HREISMS: calcd. for $[C_{26}H_{28}N_2O_2+Na]^+$: 423.20430, found: 423.20456 (0.61 ppm). Ketone **16**: TLC [PE/EA (3:1)]: R_f = 0.30. ^{1}H NMR (400 MHz, $CDCl_3$): δ = 8.86 (s_{br}, 1H, N*H*'), 8.28 (s, 1H, 7-*H*'), 7.94 (s_{br}, 1H, N*H*), 7.86 (dd, 1H, *J* = 8.4 Hz, *J* = 1.0 Hz, 5-*H*'), 7.65 (d, 1H, *J* = 8.4 Hz, 4-*H*'), 7.44 (d, 1H, *J* = 8.3 Hz, 4-*H*), 7.25 (dd, 1H, *J* = 8.4 Hz, *J* = 1.2 Hz, 5-*H*), 7.08 (s, 1H, 7-*H*), 6.97 (1, dd, *J* = 2.6 Hz, *J* = 2.6 Hz, 2-*H*'), 6.84 (dd, 1H, *J* = 2.5 Hz, *J* = 2.5 Hz, 2-*H*), 6.46-6.41 (m, 1H, 3-*H*'), 6.36-6.31 (m, 1H, 3-*H*), 6.23 (s, 1H, C(=O)CHCC*H*), 4.95 (s, 1H, C(=O)C*H*CCH), 1.24 (s, 3H, CH_3), 1.12 (s, 3H, CH_3), 1.05 (s, 3H, CH_3), 0.85 (s, 3H, CH_3). ^{13}C NMR (100 MHz, $CDCl_3$): δ = 202.5 (1C, *C*(=O)), 138.7 (1C, C(=O)CH*C*CH), 137.9 (1C, C(=O)CHC*C*H), 135.9 (1C, *C*-7a), 135.6 (1C, *C*-7a') 132.4 (1C, *C*-6'), 131.8 (1C, *C*-3a), 130.0 (1C, *C*-6), 128.8 (1C, *C*-2'), 126.9 (1C, *C*-3a), 124.6 (1C, *C*-2), 120.5 (2C, *C*-4, *C*-5'), 120.1 (1C, *C*-4'), 118.1 (1C, *C*-5), 113.0 (1C, *C*-7'), 108.2 (1C, C-7), 102.3 (1C, *C*-3'), 102.2 (1C, *C*-3), 63.9 (1C, C(=O)*C*H), 50.0 (1C,

C(=O)CH*C*$(CH_3)_2$), 47.0 (1C, C(=O)CHC$(CH_3)_2$*C*$(CH_3)_2$), 28.7 (1C, CH_3), 25.5 (1C, CH_3), 23.4 (1C, CH_3), 21.6 (1C, CH_3). IR (diamond-ATR): $\tilde{\nu}$ = 3395 (m), 3105 (w), 2960 (m), 2930 (w), 2867 (w), 1722 (w), 1651 (s), 1610 (s), 1563 (w), 1500 (m), 1454 (m), 1406 (w), 1344 (s), 1282 (m), 1252 (m), 1221 (m), 1154 (s), 1119 (m), 1096 (m), 1065 (w), 1043 (w), 996 (w), 944 (w), 898 (m), 855 (m), 805 (s), 766 (s), 725 (s), 665 (m), 633 (m), 562 (m). UV-Vis (MeOH): λ_{max} (lg ε) = 203 (4.38), 235 (4.53), 246 (4.54), 297 (4.42). HRESIMS: calcd. for $[C_{26}H_{26}N_2O+Na]^+$: 405.19373, found: 405.19385 (0.30 ppm).

Tricycles 17 and 18: A solution of unsaturated ketone **14** (50 mg, 0.25 mmol, 1.0 equiv) in 1,2-dichlorobenzene was treated with $AlCl_3$ (67 mg, 0.50 mmol, 2.0 equiv) and heated to 150 °C for 3 h under an argon atmosphere. The reaction was allowed to cool to room temperature and poured into ice water. The aqueous phase was extracted with TBME (3 × 30 mL). The organic phase was washed with sat. $NaHCO_3$, H_2O and brine (40 mL each) and dried with $MgSO_4$. After evaporation of the solvent the crude product was subjected to column chromatography to yield the products as a 0.7:1 mixture (**17**:**18**) as a yellow oil (21 mg, 0.44 mmol, 44%). TLC [PE/EA (3:1)]: R_f = 0.20. IR (diamond-ATR): $\tilde{\nu}$ = 3271 (m), 3100 (w), 2959 (m), 2923 (w), 2866 (w), 1671 (s), 1607 (s), 1572 (m), 1495 (s), 1460 (w), 1442 (w), 1423 (w), 1354 (m), 1304 (s), 1260 (m), 1217 (m), 1200 (w), 1173 (w), 1153 (w), 1107 (m), 1063 (m), 1019 (w), 953 (w), 884 (m), 813 (s), 740 (s), 700 (w), 658 (m), 626 (m), 583 (s). UV-Vis (MeOH): λ_{max} (lg ε) = 202 (3.82), 242 (4.28), 298 (3.99). Isomer **17**: ^{1}H NMR (400 MHz, $CDCl_3$): δ = 8.86 (s_{br}, 1H, N*H*), 7.77 (s, 1H, 7-*H*), 7.66 (s, 1H, 4-*H*), 7.44-7.42 (m, 1H, 2-*H*), 6.59-6.57 (m, 1H, 3-*H*), 2.68 (s, 2H, C*H*$_2$), 1.47 (s, 6H, C(C*H*$_3$)$_2$). ^{13}C NMR (100 MHz, $CDCl_3$): δ = 206.7 (1C, *C*(=O)), 154.9 (1C, *C*-5), 135.7 (1C, *C*-7a), 134.9 (1C, *C*-3a), 130.2 (1C, *C*-6), 129.6 (1C, *C*-2), 114.1 (1C, *C*-4),

105.8 (1C, *C*-7), 102.7 (1C, *C*-3), 54.1 (1C, C*H*$_2$), 38.0 (1C, *C*(CH_3)$_2$), 30.9 (2C, C(*C*H_3)$_2$). MS (EI, 70 eV): *m/z* (%) = 199 (41), 184 (100), 167 (2), 156 (21), 141 (3), 129 (16), 115 (4), 102 (2), 89 (3), 77 (5), 63 (3), 51 (2). HREIMS: calcd. for $[C_{13}H_{13}NO]^+$: 199.09917; found: 199.10083 (8.34 ppm). Isomer **18**: ^{1}H NMR (400 MHz, $CDCl_3$): δ = 9.04 (s_{br}, 1H, N*H*), 7.62 (d, 1H, *J* = 8.3 Hz, 4-*H*), 7.50 (d, 1H, *J* = 8.3 Hz, 5-*H*), 7.47-7.45 (m, 1H, 2-*H*), 6.70 (dd, 1H, *J* = 3.1 Hz, *J* = 1.9 Hz, 3-*H*), 2.69 (s, 2H, C*H*$_2$), 1.47 (s, 6H, C(C*H*$_3$)$_2$). ^{13}C NMR (100 MHz, $CDCl_3$): δ = 205.5 (1C, *C*(=O)), 148.4 (1C, *C*-7), 133.7 (1C, *C*-3a), 130.7 (1C, *C*-6), 130.6 (1C, *C*-7a), 128.0 (1C, *C*-2), 120.3 (1C, *C*-4), 114.9 (1C, *C*-5), 104.2 (1C, *C*-3), 53.5 (1C, *C*H$_2$), 38.2 (1C, *C*(CH_3)$_2$), 28.6 (2C, C(*C*H$_3$)$_2$). MS (EI, 70 eV): *m/z* (%) = 199 (52), 184 (100), 167 (2), 154 (19), 141 (4), 129 (13), 115 (5), 102 (2), 89 (3), 77 (5), 63 (3), 51 (2). HREIMS: calcd. for $[C_{13}H_{13}NO]^+$: 199.09917, found: 199.09728 (9.49 ppm).

Verticillatine B (20): 5-Bromoindole (**19**, 200 mg, 1.02 mmol, 1.0 equiv) was placed in a dry Schlenk flask under argon and dried under vacuum. THF (10 mL) was added, the solution was cooled to –78 °C and *n*-BuLi (1.6 M in hexane, 640 µL, 1.02 mmol, 1.0 equiv) was added slowly. After stirring the reaction mixture for 10 min, TBSCl (154 mg, 1.02 mmol, 1.0 equiv) was added and the solution was allowed to warm to −20 °C within 3.5 h. The reaction mixture was cooled to –78 °C again and *t*-BuLi (2.5 M in pentane, 1.20 mL, 2.04 mmol, 2.0 equiv) was added and the mixture was stirred for 15 min. After the addition an immediate change in color from colorless to yellow can be observed. *N*-isovaleroylpiperidine (182 µL, 1.02 mmol, 1.0 equiv) was added as pure oil without dilution in THF and the solution turned colorless after 20 h of stirring, while warming up to room temperature. The reaction was quenched with iPrOH (1 mL), diluted with TBME (30 mL) and washed with sat. $NaHCO_3$, H_2O and brine (30 mL each). The organic phase was dried with $MgSO_4$ and filtered. After evaporation of the solvent, the crude product was subjected to column

chromatography on silica gel [PE/EA (20:1)] to afford the TBS-protected verticillatine B (150 mg, 0.48 mmol). The protected compound was dissolved in THF (5 mL) and a solution of TBAF (1 M in THF + 5% H_2O, 480 µL, 0.48 mmol, 1.0 equiv) was added at room temperature and stirred for 1 h. The mixture was diluted with TBME (50 mL) und washed with H_2O (50 mL). After drying with $MgSO_4$, filtration and evaporation of the solvent the crude natural product was subjected to column chromatography with PE/EA [5:1, silica neutralized with PE/NEt_3 (100:1)] to afford verticillatine B as a white solid (97 mg, 0.48 mmol, 47%). TLC [PE/EA (5:1)]: R_f = 0.20. Mp.: 95-97 °C. ^{1}H NMR (400 MHz, $CDCl_3$): δ = 8.67 (s_{br}, 1H, N*H*), 8.36-8.31 (m, 1H, 4-*H*), 7.88 (dd, 1H, *J* = 8.6 Hz, *J* = 1.7 Hz, 6-*H*), 7.41 (d, 1H, *J* = 8.6 Hz, 7-*H*), 7.28 (dd, 1H, *J* = 3.1 Hz, *J* = 2.5 Hz, 2-*H*), 6.67 (ddd, 1H, *J* = 3.0, *J* = 2.0 Hz, *J* = 0.9 Hz, 3-H), 2.92 (d, 2H, *J* = 7.0 Hz, CH_2), 2.35 (tsep, 1H, *J* = 6.7 Hz, C*H*CH_2), 1.02 (d, 6H, *J* = 6.6 Hz, CH(CH_3)$_2$). ^{13}C NMR (100 MHz, $CDCl_3$): δ = 200.7 (1C, *C*(=O), 138.4 (1C, *C*-7a), 130.2 (1C, *C*-5), 127.4 (1C, *C*-3a), 125.7 (1C, *C*-2), 122.7 (1C, *C*-4), 122.3 (1C, *C*-6), 111.0 (1C, *C*-7), 104.2 (1C, *C*-3), 47.5 (1C, *C*H_2), 25.7 (1C, *C*HCH_2), 22.9 (2C, CH(*C*H_3)$_2$). IR (ATR): $\tilde{\nu}$ = 3284 (s), 2957 (m), 2927 (m), 2891 (w), 2872 (w), 1659 (s), 1601 (s), 1428 (w), 1363 (m), 1327 (m), 1298 (m), 1255 (m), 1220 (m), 1157 (m), 1117 (m), 1092 (m), 1021 (w), 912 (w), 884 (m), 797 (m), 764 (s), 731 (s), 693 (m), 646 (m), 629 (m), 575 (m), 535 (m). UV-Vis (MeOH): λ_{max} (lg ε) = 249 (4.54), 295 (3.94). MS (EI, 70 eV): *m/z* (%) = 201 (27), 186 (3), 168 (1), 159 (16), 144 (100), 130 (4), 116 (39), 103 (1), 89 (13), 77 (1), 63 (3), 51 (1). HREIMS: calcd. for $[C_{13}H_{15}NO]^+$: 201.11482, found: 201.11601 (5.92 ppm).

1-Iodo-2-methyl-3-nitrobenzene (22): 2-Methyl-3-nitroaniline (**21**, 4.85 g, 31.88 mmol, 1.0 equiv) was suspended in H_2O (33 mL) and a mixture of H_2SO_4 (6.7 mL) and H_2O (33 mL) was added. The solution was cooled to 0 °C. A solution of

$NaNO_2$ (2.31 g, 33.48 mmol, 1.1 equiv) in H_2O (33 mL) was added (the temperature had to be kept constantly below 5 °C). After stirring for 1 h there was still solid material left. Further addition of H_2SO_4 (6.7 mL) in H_2O (33 mL) and $NaNO_2$ (1.10 g, 15.94 mmol, 0.5 equiv) in H_2O (17 mL) was necessary to give the desired clear solution of the diazonium salt. KI (7.94 g, 47.82 mmol, 1.5 equiv) in H_2O (33 mL) was added dropwise and evolution of gas could be observed. After complete addition the solution was stirred for 1 h and warmed to rt. The reaction mixture was extracted four times with CH_2Cl_2 (50 mL each). The organic phase was washed with aqueous $Na_2S_2O_3$ and brine (100 mL each), dried with $MgSO_4$, and filtered. After evaporation of the solvent, the crude product was subjected to column chromatography on silica gel with PE/EA (30:1) to give a yellow solid (5.61 g, 21.33 mmol, 67%). Further purification can be done by sublimation if necessary. TLC (PE): R_f = 0.18. Mp.: 35 °C. ^{1}H NMR (400 MHz, $CDCl_3$): δ = 8.06 (dd, 1H, *J* = 7.8 Hz, *J* = 1.2 Hz, C*H*CI), 7.72 (dd, 1H, *J* = 8.0 Hz, *J* = 1.2 Hz, C*H*CNO$_2$), 7.04 (ddd, 1H, *J* = 8.0 Hz, *J* = 7.8 Hz, *J* = 1.2 Hz, C*H*CHCI), 2.60 (s, 3H, C*H*$_3$). ^{13}C NMR (100 MHz, $CDCl_3$): δ = 150.4 (1C, *C*NO$_2$), 143.1 (1C, *C*HCI), 135.0 (1C, *C*CH$_3$), 127.9 (1C, *C*HCHCI), 123.9 (1C, *C*HCNO$_2$), 103.5 (1C, *C*I), 25.0 (1C, *C*H$_3$). IR (diamond-ATR): $\tilde{\nu}$ = 3085 (w), 2968 (w), 2864 (w), 1591 (w), 1512 (s), 1442 (m), 1382 (m), 1353 (s), 1274 (w), 1205 (w), 1087 (w), 1000 (m), 911 (w), 858 (m), 793 (s), 734 (s), 693 (s), 592 (w), 552 (w). UV-Vis (MeOH): λ_{max} (lg ε) = 202 (4.24), 228 (4.14). MS (EI, 70 eV): *m/z* (%) = 262 (37), 245 (100), 217 (14), 190 (2), 164 (2), 126 (12), 119 (23), 106 (5), 90 (70), 78 (15), 63 (33), 51 (9). HREIMS: calcd. for $[M]^+$: 262.94377, found: 262.94211 (6.31 ppm).

4-Iodoindole (23): 1-Iodo-2-methyl-3-nitrobenzene (**22**, 994 mg, 3.78 mmol, 1.0 equiv) was dissolved in DMF (8.5 mL), and DMFDMA (783 µL, 5.67 mmol, 1.5 equiv) was added at rt. Then, the solution was cooled to 0 °C and pyrrolidine

(310 µL, 3.78 mmol, 1.0 equiv) was added to give a slightly orange solution. The mixture was heated to 100 °C for 2 h to give a blood red solution (at 90 °C the mixture starts to become red). Analysis by TLC showed remaining starting material. After addition of DMF (44 mL) and heating to 100 °C for 1 h the starting material was consumed. A buffer solution of NH_4OAc (5.11 g, 66.34 mmol, 17.6 equiv) in H_2O (16.6 mL) was added and the reaction mixture was cooled to 0 °C. $TiCl_3$ (20% in 3% HCl, 21.6 mL, 33.61 mmol, 8.9 equiv) was added dropwise and the solution was stirred overnight. The solution was diluted with H_2O (100 mL) and extracted with TBME (4 × 50 mL). The organic phase was washed with 2 N NaOH (50 mL), H_2O (2 × 50 mL) and brine (50 mL). After filtration and drying with $MgSO_4$ the solvent was evaporated. Sublimation in high vacuum with heating gave the product as pale yellow solid (472 mg, 1.94 mmol, 51%). Mp.: 85-90 °C. ^{1}H NMR (400 MHz, $CDCl_3$): δ = 8.32 (s_{br}, 1H, N*H*), 7.52 (dd, 1H, *J* = 7.5 Hz, *J* = 0.7 Hz, 7-*H*), 7.37 (ddd, 1H, *J* = 8.1 Hz, *J* = 0.8 Hz, *J* = 0.8 Hz, 5-*H*), 7.27 (dd, 1H, *J* = 3.1 Hz, *J* = 2.7 Hz, 2-*H*), 6.93 (dd, 1H, *J* = 8.1 Hz, *J* = 7.6 Hz, 6-*H*), 6.49 (ddd, 1H, *J* = 3.2 Hz, *J* = 2.2 Hz, *J* = 0.9 Hz, 3-*H*). ^{13}C NMR (100 MHz, $CDCl_3$): δ = 134.7 (1C, *C*-7a), 132.4 (1C, *C*-3a), 129.4 (1C, *C*-6), 124.4 (1C, *C*-2), 123.3 (1C, *C*-6), 111.0 (1C, *C*-5), 106.4 (1C, *C*-3), 87.4 (1C, *C*-4). IR (diamond-ATR): $\tilde{\nu}$ = 3377 (m), 3103 (w), 1609 (w), 1556 (m), 1502 (w), 1479 (w), 1460 (w), 1424 (m), 1410 (m), 1329 (m), 1267 (m), 1175 (m), 1138 (m), 1065 (w), 1040 (m), 990 (w), 902 (w), 880 (m), 799 (w), 777 (w), 743 (s), 720 (s), 619 (m), 562 (m). UV-Vis (MeOH): λ_{max} (lg ε) = 219 (4.59), 2.81 (3.91). MS (EI, 70 eV): *m/z* (%) = 242 (100), 214 (1), 163 (1), 126 (8), 116 (41), 89 (17), 74 (2), 63 (8), 50 (2). HREIMS: calcd. for $[M]^+$: 242.95394, found: 242.95291 (5.47 ppm).

(*E*)-4-(1*H*-Indol-4-yl)but-3-en-2-one (24): Indole **23** (100 mg, 0.41 mmol, 1.0 equiv) was placed in a sealed tube under argon with $Pd(OAc)_2$ (9 mg, 0.04 mmol,

10 mol %), methyl vinyl ketone (67 µL, 0.82 mmol, 2.0 equiv), *n*-Bu_4NBr (13 mg, 0.04 mmol, 10 mol %), NaOAc (34 mg, 0.41 mmol, 1.0 equiv) and H_2O (45 µL, 2.47 mmol, 6.0 equiv) in DMAC (2 mL). The solution was heated to 100 °C and stirred for 16 h at this temperature. After cooling to rt the mixture was filtered through a pad of Celite 545, which was thoroughly washed with EA (50 mL). The organic phase was washed with H_2O, sat. $NaHCO_3$ solution and brine (30 mL each). After drying with $MgSO_4$ the organic phase was filtered and the solvent was removed under reduced pressure. The crude product was subjected to column chromatography [PE/EA (2:1)] to yield the product as a yellow solid (58 mg, 0.31 mmol, 76%). TLC [PE/EA (2:1)]: R_f = 0.44. Mp.: 123 °C. ^{1}H NMR (400 MHz, $CDCl_3$): δ = 8.46 (s, 1H, 1-*H*), 7.94 (d, 1H, *J* = 16.3 Hz, C(=O)CHC*H*), 7.47 (ddd, 1H, *J* = 8.1 Hz, *J* = 0.8 Hz, *J* = 0.8 Hz, 7-*H*), 7.40 (ddd, 1H, *J* = 7.5 Hz, *J* = 0.7 Hz, *J* = 0.7 Hz, 5-*H*), 7.34 (dd, 1H, *J* = 3.1 Hz, *J* = 2.7 Hz, 2-*H*), 7.22 (dd, 1H, *J* = 7.9 Hz, *J* = 7.7 Hz, 6-*H*), 6.93 (d, 1H, *J* = 16.3 Hz, C(=O)C*H*), 6.84 (ddd, 1H, *J* = 3.2 Hz, *J* = 2.0 Hz, *J* = 1.0 Hz, 3-*H*), 2.44 (s, 3H, CH_3). ^{13}C NMR (100 MHz, $CDCl_3$): δ = 198.8 (1C, *C*(=O)), 142.5 (1C, C(=O)CH*C*H), 136.3 (1C, *C*-7a), 127.4 (1C, C(=O)*C*H), 127.1 (1C, *C*-3a), 126.6 (1C, *C*-4), 125.7 (1C, *C*-2), 122.1 (1C, *C*-6), 120.8 (1C, *C*-5), 113.5 (1C, *C*-7), 101.3 (1C, *C*-3), 27.5 (1C, *C*H_3). IR (diamond-ATR): $\tilde{\nu}$ = 3243 (m), 3102 (w), 2996 (w), 2923 (w), 1755 (w), 1658 (m), 1630 (s), 1499 (w), 1436 (m), 1417 (m), 1344 (s), 1263 (s), 1206 (m), 1158 (m), 1132 (w), 1092 (w), 1054 (w), 1019 (w), 996 (w), 965 (s), 897 (m), 841 (w), 743 (s), 703 (m), 619 (w), 586 (m), 561 (m), 542 (m). UV-Vis (MeOH): λ_{max} (lg ε) = 211 (4.41), 252 (4.15), 365 (4.10). MS (EI, 70 eV): *m/z* (%) = 185 (81), 170 (100), 154 (4), 142 (25), 128 (4), 115 (87), 102 (2), 89 (13), 63 (9), 57 (3). HREIMS: calcd. for $[M]^+$: 185.08352, found: 185.08327 (1.35 ppm).

4-((1*E*,3*E*)-4-Chloro-3-methylbuta-1,3-dien-1-yl)-1*H*-indole and 4-((1*E*,3*Z*)-4-chloro-3-methylbuta-1,3-dien-1-yl)-1*H*-indole (25): Chloromethyltriphenylphosphonium chloride (118 mg, 0.34 mmol, 2.2 equiv) and unsaturated ketone **24** (30 mg, 0.16 mmol, 1.0 equiv) were dried under high vacuum in separate Schlenk flasks. The phosphonium salt was suspended in THF (2 mL). After cooling to 0 °C a 1.9 M solution of PhLi in Bu_2O (190 µL, 0.35 mmol, 2.2 equiv) and a 1 M solution of LiBr in THF (350 µL, 0.35 mmol, 2.2 equiv) were added at the same time. The mixture was warmed to rt. The phosphonium salt did not dissolve completely and further PhLi (40 µL, 0.08 mmol, 0.5 equiv) was added but the solid still did not dissolve. The mixture was cooled to –78 °C and the unsaturated ketone **24** in THF (2 mL) was added dropwise. The reaction mixture turned turbid and was warmed to rt and again cooled to –78 °C (stirring for 5 min at each temperature). At −78 °C a second portion of PhLi (190 µL, 0.36 mmol, 2.3 equiv) and LiBr (350 µL, 0.35 mmol, 2.2 equiv) was added and the reaction mixture was stirred for 15 min, warmed to rt (15 min stirring) and again cooled to –78 °C (15 min stirring). At this temperature a 2 M solution of HCl in Et_2O (160 µL, 0.32 mmol, 2.0 equiv) was added dropwise and the solution was almost completely decolorized. KO*t*-Bu (23 mg, 0.19 mmol, 1.2 equiv) was added in one portion and the reaction was warmed to rt. After stirring for 1 h the TLC showed complete consumption of the starting material. The mixture was diluted with TBME (10 mL) and quenched with H_2O (5 mL). TMBE (10 mL) was added and the organic phase was washed with H_2O (50 mL) followed by extraction of the aqueous phase with TBME (10 mL). The combined organic phases were washed with brine (30 mL) and dried with $MgSO_4$. After filtration the crude product was subjected to column chromatography on silica gel (60–200 µm) that had been previously neutralized with PE/EA (5:1) + 1% NEt_3. Elution with the same solvent mixture gave the products as a yellow oil (*E*:*Z*/1:0.63, 32 mg, 0.15 mmol,

92%). TLC [PE/EA (5:1)]: R_f = 0.39. IR (diamond-ATR): $\tilde{\nu}$ = 3412 (m), 2951 (w), 2919 (w), 2854 (w), 1602 (w), 1572 (w), 1502 (w), 1431 (w), 1411 (w), 1381 (w), 1339 (m), 1279 (w), 1160 (w), 1111 (w), 1081 (w), 1023 (w), 959 (m), 882 (w), 792 (m), 747 (s), 719 (m), 598 (w), 567 (w), 539 (w). UV-Vis (MeOH): λ_{max} (lg ε) = 213 (4.35), 245 (4.17), 292 (3.91), 333 (4.03). HRESIMS: calcd. for $[M+H]^+$: 218.07310, found: 218.07321 (0.50 ppm). *E*-isomer: ^{1}H NMR (400 MHz, $CDCl_3$): δ = 10.4 (s_{br}, 1H, N*H*), 7.40-7.39 (m, 1H, 2-*H*), 7.37 (ddd, 1H, *J* = 8.0 Hz, *J* = 0.8 Hz, *J* = 0.8 Hz, 7-*H*), 7.25 (dd, 1H, *J* = 7.4 Hz, *J* = 0.9 Hz, 5-*H*), 7.16-7.12 (m, 2H, C*H*C*H*CCH$_3$), 7.12-7.08 (m, 1H, 6-*H*), 6.82 (ddd, 1H, *J* = 3.1 Hz, *J* = 2.0 Hz, *J* = 1.0 Hz, 3-*H*), 6.53 (q, 1H, *J* = 1.3 Hz, C*H*Cl), 2.11 (d, 3H, *J* = 1.3 Hz, C*H*$_3$). ^{13}C NMR (100 MHz, $CDCl_3$): δ = 138.7 (1C, *C*CH$_3$), 137.7 (1C, *C*-7a), 129.7 (1C, *C*-4), 129.0 (1C, CH*C*HCCH$_3$), 128.7 (1C, *C*HCHCCH$_3$), 127.4 (1C, *C*-3a), 126.1 (1C, *C*-2), 122.25 (1C, *C*-6), 119.7 (1C, *C*HCl), 117.9 (1C, *C*-5), 111.9 (1C, *C*-7), 101.0 (1C, *C*-3), 12.9 (1C, *C*H$_3$). *Z*-isomer: ^{1}H NMR (400 MHz, $CDCl_3$): δ = 10.5 (s_{br}, 1H, N*H*), 7.59 (dd, 1H, *J* = 16.3 Hz, *J* = 0.8 Hz, CHC*H*CCH$_3$), 7.45-7.43 (m, 1H, 2-*H*), 7.42 (d, 1H, *J* = 8.1 Hz, 5-*H*), 7.30 (ddd, 1H, *J* = 7.3 Hz, *J* = 0.8 Hz, *J* = 0.8 Hz, 7-*H*), 7.22 (d, 1H, *J* = 16.3 Hz, C*H*CHCCH$_3$), 7.16-7.12 (m, 1H, 6-*H*), 6.83 (ddd, 1H, *J* = 3.1 Hz, *J* = 2.0 Hz, *J* = 0.8 Hz, 3-*H*), 6.22-6.19 (m, 1H, C*H*Cl), 2.08 (d, 1H, *J* = 1.5 Hz, C*H*$_3$). ^{13}C NMR (100 MHz, $CDCl_3$): δ = 137.8 (1C, *C*-7a), 135.9 (1C, *C*CH$_3$), 132.0 (1C, *C*HCHCCH$_3$), 129.6 (1C, *C*-4), 127.2 (1C, *C*-3a), 126.4 (1C, *C*-2), 124.4 (1C, CH*C*HCCH$_3$), 122.28 (1C, *C*-6), 119.0 (1C, *C*-7), 115.7 (1C, *C*HCl), 112.5 (1C, *C*-5), 101.1 (1C, *C*-3), 17.9 (1C, *C*H$_3$).

Indiacen B (2) and *Z*-isomer 26: DMF (1 mL) was cooled to 0 °C. $POCl_3$ (14 µL, 0.15 mmol, 2.1 equiv) was added and the solution was stirred for 30 min. Indole **25** (15 mg, 0.07 mmol, 1.0 equiv) in DMF (1 mL) was added dropwise. After stirring for

16 h some starting material still remained, and a 0.15 M solution of $POCl_3$ in DMF (140 µL, 0.02 mmol, 0.3 equiv) was added. The mixture was poured into ice-cold sat. $NaHCO_3$ (25 mL). The aqueous phase was extracted with EA three times (10 mL each). The combined organic phases were washed with H_2O and brine (30 mL each) and dried with $MgSO_4$. After filtration the solvent was evaporated to give the crude product as a brown solid (14 mg). The *E*-isomer **2** (3.8 mg, 0.02 mmol, 29%) and *Z*-isomer **26** (2.7 mg, 0.01 mmol, 14%) of the natural product were separated by semi-preparative HPLC (250 × 10 mm column, packed with LiChrosorb Si 100, 5 µm, flow: 4.6 mL/min) under isocratic conditions [*n*-hexane/EA (70:30)]. Indiacen B (**2**): R_t = 69.0 min. ^1H NMR (400 MHz, acetone-d_6): δ = 11.37 (s_{br}, 1H, N*H*), 9.91 (s, 1H, C*H*O), 8.63 (d, 1H, *J* = 16.0 Hz, C*H*CHCCH$_3$), 8.32 (d, 1H, *J* = 2.8 Hz, 2-*H*), 7.59 (d, 1H, *J* = 7.6 Hz, 5-*H*), 7.46 (dd, 1H, *J* = 8.0 Hz, *J* = 0.9 Hz, 7-*H*), 7.28 (ddd, 1H, *J* = 8.0 Hz, *J* = 7.6 Hz, *J* = 0.4 Hz, 6-*H*), 6.97 (dd, 1H, *J* = 16.0 Hz, *J* = 0.6 Hz, CHC*H*CCH$_3$), 6.49-6.46 (m, 1H, C*H*Cl), 2.20 (d, 3H, *J* = 1.3 Hz, C*H*$_3$). ^{13}C NMR (100 MHz, acetone-d_6): δ = 184.4 (1C, *C*HO), 143.0 (1C, *C*-2), 140.1 (1C, *C*-7a), 139.5 (1C, *C*CH$_3$), 132.6 (1C, *C*-4), 131.7 (1C, *C*HCHCCH$_3$), 128.7 (1C, CH*C*HCCH$_3$), 125.0 (1C, *C*-6), 124.0 (1C, *C*-3a), 121.6 (1C, *C*-3), 119.3 (1C, *C*HCl), 118.9 (1C, *C*-5), 112.5 (1C, *C*-7), 13.3 (1C, *C*H$_3$). IR (diamond-ATR): $\tilde{\nu}$ = 3166 (m), 3113 (m), 3068 (m), 3001 (w), 2956 (m), 2916 (m), 2846 (w), 2765 (w), 1633 (s), 1567 (m), 1511 (m), 1457 (m), 1422 (m), 1401 (s), 1359 (m), 1334 (m), 1296 (m), 1274 (m), 1171 (w), 1147 (m), 1103 (m), 1056 (w), 1010 (w), 975 (s), 887 (m), 853 (m), 794 (s), 770 (s), 744 (s), 642 (s), 614 (s), 598 (m). UV-Vis (MeOH): λ_{max} (lg ε) = 209 (4.32), 224 (4.35), 235 (4.35), 320 (4.39). HREIMS: calcd. for $[C_{14}H_{12}ClNO+Na]^+$: 268.04996, found: 268.05007 (0.41 ppm). *Z*-indiacen B (**26**): R_t = 78.8 min. ^1H NMR (400 MHz, $CDCl_3$): δ = 11.44 (s_{br}, 1H, N*H*), 9.91 (s, 1H, C*H*O), 8.74 (dd, 1H, *J* = 16.1 Hz, *J* = 0.5 Hz, C*H*CHCCH$_3$),

8.33 (d, 1H, *J* = 3.3 Hz, 2-*H*), 7.67 (d, 1H, *J* = 7.6 Hz, 5-*H*), 7.51 (dd, 1H, *J* = 8.0 Hz, *J* = 0.9 Hz, 7-*H*), 7.39 (dd, 1H, *J* = 16.2 Hz, *J* = 0.8 Hz, CHC*H*CCH_3), 7.33 (ddd, 1H, *J* = 8.1 Hz, *J* = 7.6 Hz, *J* = 0.5 Hz, 6-*H*), 6.19-6.17 (m, 1H, C*H*Cl), 2.18 (d, 3H, *J* = 1.5 Hz, CH_3). ^{13}C NMR (100 MHz, $CDCl_3$): δ = 184.4 (1C, *C*HO), 143.2 (1C, *C*-2), 140.1 (1C, *C*-7a), 136.7 (1C, *C*CH_3), 134.7 (1C, *C*HCHCCH_3), 132.5 (1C, *C*-4), 125.1 (1C, *C*-6), 124.1 (1C, *C*-3a), 123.9 (1C, CH*C*HCCH_3), 121.6 (1C, *C*-3), 119.4 (1C, *C*-5), 115.2 (1C, *C*HCl), 113.0 (1C, *C*-7), 18.5 (1C, *C*H_3). IR (diamond-ATR): $\tilde{\nu}$ = 3162 (m), 3109 (m), 3005 (m), 2958 (m), 2916 (m), 2751 (w), 1638 (s), 1512 (m), 1454 (m), 1398 (s), 1363 (m), 1344 (m), 1295 (m), 1267 (m), 1146 (m), 1104 (m), 1058 (m), 1012 (m), 969 (m), 888 (w), 851 (m), 770 (s), 734 (s), 645 (s), 618 (m), 598 (m), 565 (m), 542 (w). UV-Vis (MeOH): λ_{max} (lg ε) = 203 (4.46), 320 (4.25). HREIMS: calcd. for $[C_{14}H_{12}ClNO+Na]^+$: 268.04996, found: 268.05011 (0.56 ppm).

2. Crystallographic figures and tables for indiacen B (2)

Crystal data: $C_{14}H_{12}ClNO$, orthorhombic, $P2_12_12_1$, a = 6.34592(9), b = 12.84125(15), c = 14.30104(16) Å, V = 2122.71(15) Å^3, Z = 4, T = 100 K, D_x = 1.400 Mg m^{-3}. A colorless irregular crystal ca. 0.35 × 0.2 × 0.1 mm was used to record a total of 28202 data to 2θ(max) 151° using Cu Kα radiation on an Oxford Diffraction Nova A diffractometer. Absorption corrections were based on multi-scans. The structure was refined on F^2 using the program SHELXL-97 to $wR2$ 0.064 (all data), $R1$ 0.024 ($I > 2\sigma(I)$) for 2392 independent data and 159 parameters; S = 1.08, max $\Delta\rho$ = 0.19 e Å^{-3}. The NH hydrogen was refined freely, other hydrogen atoms were included using a riding model or rigid methyl groups. The Flack parameter refined to 0.006(11), but the compound is achiral. Crystallographic data have been deposited with the Cambridge Crystallographic Data Centre as supplementary publications no. CCDC-1038862.

Copies of the data can be obtained free of charge from www.ccdc.cam.ac.uk/data_request/cif.

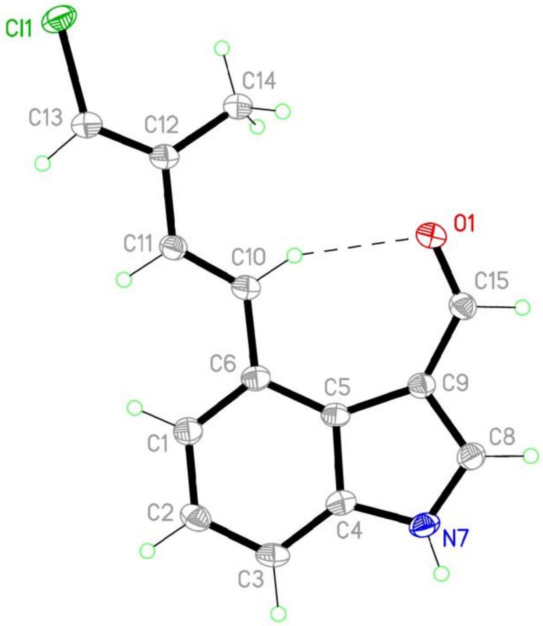

Figure S1: Ellipsoid representation of compound **2**, showing the intramolecular hydrogen bond C10–H···O1.

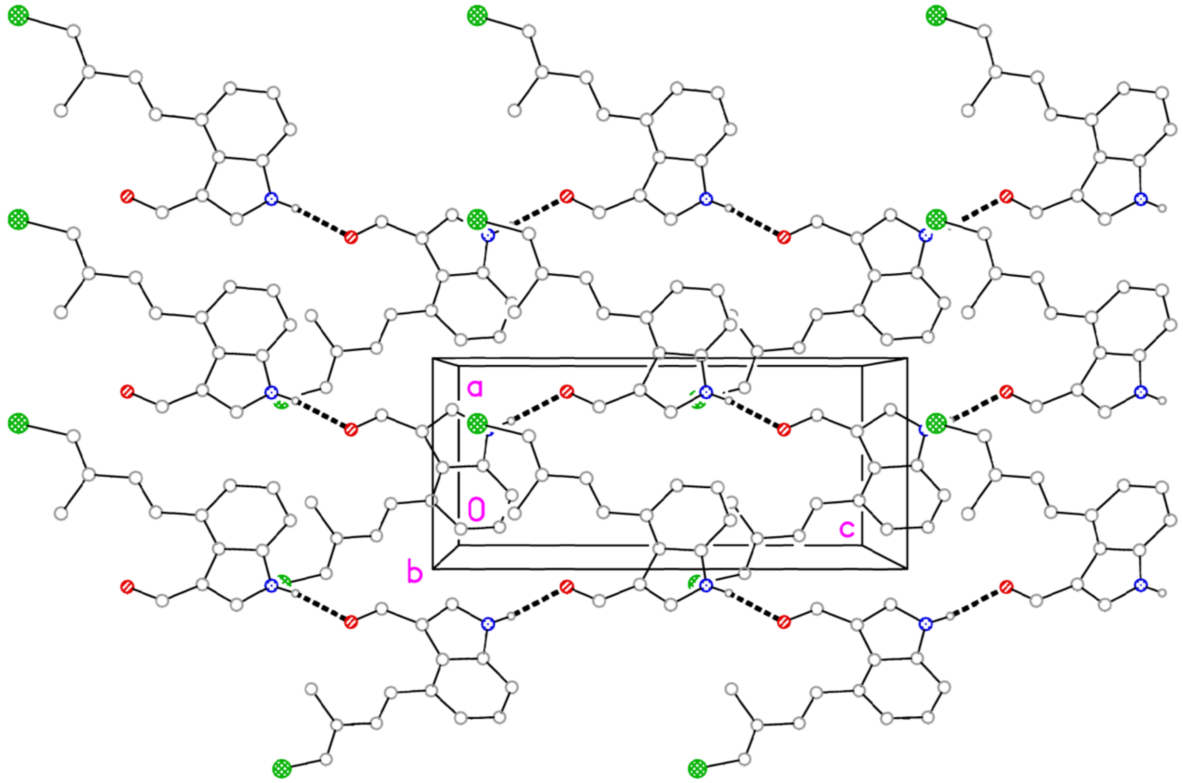

Figure S2: Packing diagram of compound **2**, showing the formation of chains parallel to the c axis via the classical hydrogen bond N7–H···O1.

Crystallographic tables: NB This information is also available, together with full experimental details, in the deposited CIF file.

Table 1: Atomic coordinates (×10^4) and equivalent isotropic displacement parameters (Å^2×10^3). U(eq) is defined as one third of the trace of the orthogonalized U_{ij} tensor.

Atom	x	y	z	U(eq)
C(1)	3740(2)	6450.8(11)	5238.5(9)	21.9(3)
C(2)	3608(2)	6105.8(11)	6163.4(9)	23.7(3)
C(3)	1897(2)	5534.8(10)	6471.4(9)	22.8(3)
C(4)	338(2)	5318.8(10)	5817.0(10)	19.6(3)
C(5)	462(2)	5612.2(10)	4862.0(9)	17.7(3)
C(6)	2219(2)	6207.1(10)	4564.3(9)	19.0(3)
N(7)	–1533.8(19)	4783.8(9)	5954.2(8)	22.0(2)
C(8)	–2564(2)	4708.1(10)	5139.4(10)	22.0(3)
C(9)	–1431(2)	5184.6(10)	4424.5(9)	18.7(3)
C(10)	2409(2)	6561.6(10)	3591.4(9)	19.7(3)
C(11)	4144(2)	6985.2(11)	3216.7(9)	19.8(3)
C(12)	4325(2)	7324.6(10)	2241.3(9)	19.4(3)
C(13)	6249(2)	7603.5(11)	1957.9(9)	21.6(3)
C(14)	2403(2)	7341.8(12)	1634.0(10)	24.8(3)
C(15)	–2274(2)	5128.8(10)	3494.5(9)	20.1(3)
O(1)	–1519.6(16)	5470.0(8)	2759.8(7)	23.9(2)
Cl(1)	6881.6(5)	8042.0(3)	847.5(2)	27.6(1)

Table 2: Bond lengths [Å].

Atoms	Distance	Atoms	Distance
C(1)-C(2)	1.3974(19)	N(7)-C(8)	1.3397(18)
C(1)-C(6)	1.3998(19)	C(8)-C(9)	1.3916(19)
C(2)-C(3)	1.382(2)	C(9)-C(15)	1.4355(18)
C(3)-C(4)	1.390(2)	C(10)-C(11)	1.3398(19)
C(4)-N(7)	1.3863(17)	C(11)-C(12)	1.4659(17)
C(4)-C(5)	1.4189(18)	C(12)-C(13)	1.336(2)
C(5)-C(6)	1.4169(19)	C(12)-C(14)	1.4973(19)

C(5)-C(9)	1.4616(19)	C(13)-Cl(1)	1.7321(14)
C(6)-C(10)	1.4689(17)	C(15)-O(1)	1.2351(17)

Table 3: Bond angles [°].

Atoms	Angle	Atoms	Angle
C(2)-C(1)-C(6)	122.66(13)	C(8)-N(7)-C(4)	109.33(12)
C(3)-C(2)-C(1)	121.14(13)	N(7)-C(8)-C(9)	110.79(12)
C(2)-C(3)-C(4)	116.77(12)	C(8)-C(9)-C(15)	117.78(12)
N(7)-C(4)-C(3)	127.85(13)	C(8)-C(9)-C(5)	105.99(11)
N(7)-C(4)-C(5)	108.39(12)	C(15)-C(9)-C(5)	136.20(12)
C(3)-C(4)-C(5)	123.75(13)	C(11)-C(10)-C(6)	124.90(13)
C(6)-C(5)-C(4)	118.43(12)	C(10)-C(11)-C(12)	124.44(12)
C(6)-C(5)-C(9)	136.11(12)	C(13)-C(12)-C(11)	116.10(12)
C(4)-C(5)-C(9)	105.46(11)	C(13)-C(12)-C(14)	124.39(12)
C(1)-C(6)-C(5)	117.13(12)	C(11)-C(12)-C(14)	119.51(12)
C(1)-C(6)-C(10)	121.77(12)	C(12)-C(13)-Cl(1)	125.26(11)
C(5)-C(6)-C(10)	121.09(12)	O(1)-C(15)-C(9)	128.76(13)

Table 4: Torsion angles [°].

Atoms	Angle	Atoms	Angle
(6)-C(1)-C(2)-C(3)	–2.8(2)	C(4)-N(7)-C(8)-C(9)	0.13(16)
C(1)-C(2)-C(3)-C(4)	0.4(2)	N(7)-C(8)-C(9)-C(15)	177.10(12)
C(2)-C(3)-C(4)-N(7)	–178.59(13)	N(7)-C(8)-C(9)-C(5)	–1.35(16)
C(2)-C(3)-C(4)-C(5)	2.7(2)	C(6)-C(5)-C(9)-C(8)	–177.48(14)
N(7)-C(4)-C(5)-C(6)	177.62(11)	C(4)-C(5)-C(9)-C(8)	2.00(14)
C(3)-C(4)-C(5)-C(6)	–3.47(19)	C(6)-C(5)-C(9)-C(15)	4.5(3)
N(7)-C(4)-C(5)-C(9)	–1.97(14)	C(4)-C(5)-C(9)-C(15)	–176.02(15)
C(3)-C(4)-C(5)-C(9)	176.94(12)	C(1)-C(6)-C(10)-C(11)	11.5(2)
C(2)-C(1)-C(6)-C(5)	1.98(19)	C(5)-C(6)-C(10)-C(11)	–169.31(13)
C(2)-C(1)-C(6)-C(10)	–178.84(13)	C(6)-C(10)-C(11)-C(12)	179.20(12)
C(4)-C(5)-C(6)-C(1)	1.02(18)	C(10)-C(11)-C(12)-C(13)	–171.63(14)
C(9)-C(5)-C(6)-C(1)	–179.55(14)	C(10)-C(11)-C(12)-C(14)	8.5(2)
C(4)-C(5)-C(6)-C(10)	–178.17(12)	C(11)-C(12)-C(13)-Cl(1)	–179.46(10)
C(9)-C(5)-C(6)-C(10)	1.3(2)	C(14)-C(12)-C(13)-Cl(1)	0.4(2)

C(3)-C(4)-N(7)-C(8)	–177.64(13)	C(8)-C(9)-C(15)-O(1)	–178.12(14)
C(5)-C(4)-N(7)-C(8)	1.20(15)	C(5)-C(9)-C(15)-O(1)	–0.3(3)

Table 5: Hydrogen bonds [Å and °].

D-H...A	d(D-H)	d(H...A)	d(D...A)	<(DHA)
N(7)-H(07)...O(1)#1	0.85(2)	2.06(2)	2.8809(15)	162.8(19)
C(14)-H(14A)...Cl(1)#2	0.98	2.83	3.5980(14)	135.5
C(8)-H(8)...Cl(1)#3	0.95	2.87	3.7519(14)	155.4
C(10)-H(10)...O(1)	0.95	2.26	3.0976(17)	147.0

Symmetry transformations used to generate equivalent atoms:
#1 -*x*-1/2,-*y*+1,*z*+1/2; #2 *x*-1/2,-*y*+3/2,-*z*; #3 -*x*,*y*-1/2,-*z*+1/2

3. Antimicrobial activity of indiacen B (2)

Table 6: Comparison of antimicrobial activities of both synthetic and natural indiacen B (**2**).

	MIC values (µg/mL)	
Microbial species	Indiacen B synthetic	Indiacen B isolated
Arthrobacter rubellus	not tested	0.8
Nocardioides simplex	1.7	3.3
Escherichia coli TOL C	8.3	33.0
Mycobacterium lacticola	not tested	33.0
Mycobacterium sp.	8.3	not tested
Mycobacterium diernhoferi	no inhibition	>33.0
Chromobacterium violaceum	67.0	33.0
Pseudomonas stutzeri	no inhibition	33.0
Mucor hiemalis	8.3	16.6

4. ^{1}H and ^{13}C NMR spectra and HPLC chromatograms

^{1}H NMR ($CDCl_3$, 400 MHz)

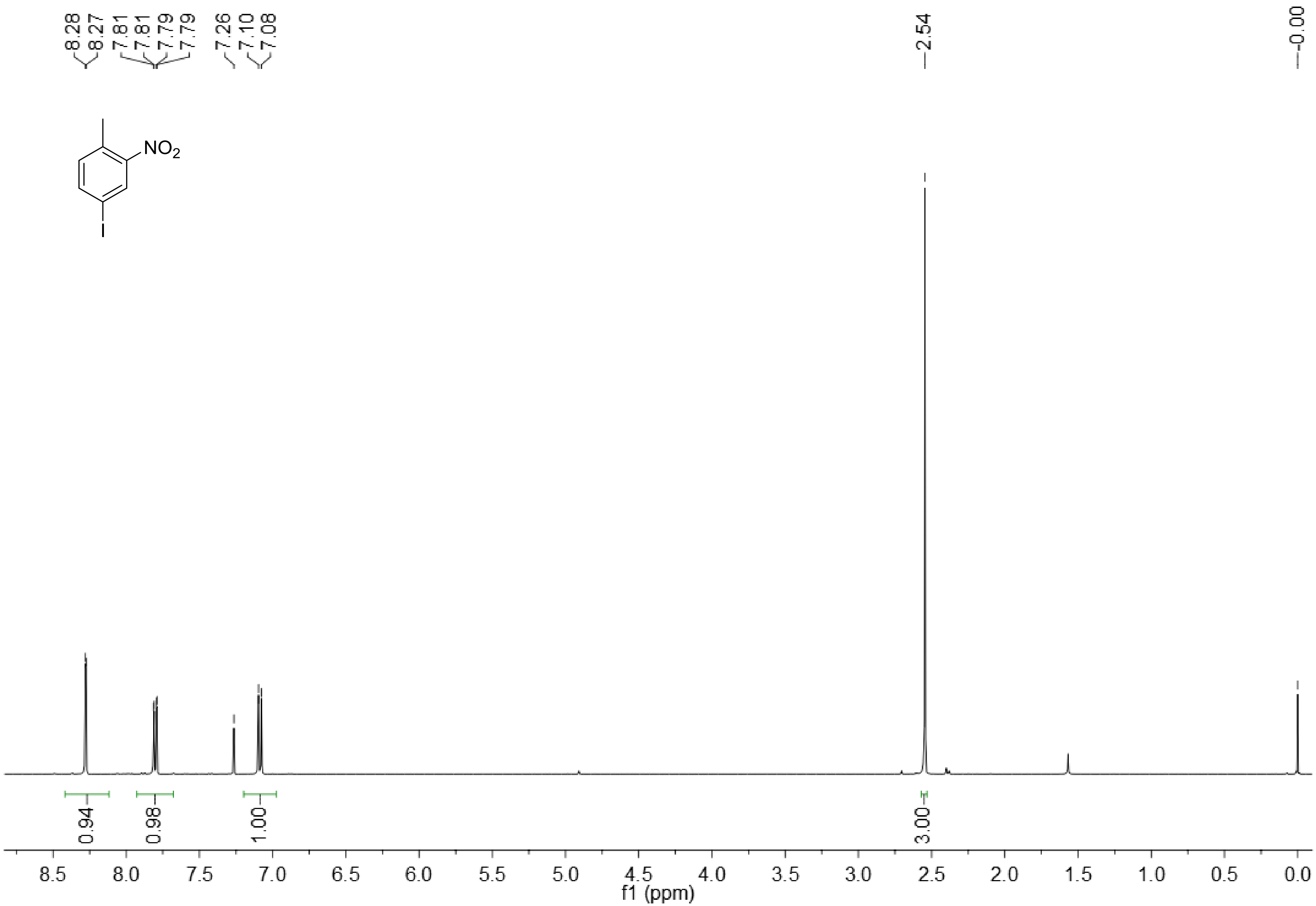

^{13}C NMR ($CDCl_3$, 100 MHz)

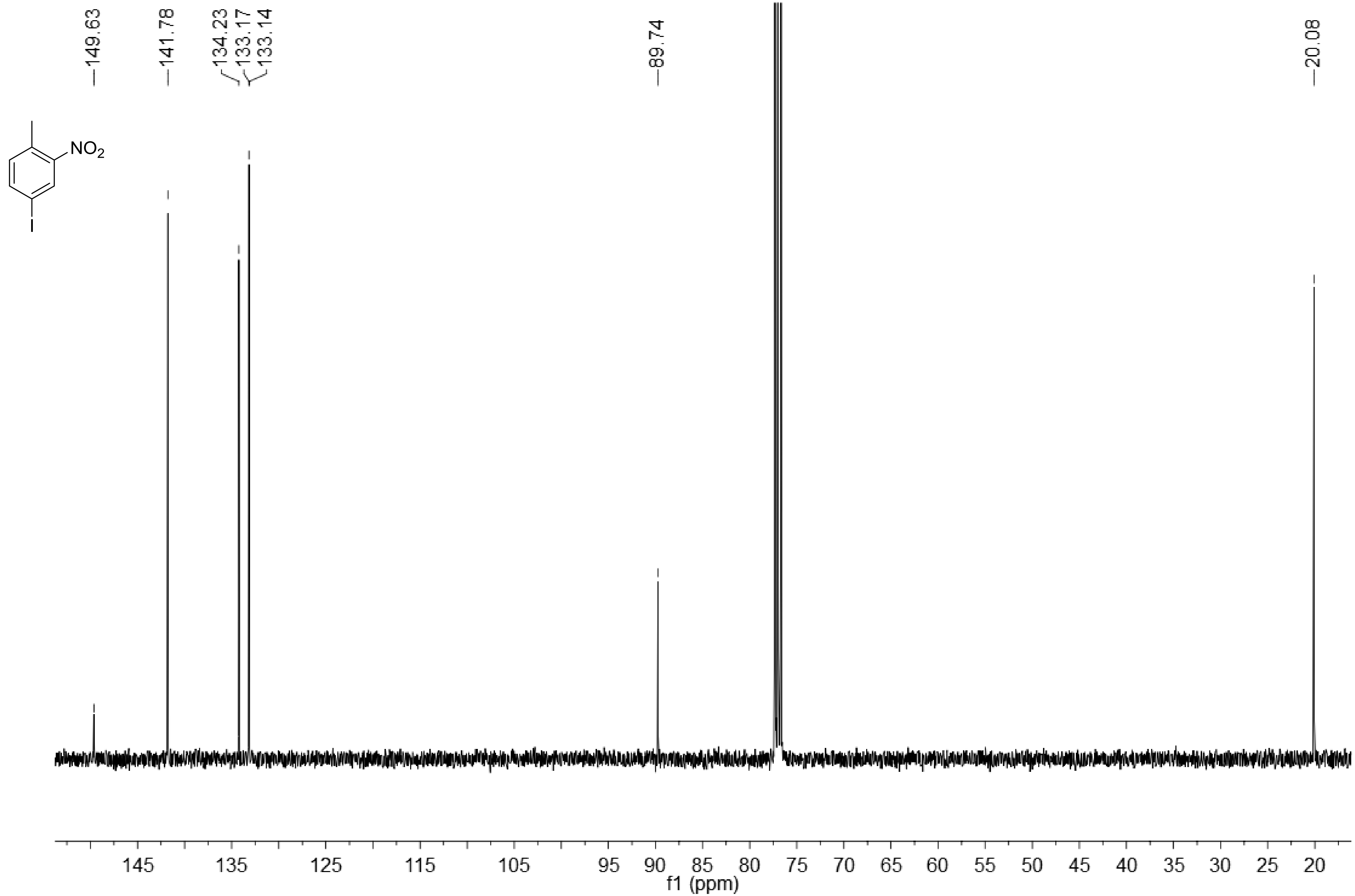

^{1}H NMR ($CDCl_3$, 400 MHz)

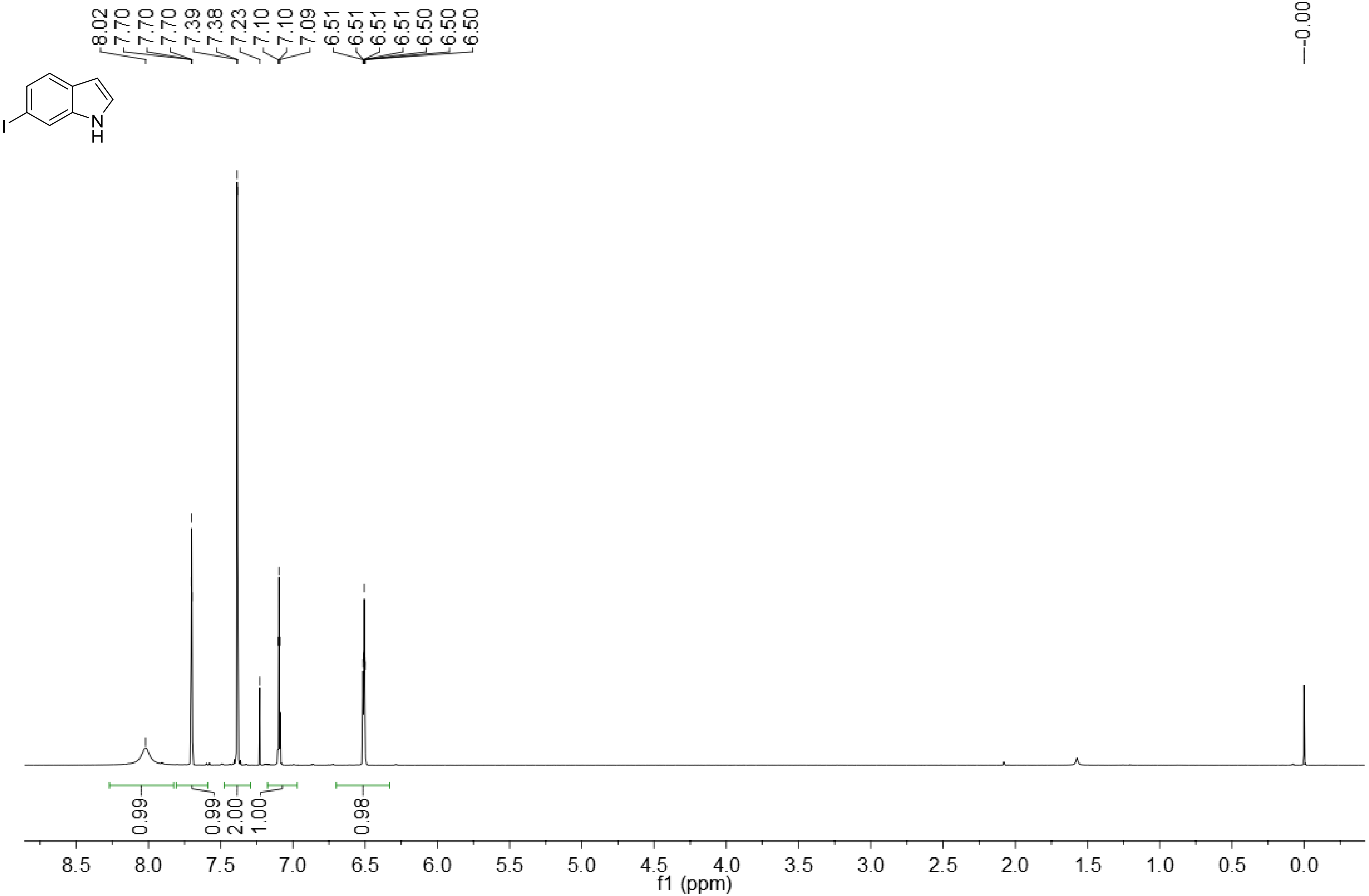

^{13}C NMR ($CDCl_3$, 100 MHz)

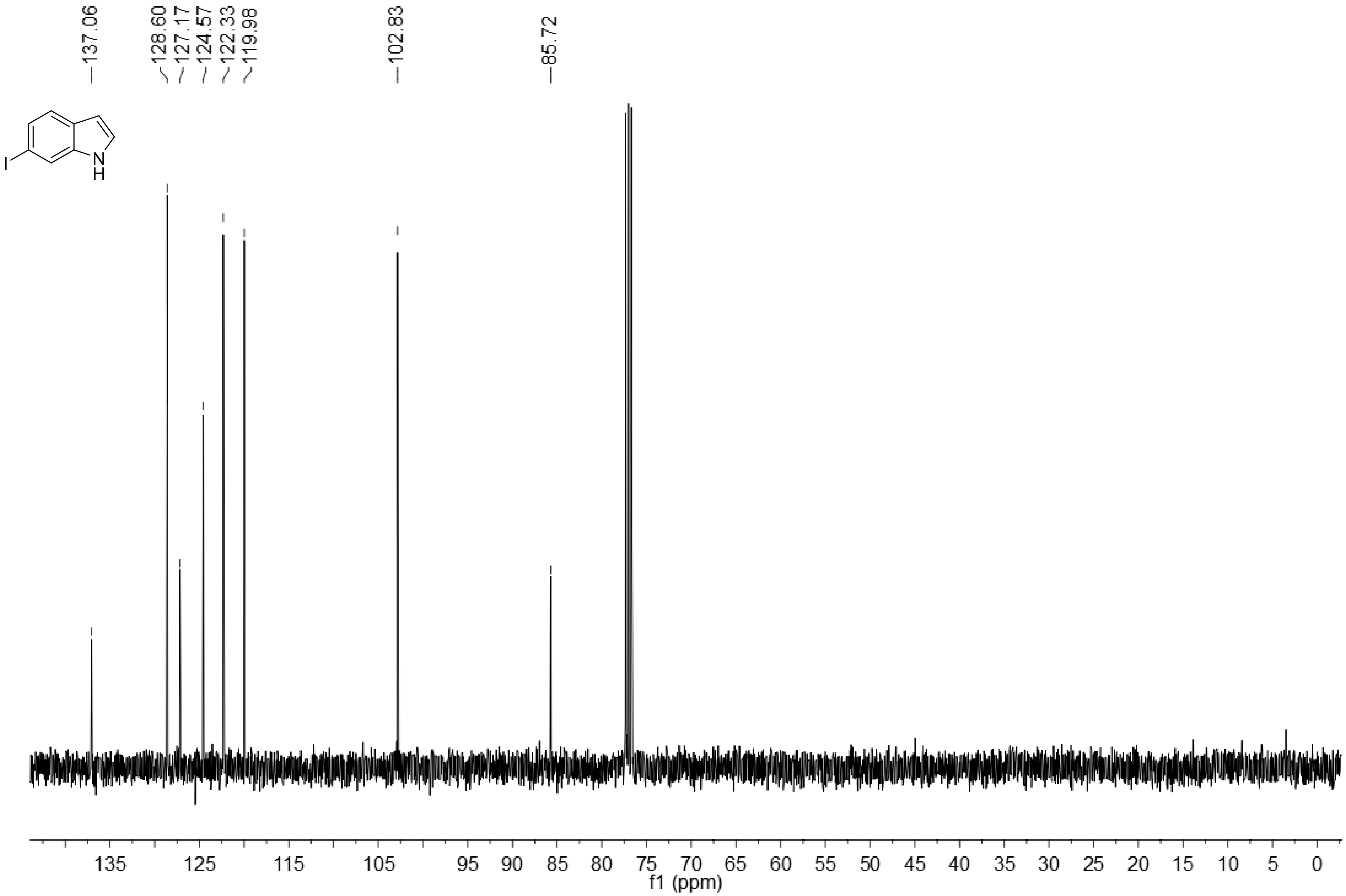

^1H NMR ($CDCl_3$, 400 MHz)

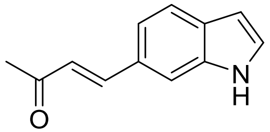

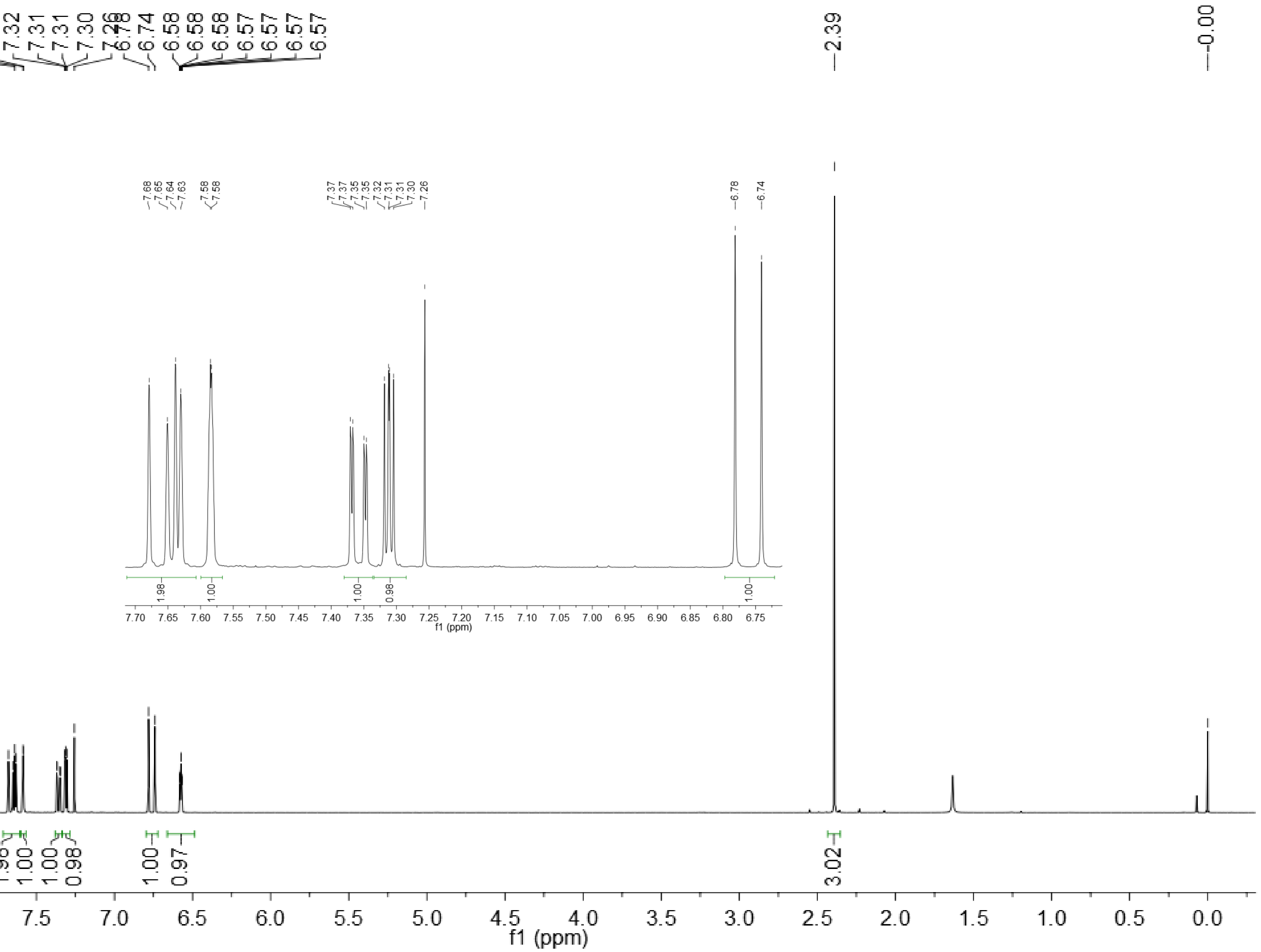

^{13}C NMR ($CDCl_3$, 100 MHz)

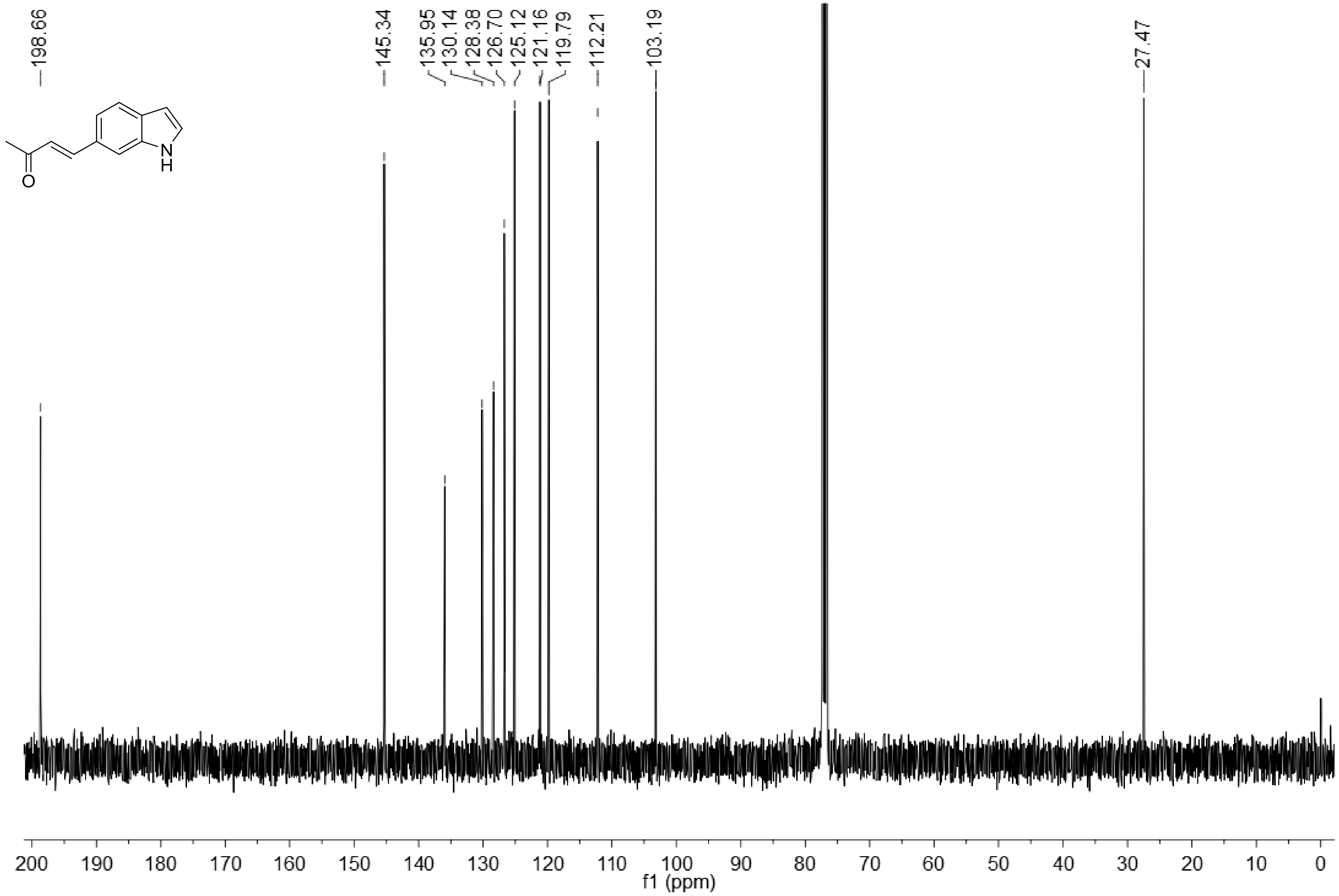

HPLC chromatogram of the separation of cyclopentyl indoles

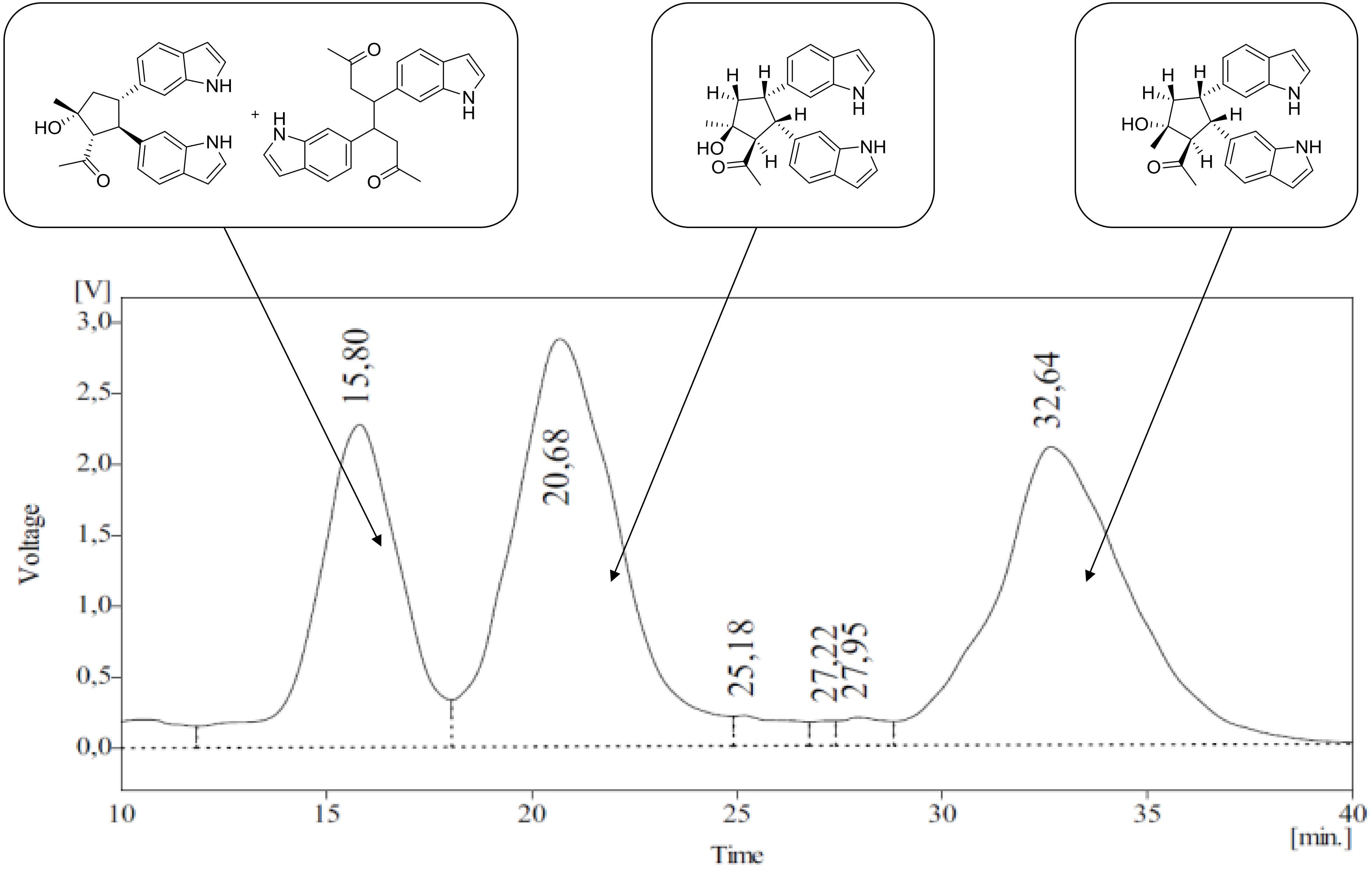

^{1}H NMR (acetone-d_6, 400 MHz)

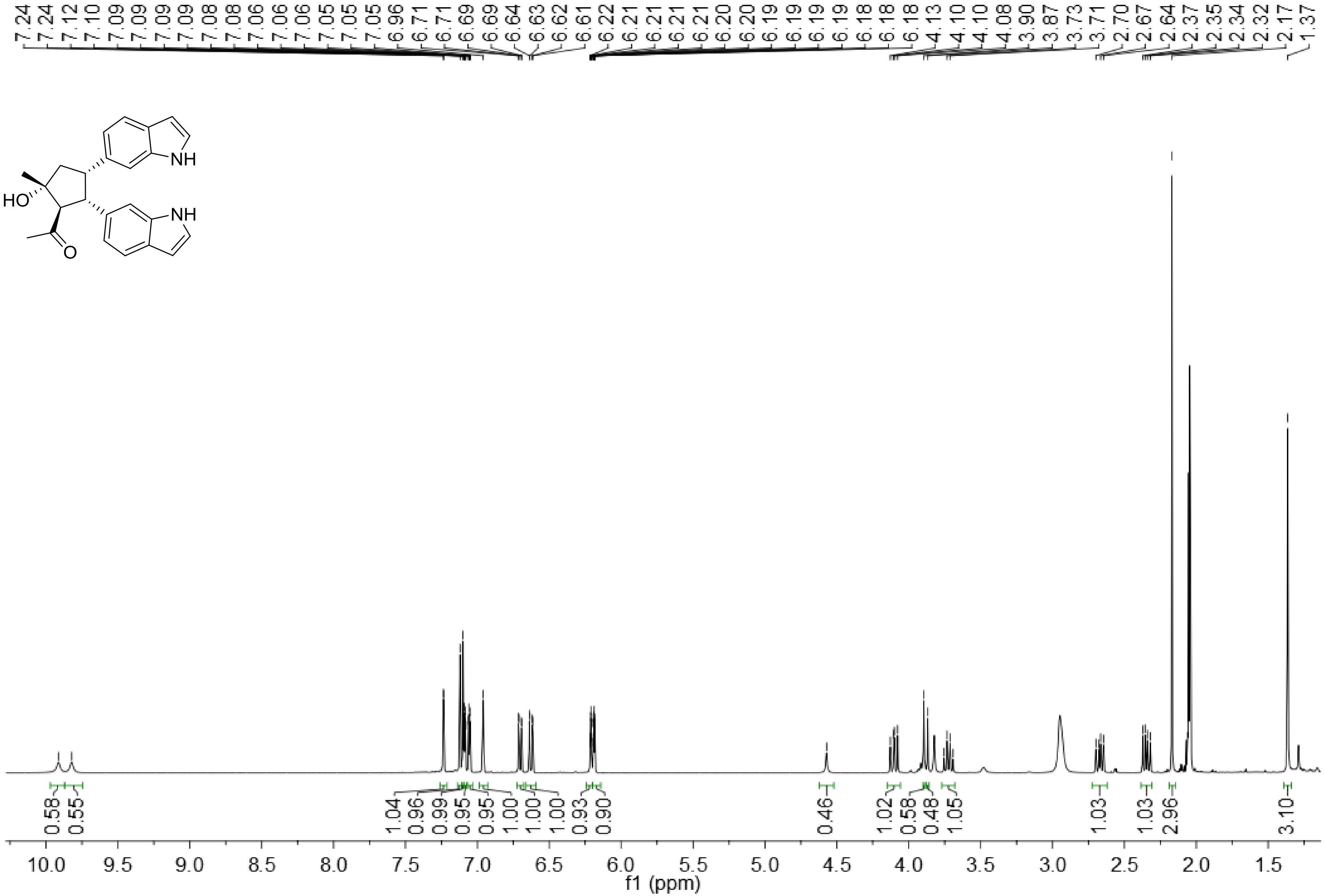

^{13}C NMR (acetone-d_6, 100 MHz)

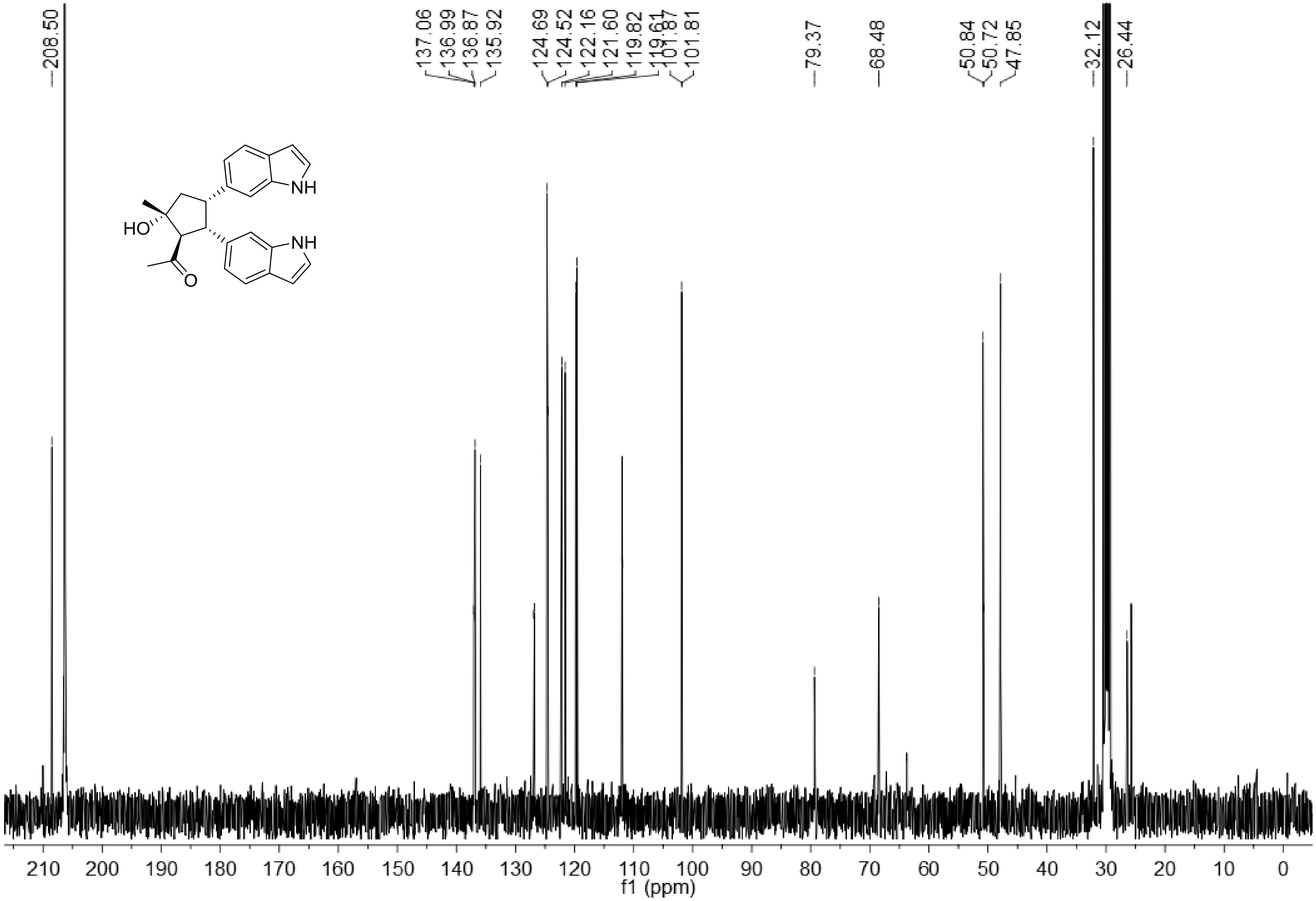

^{1}H NMR (acetone-d_6, 400 MHz)

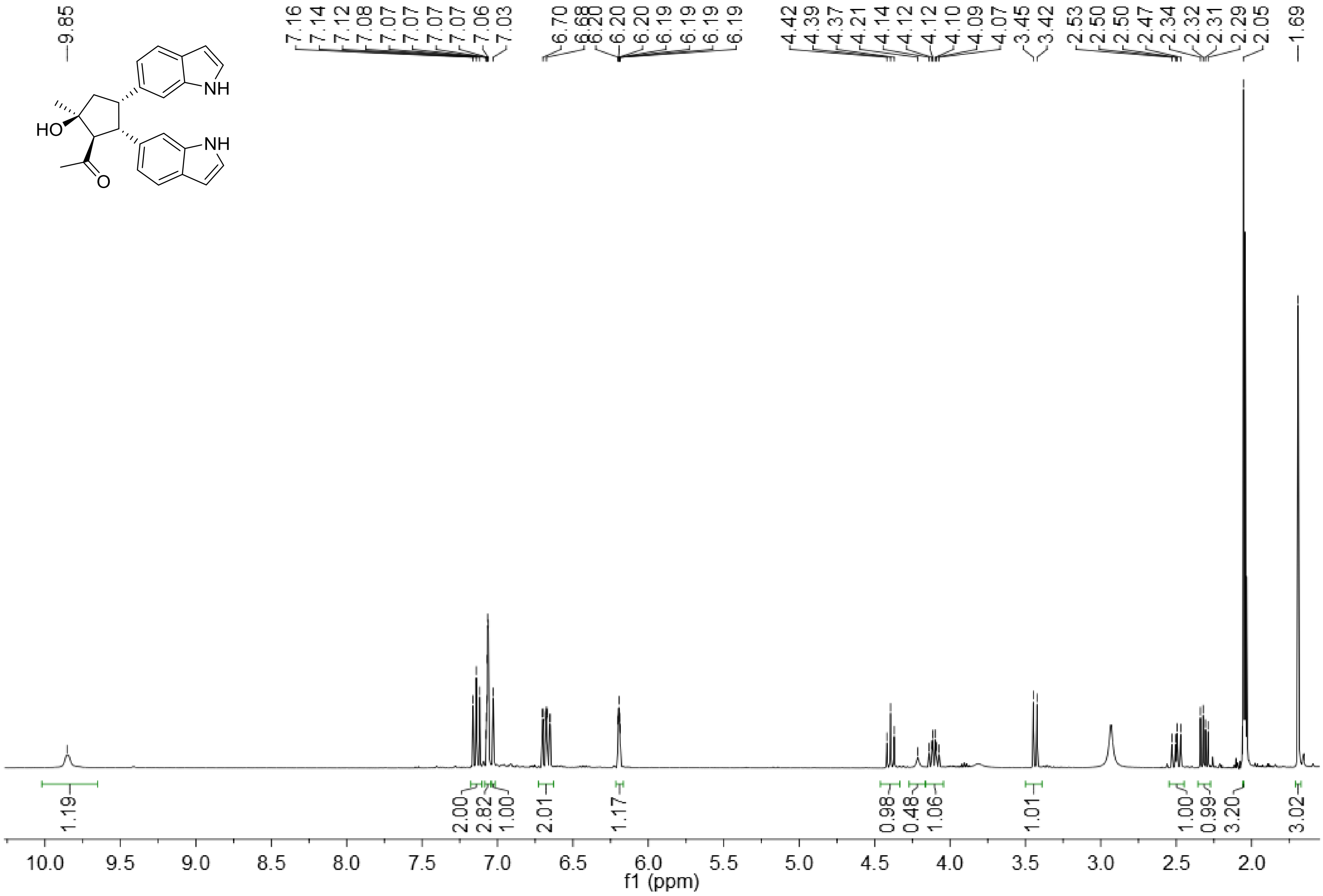

^{13}C NMR (acetone-d_6, 100 MHz)

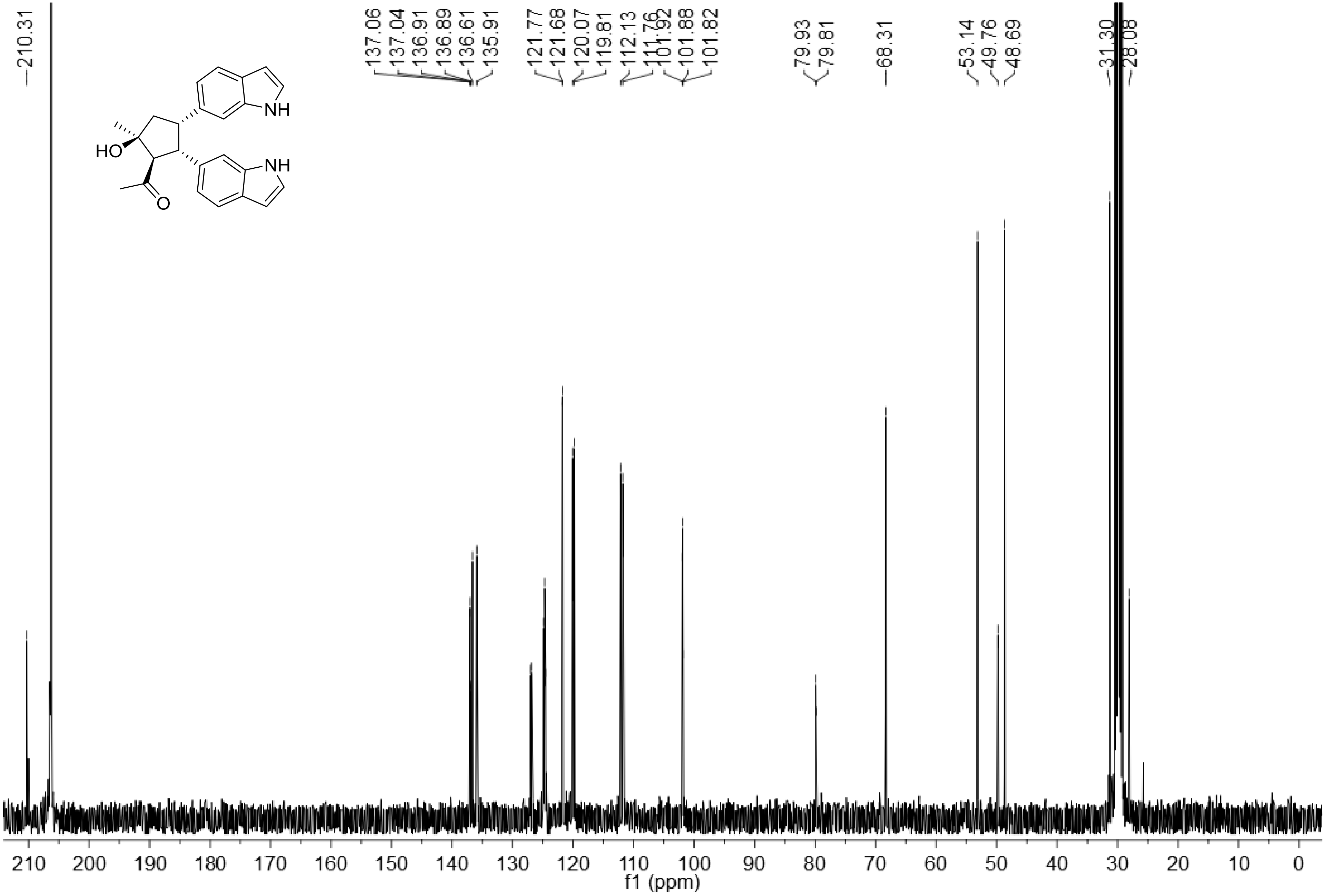

^{1}H NMR (acetone-d_6, 400 MHz)

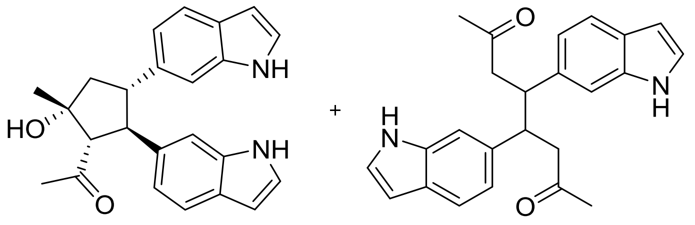

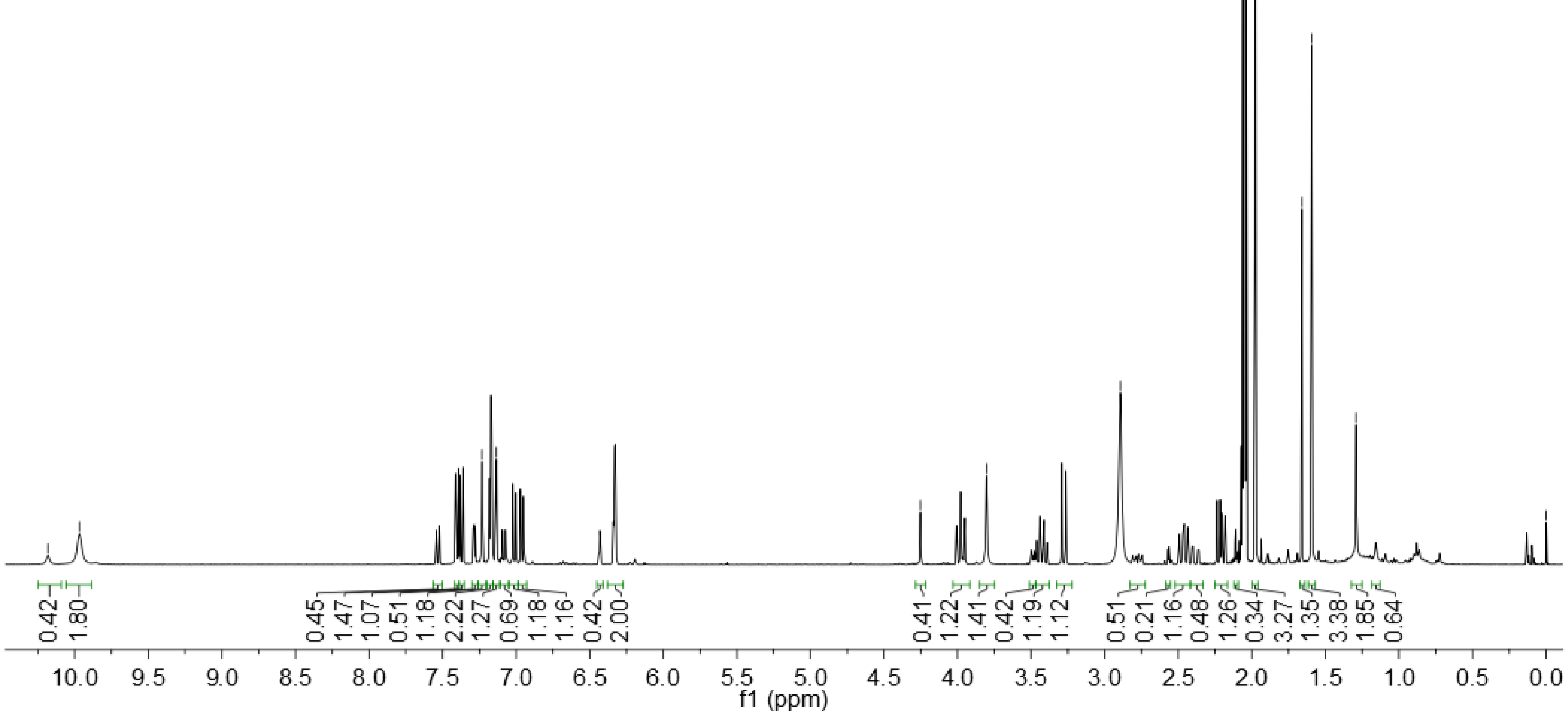

^{13}C NMR (acetone-d_6, 100 MHz)

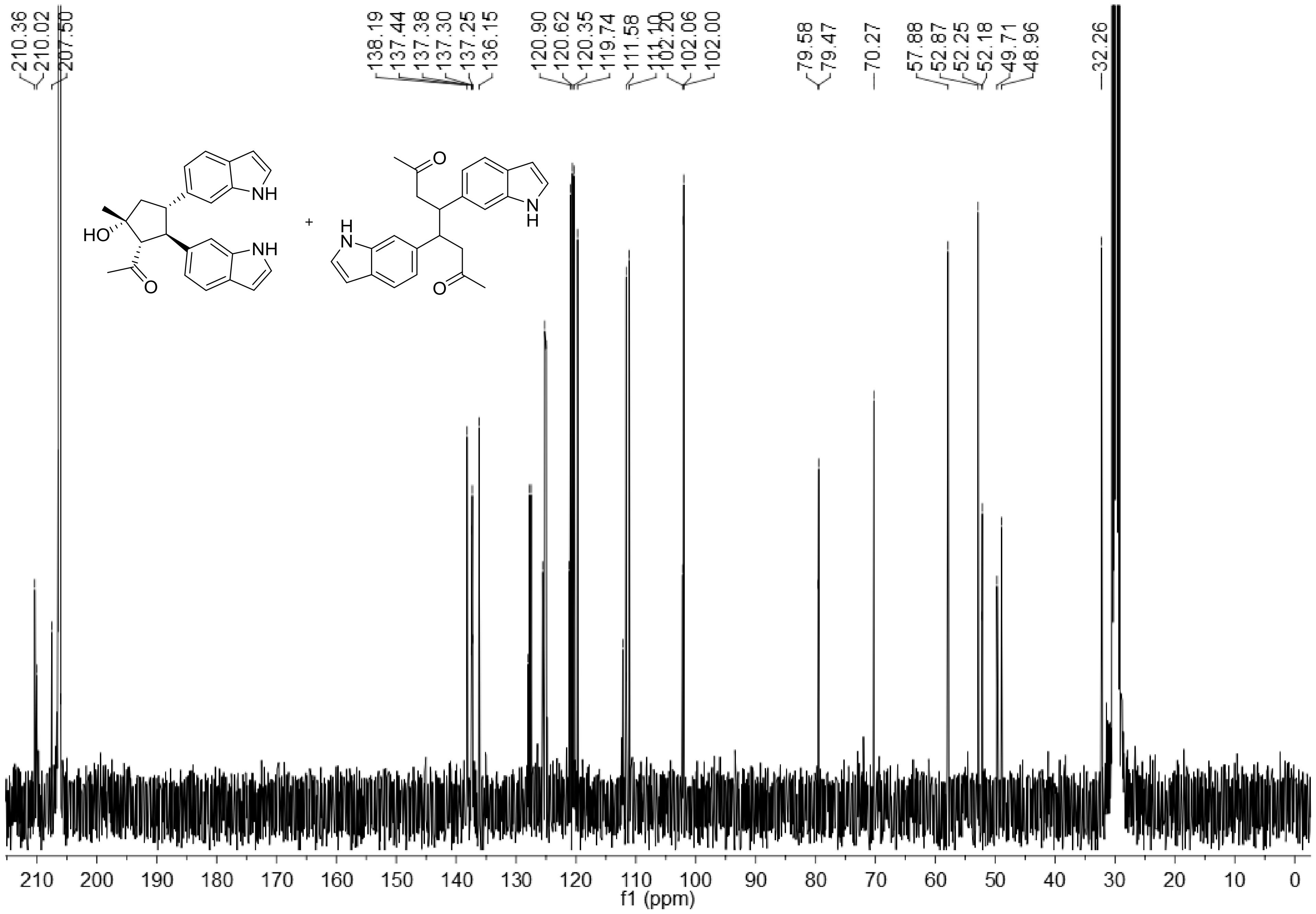

^{1}H NMR ($CDCl_3$, 400 MHz)

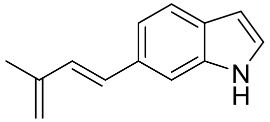

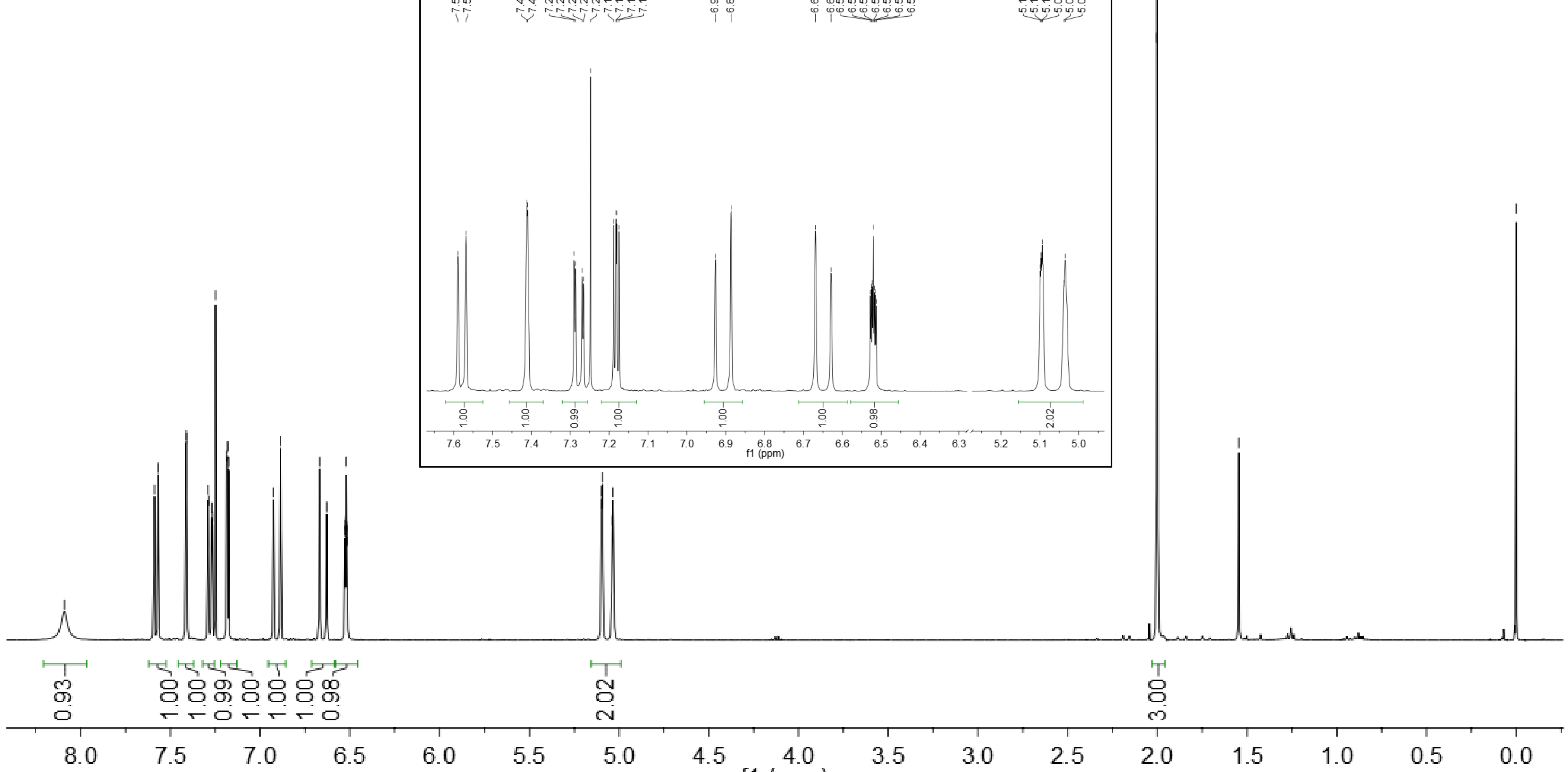

^{13}C NMR ($CDCl_3$, 100 MHz)

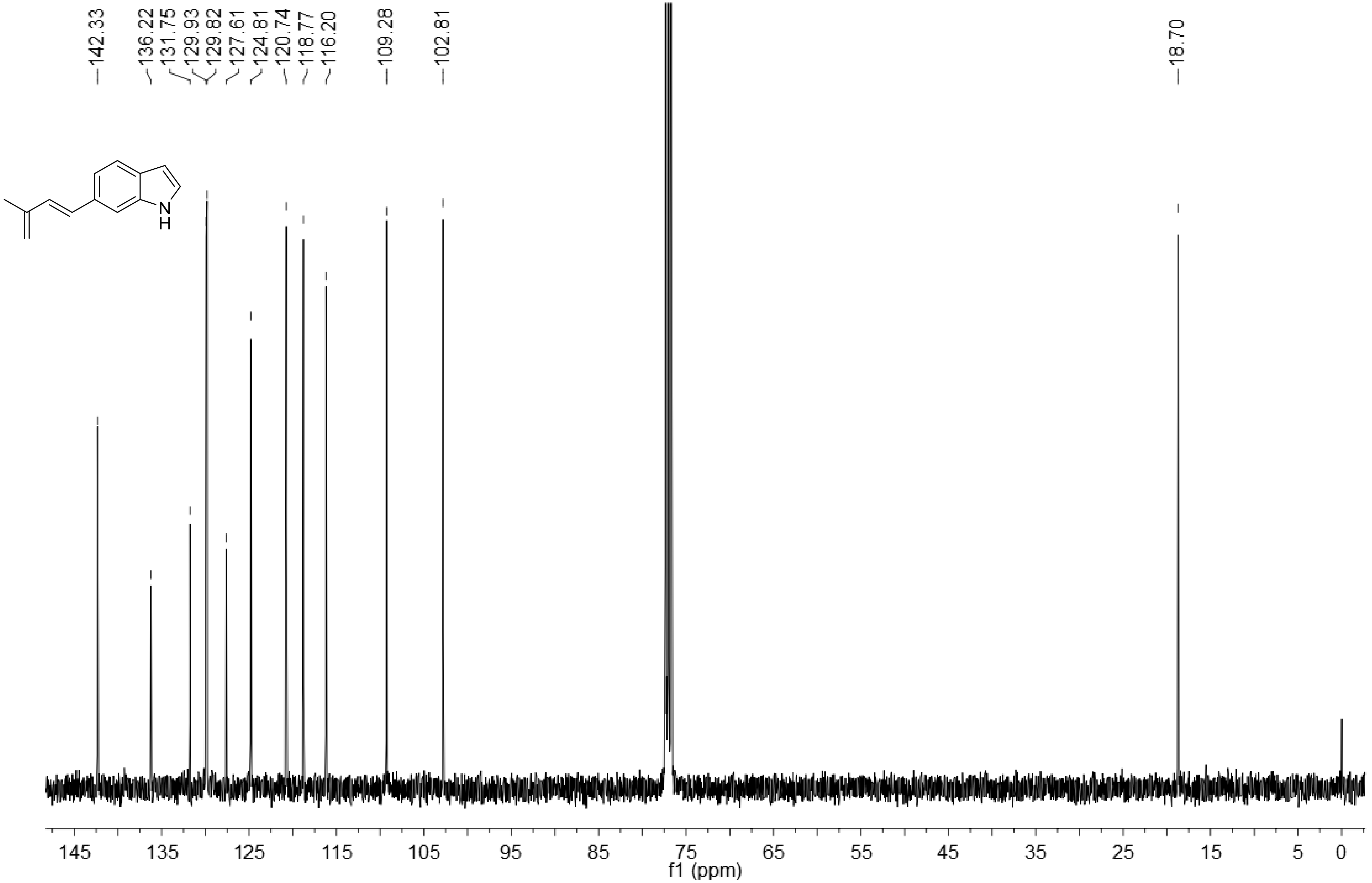

^{1}H NMR ($CDCl_3$, 400 MHz)

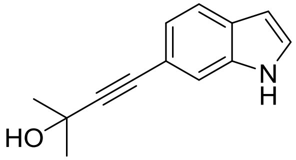

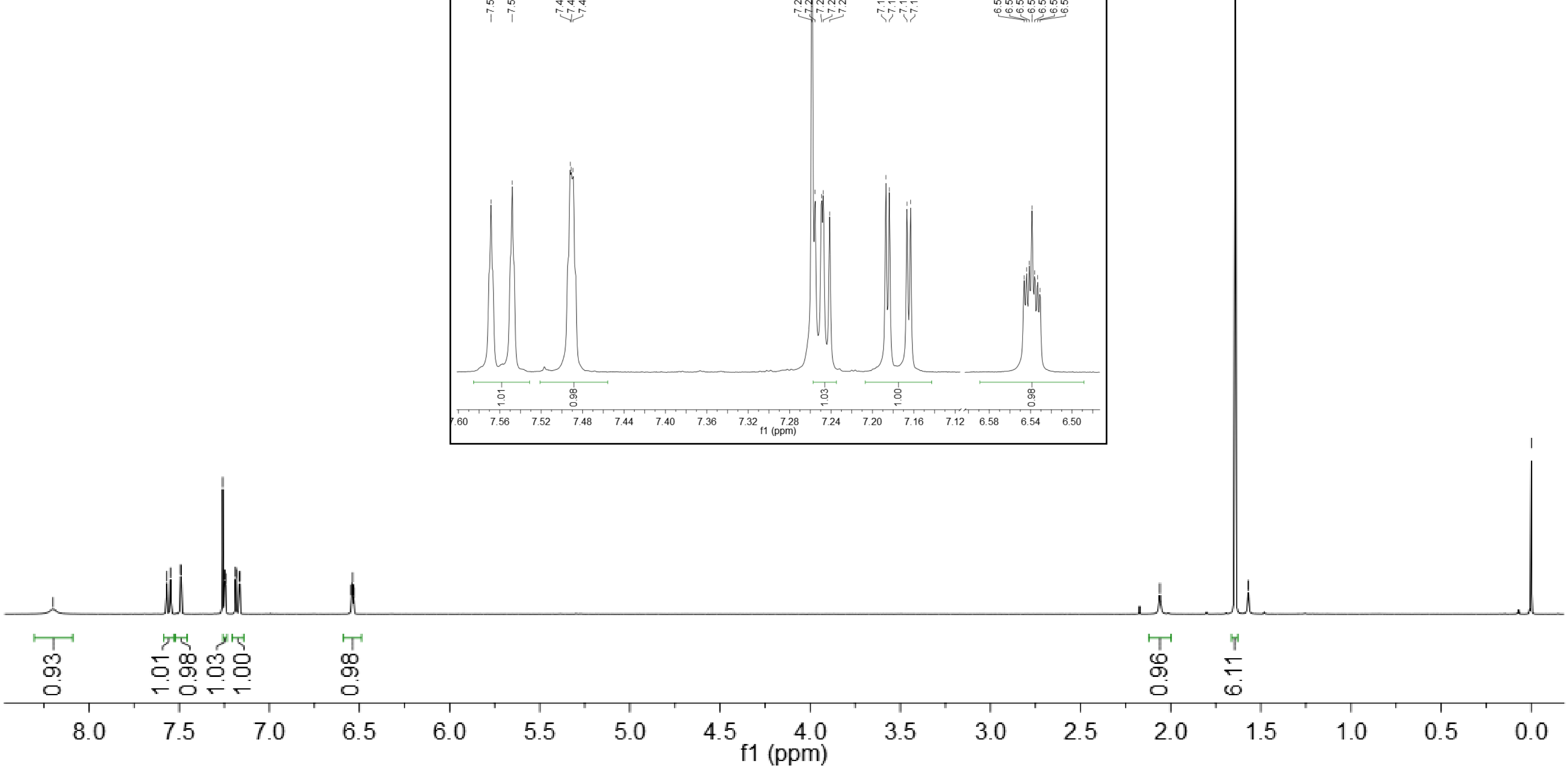

^{13}C NMR ($CDCl_3$, 100 MHz)

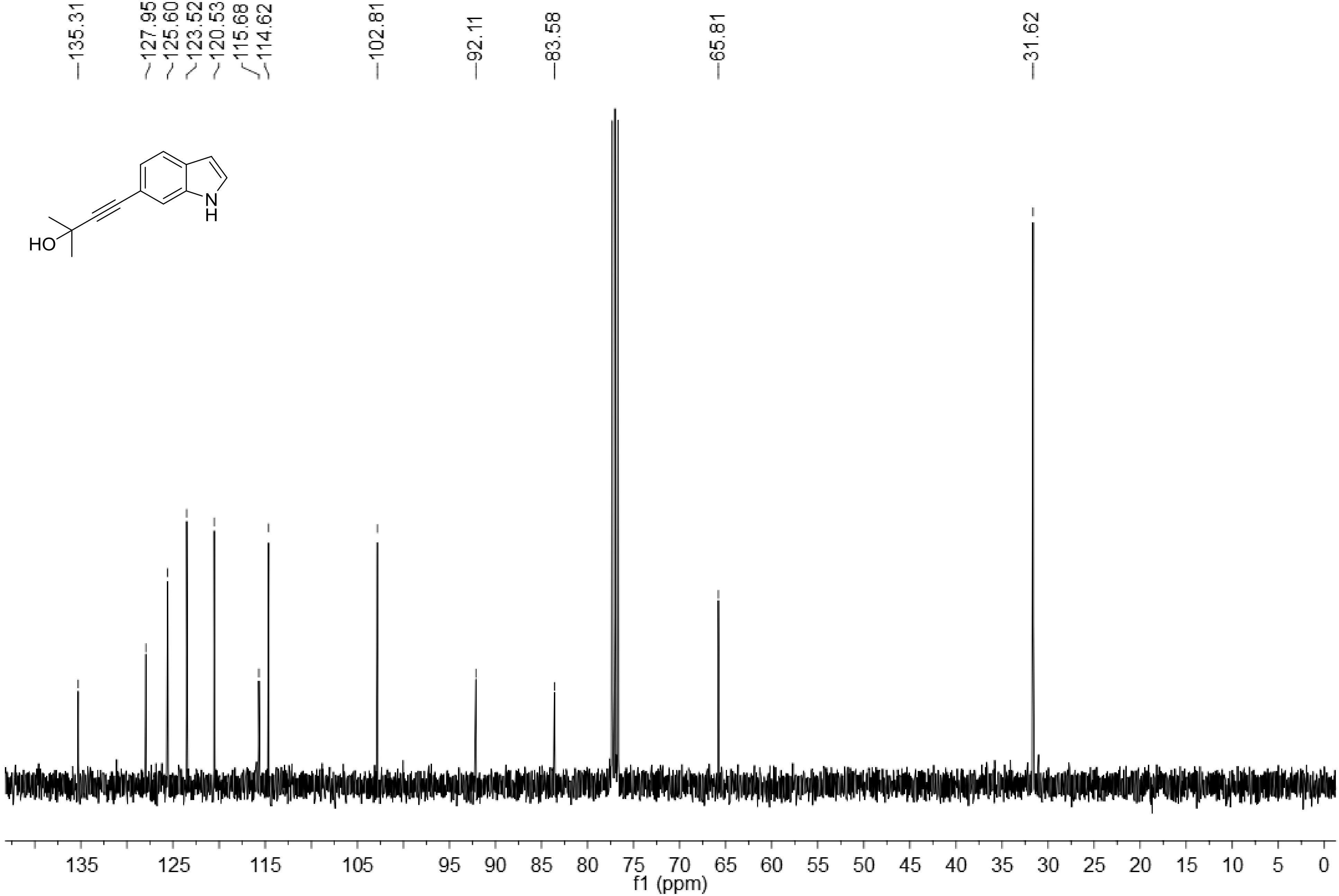

^{1}H NMR ($CDCl_3$, 400 MHz)

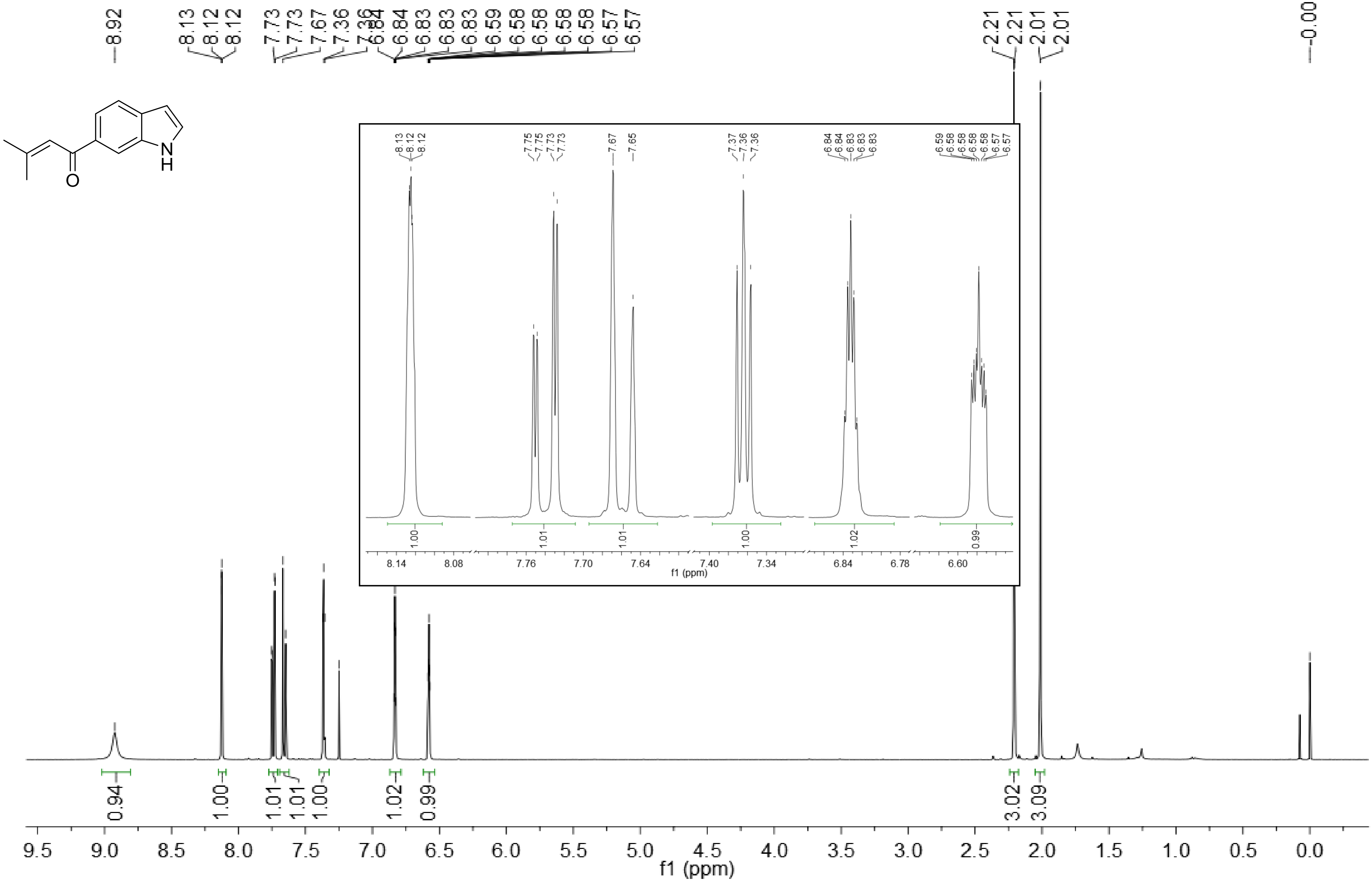

^{13}C NMR ($CDCl_3$, 100 MHz)

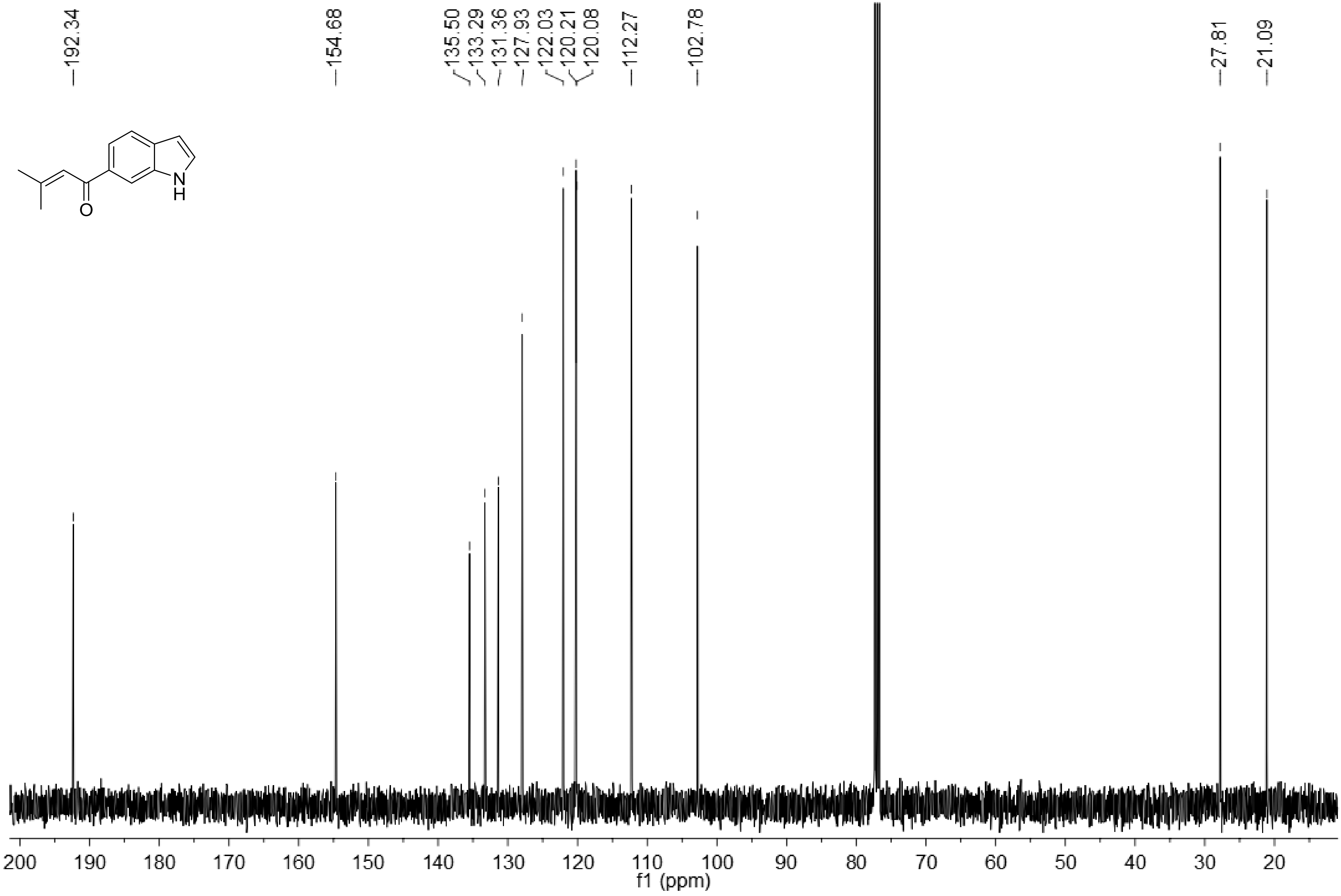

^{1}H NMR (acetone-d_6, 600 MHz)

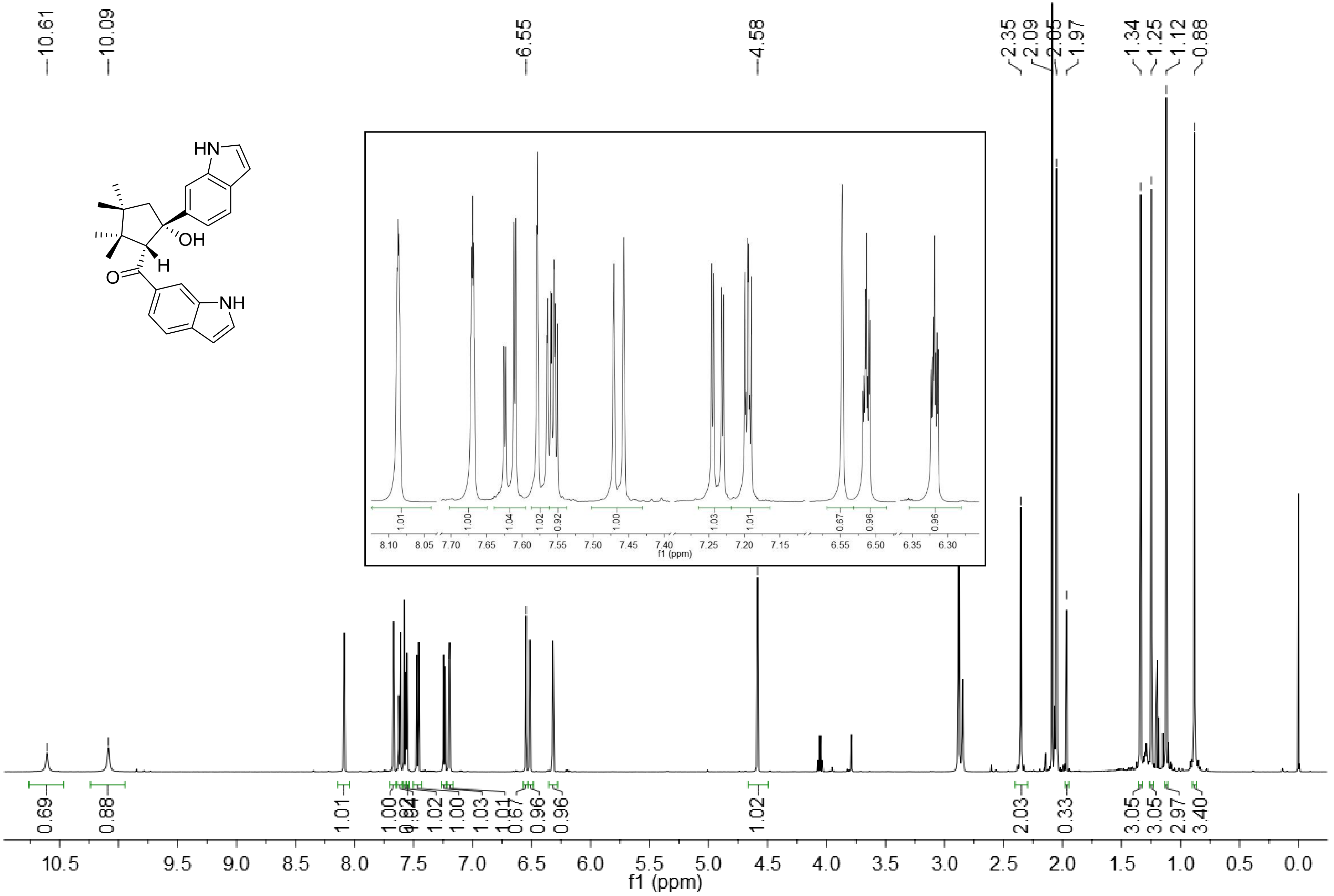

^{13}C NMR (acetone-d_6, 150 MHz)

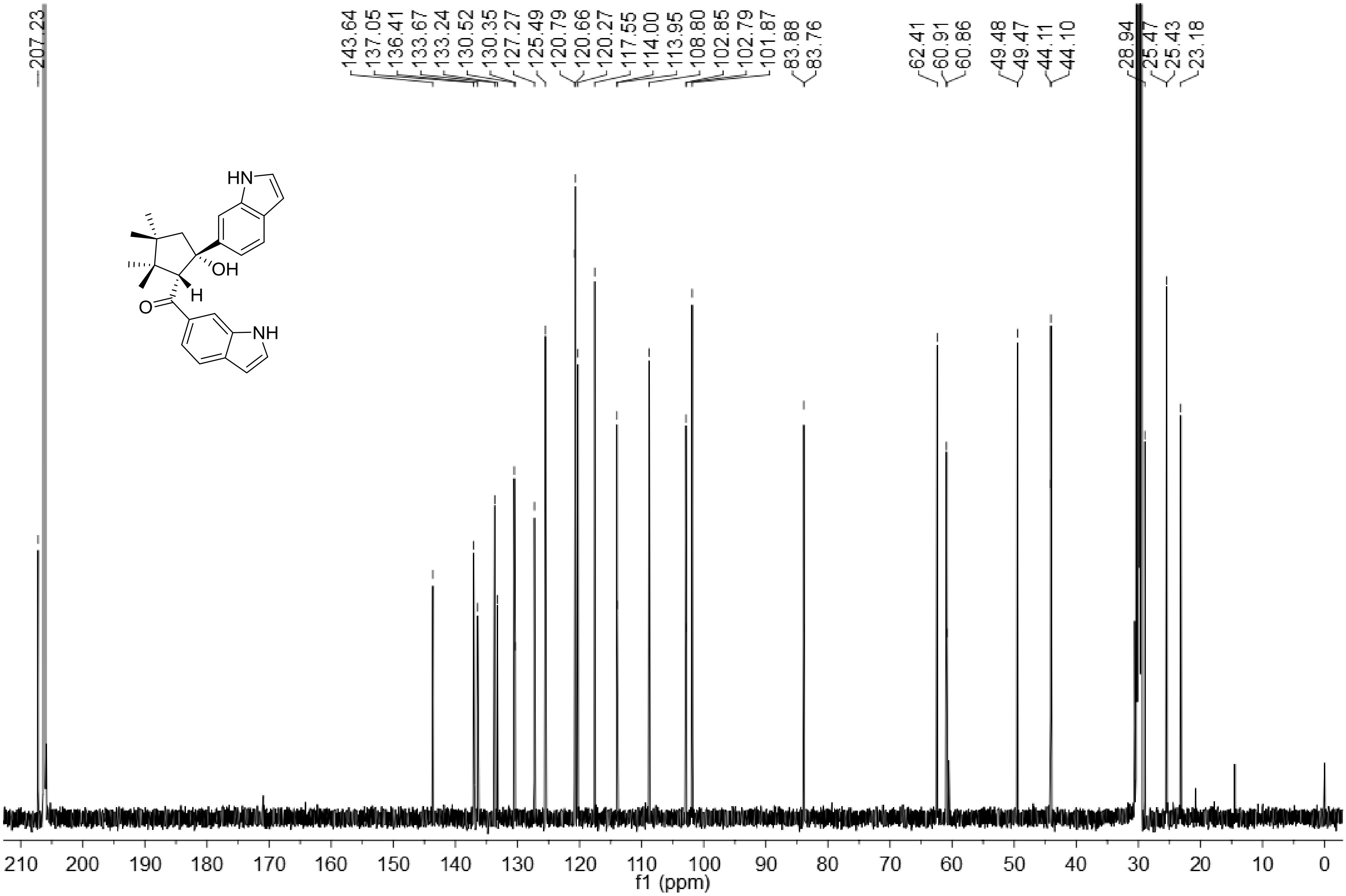

^1H NMR ($CDCl_3$, 400 MHz)

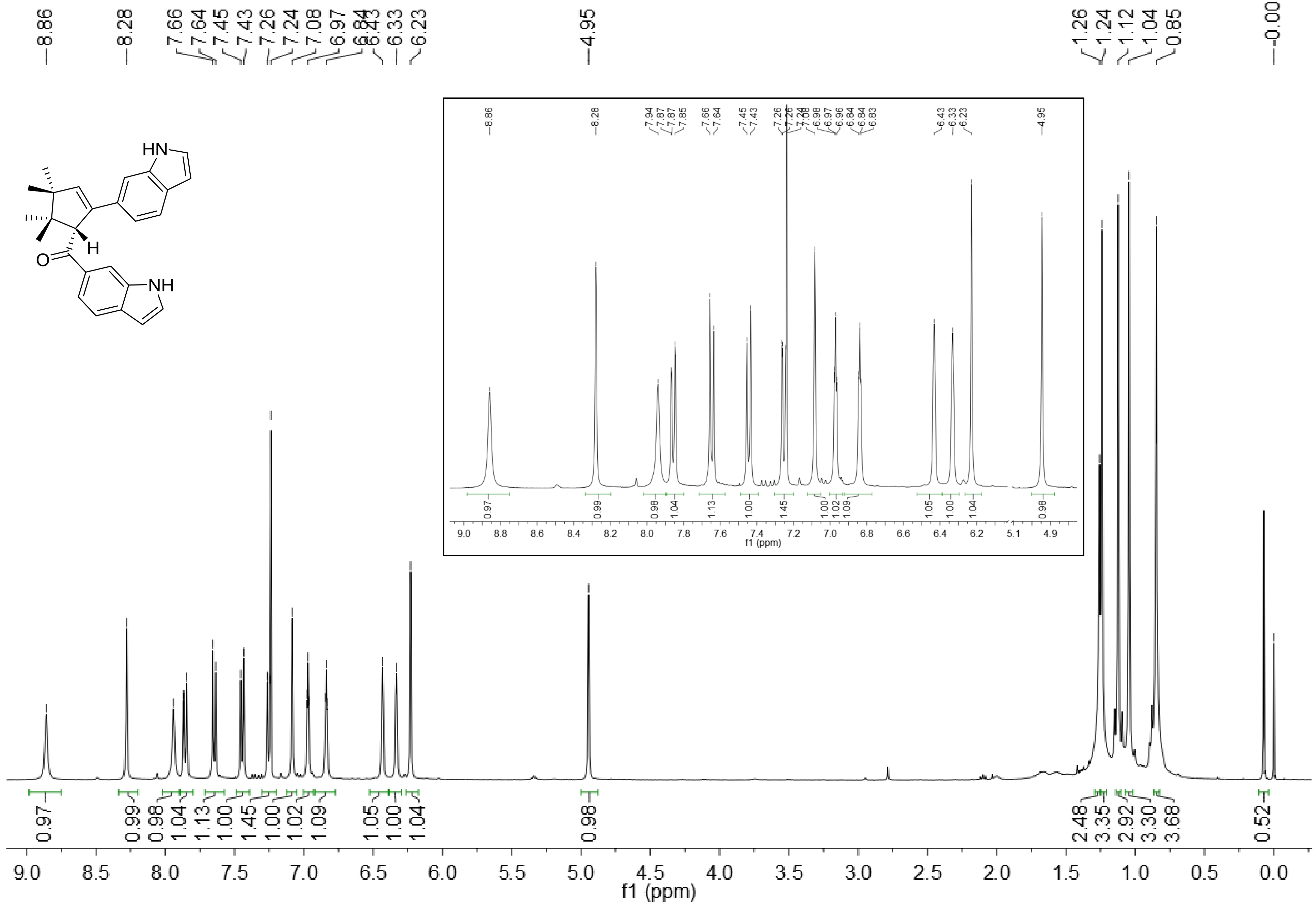

^{13}C NMR ($CDCl_3$, 100 MHz)

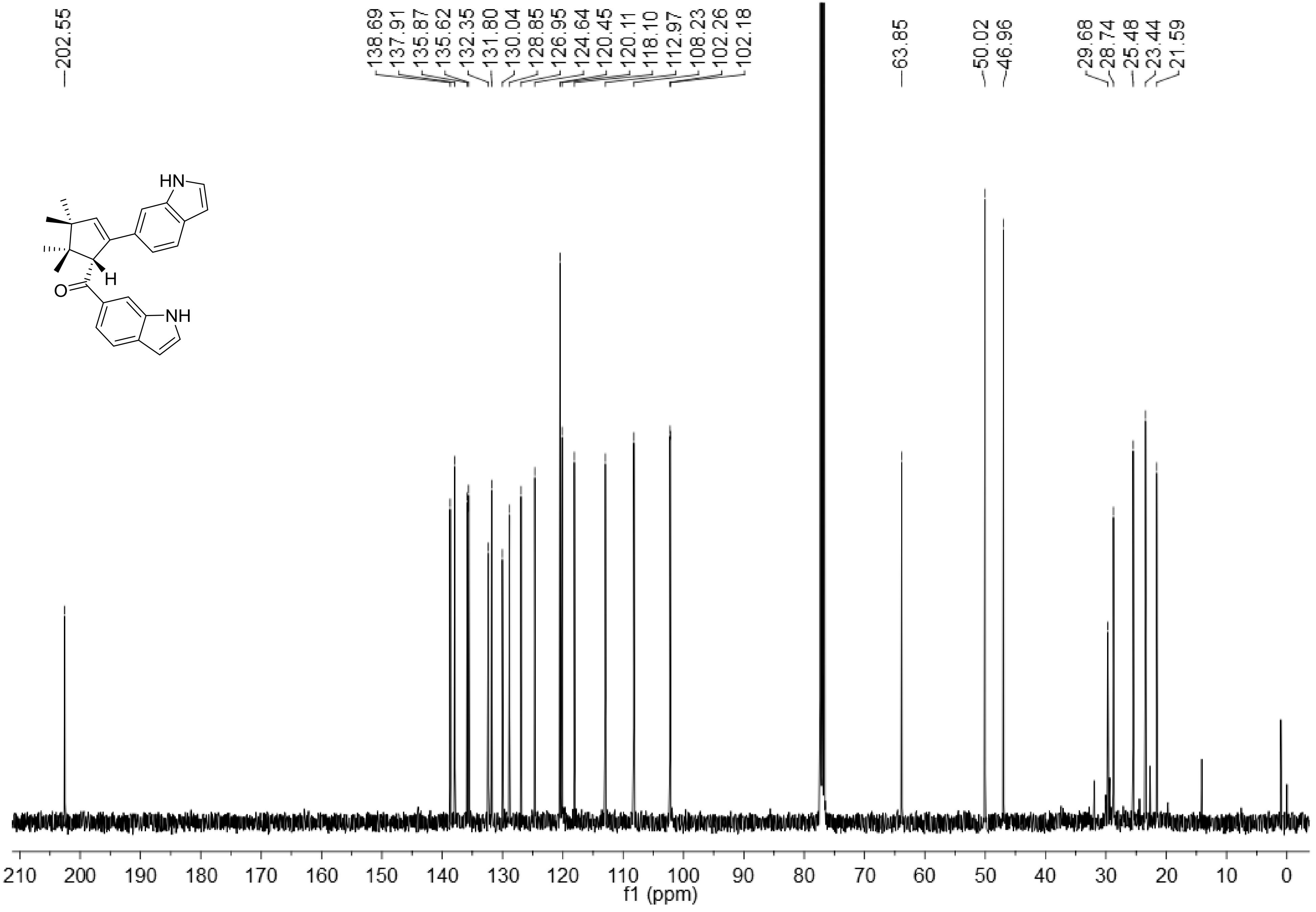

^{1}H NMR ($CDCl_3$, 400 MHz)

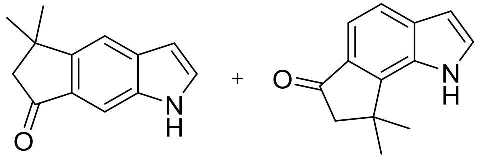

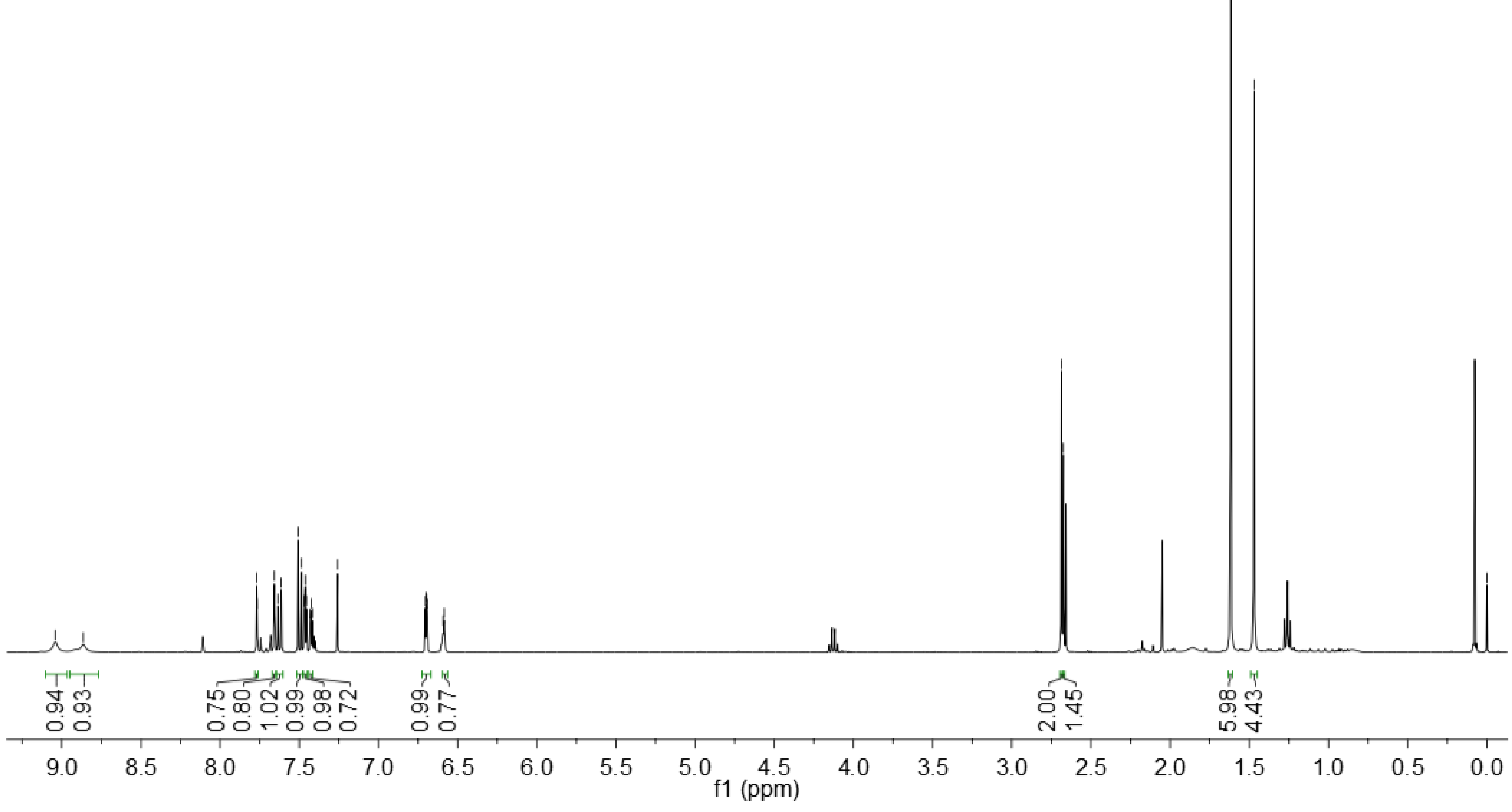

^{13}C NMR ($CDCl_3$, 100 MHz)

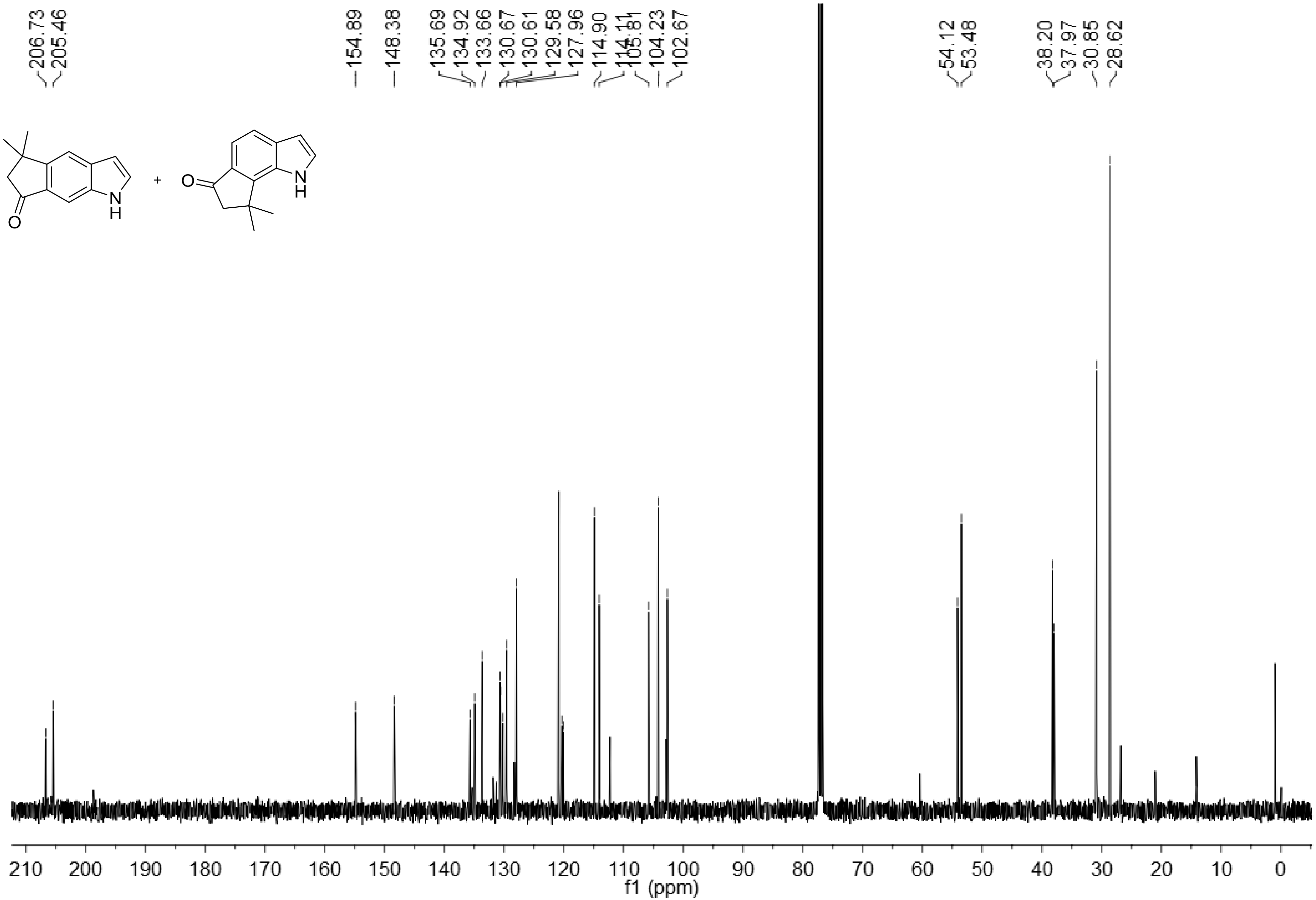

^1H NMR ($CDCl_3$, 400 MHz)

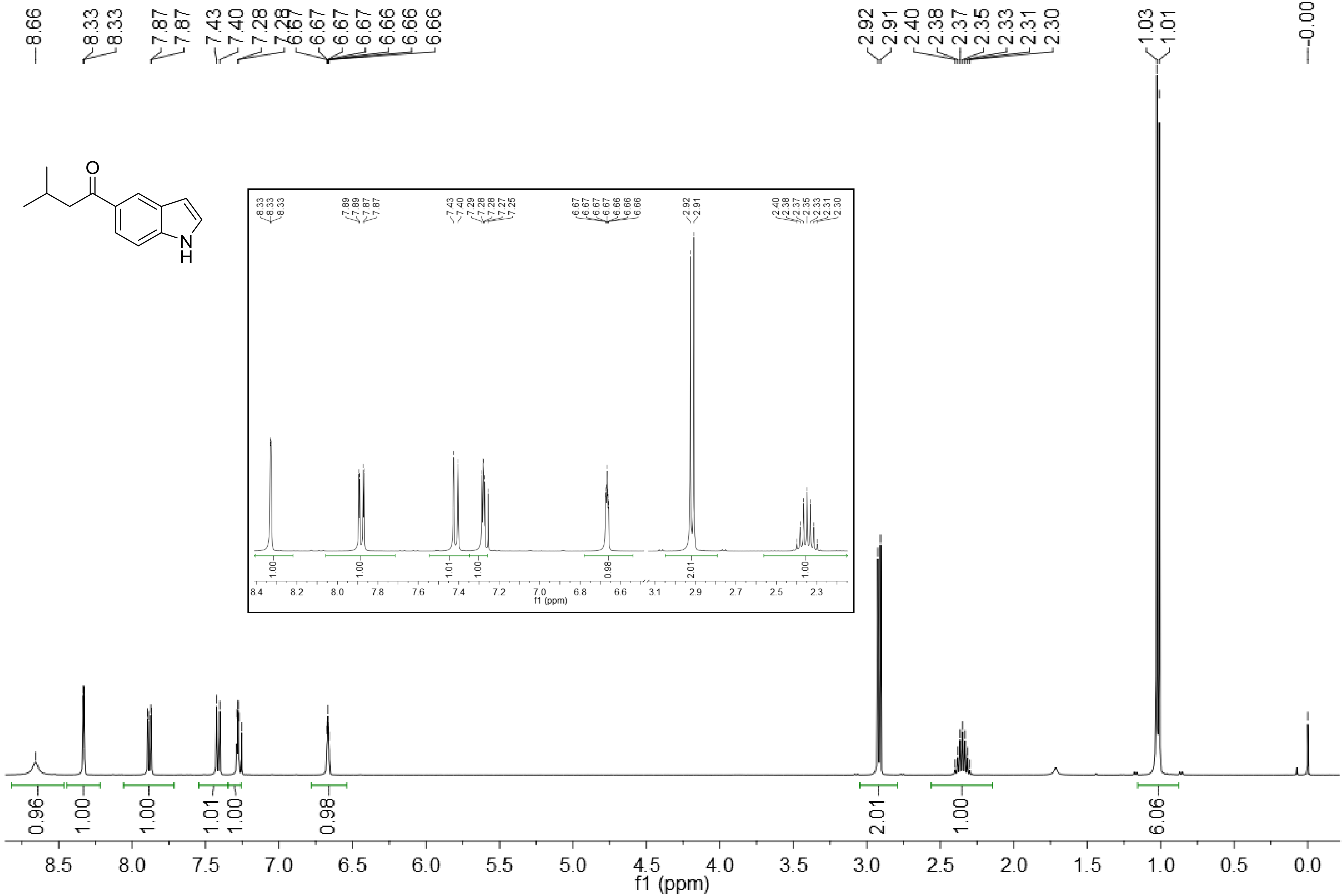

^{13}C NMR ($CDCl_3$, 100 MHz)

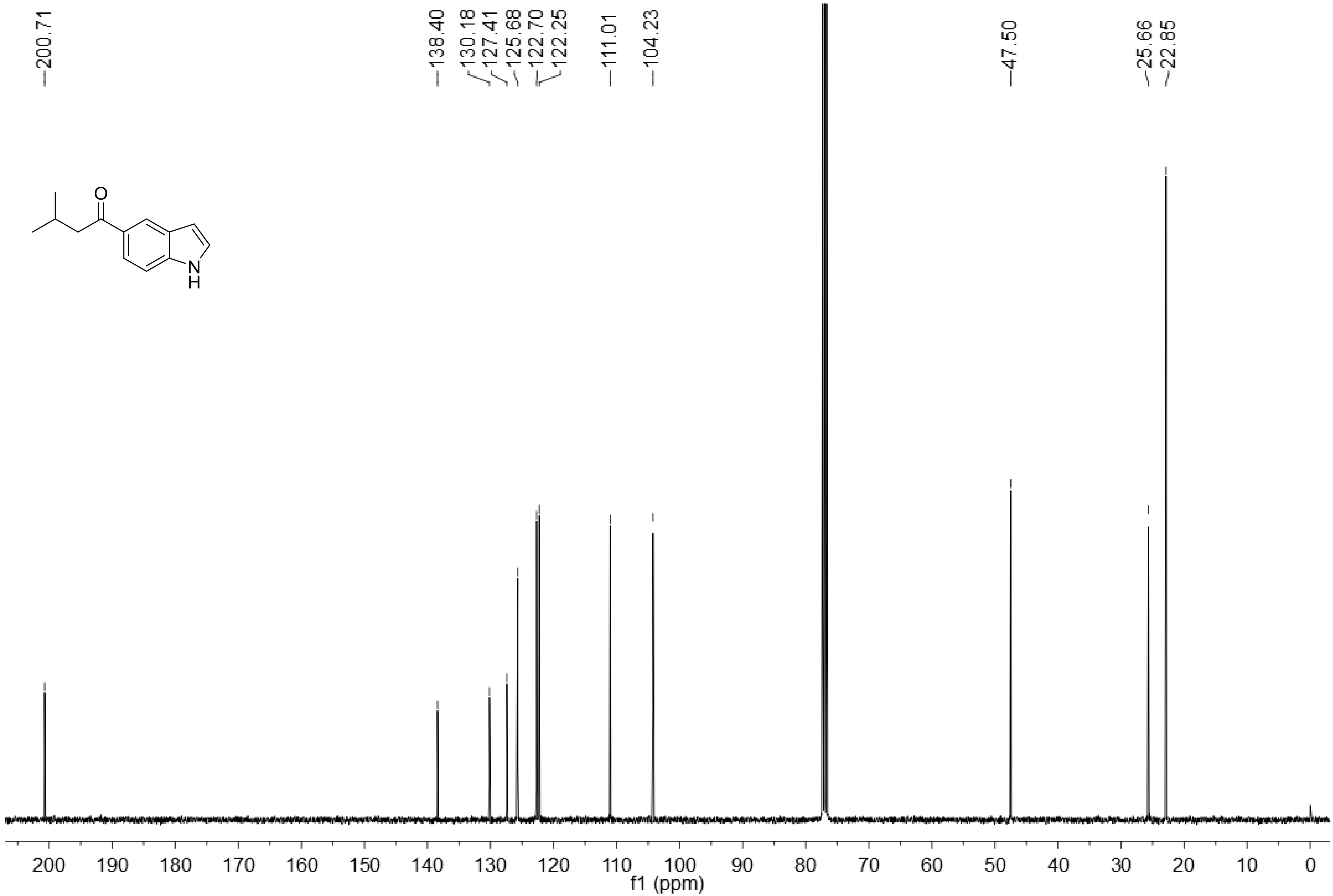

^{1}H NMR ($CDCl_3$, 400 MHz)

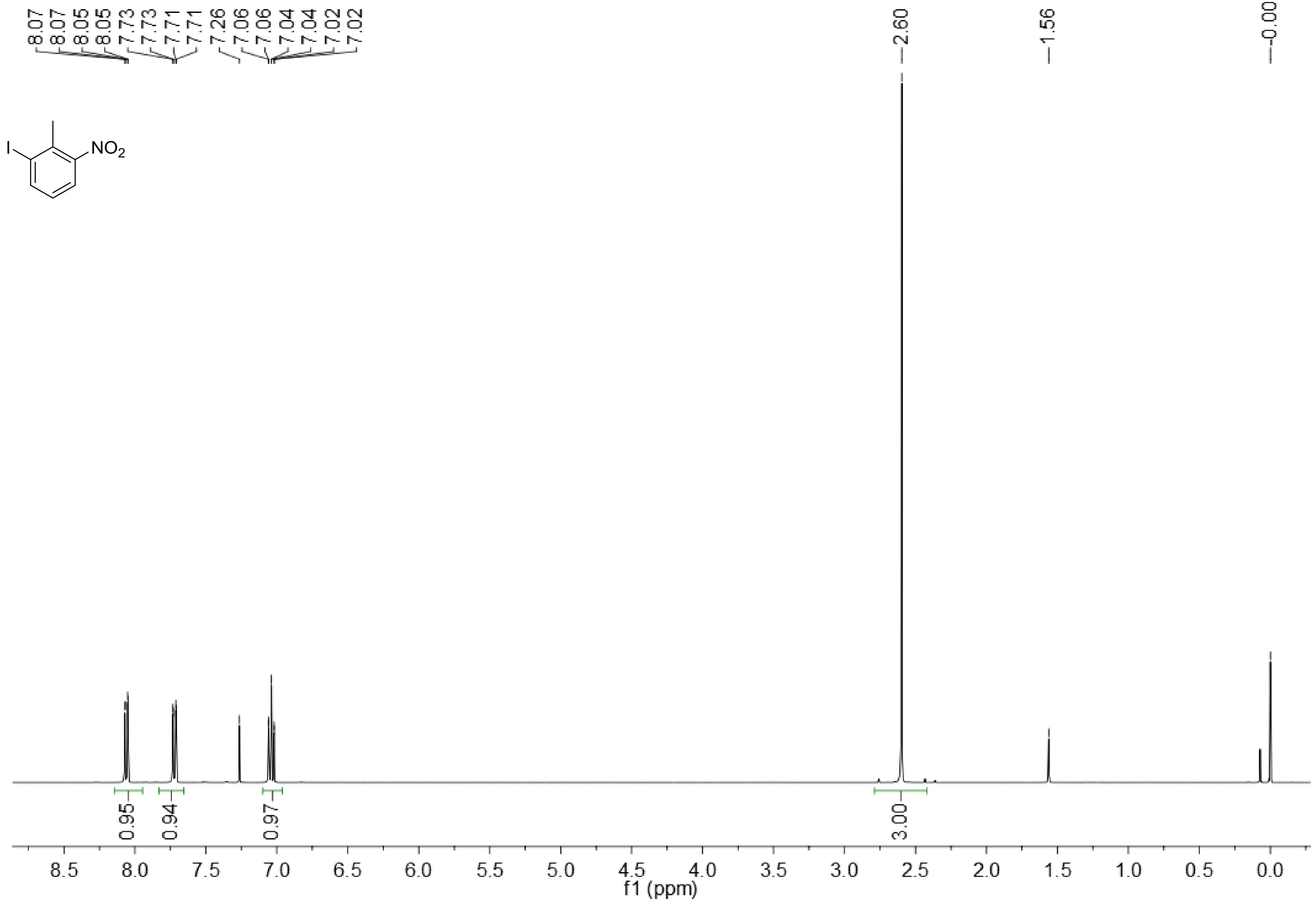

^{13}C NMR ($CDCl_3$, 100 MHz)

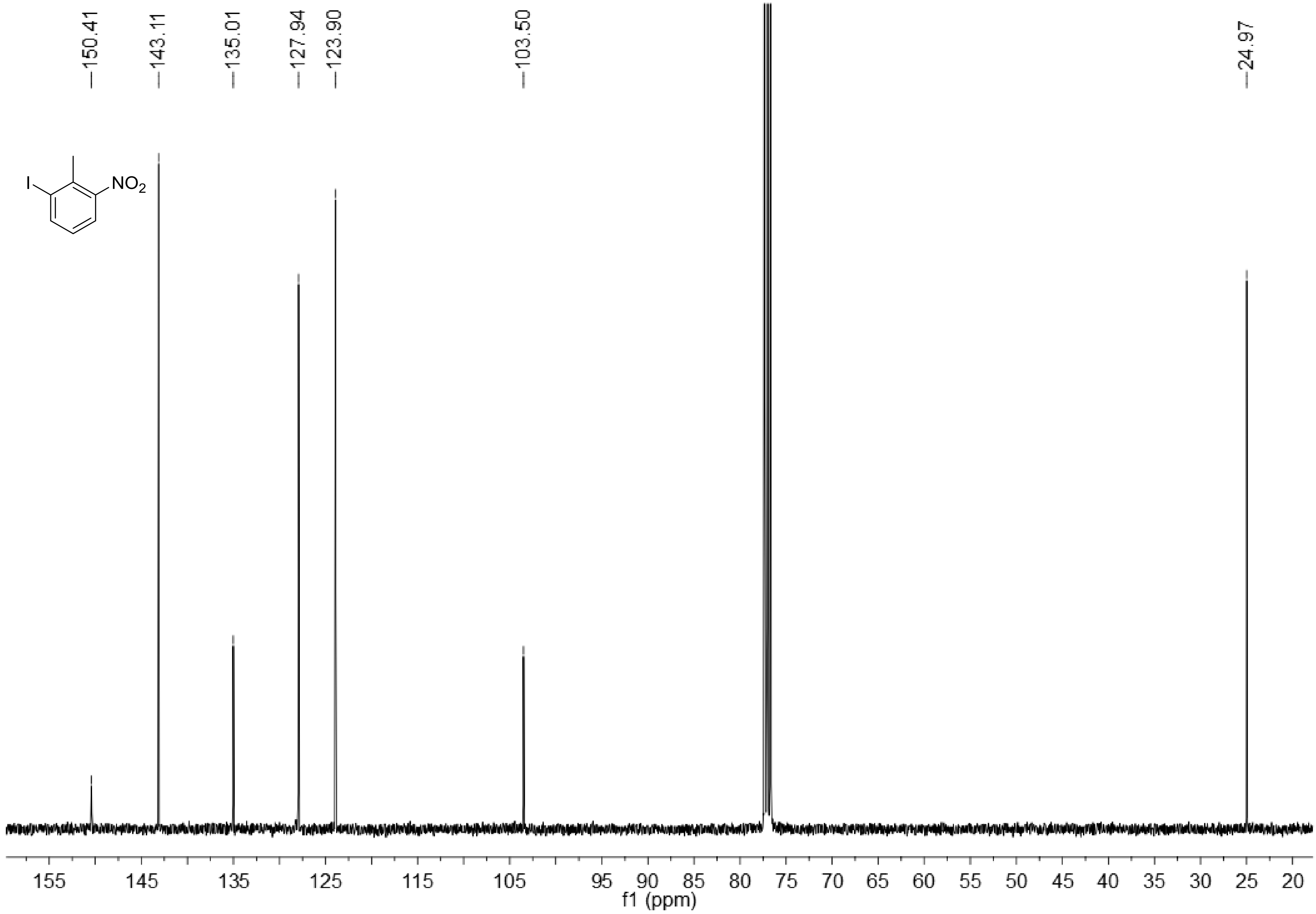

^{1}H NMR ($CDCl_3$, 400 MHz)

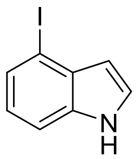

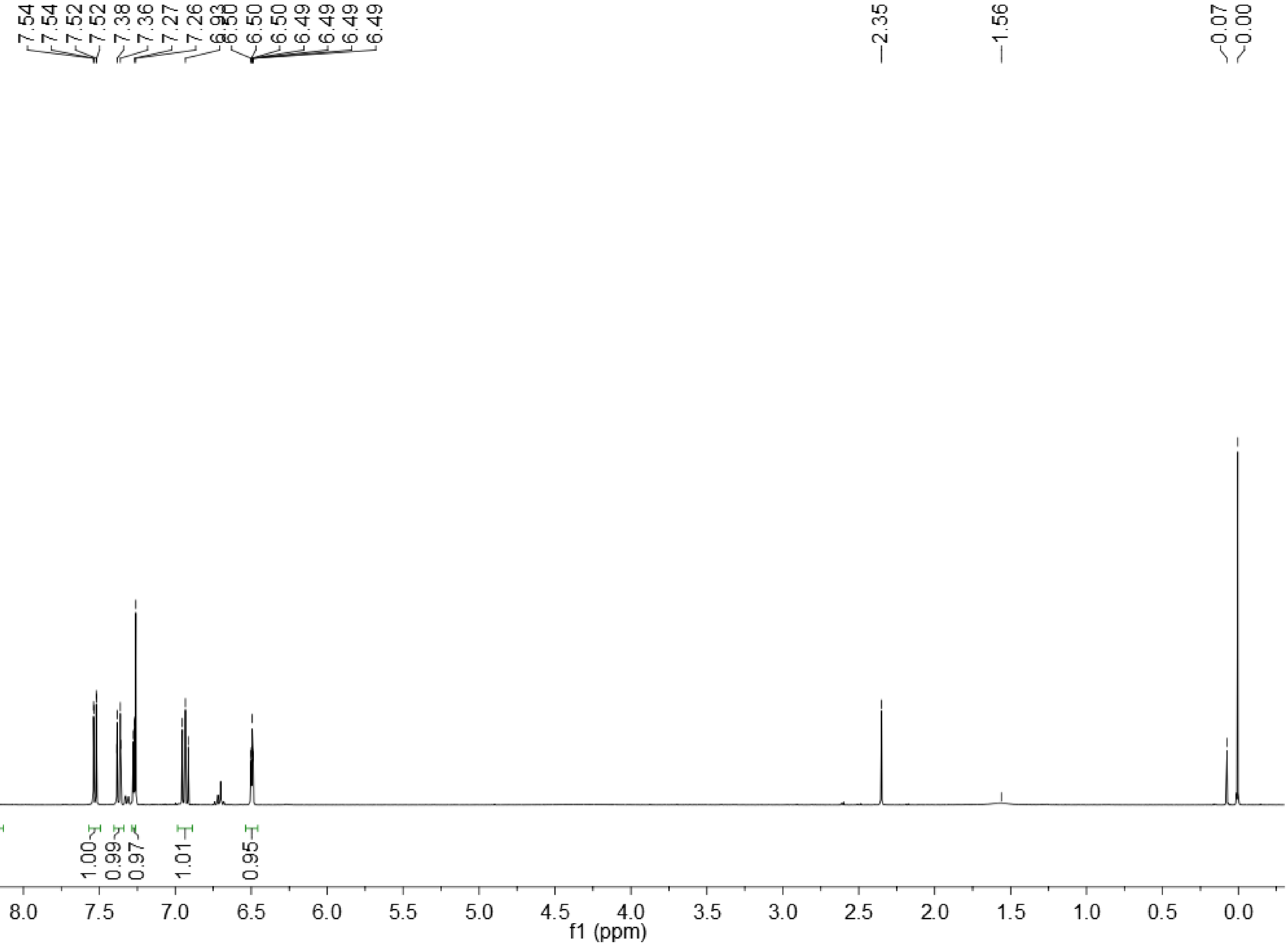

^{13}C NMR ($CDCl_3$, 100 MHz)

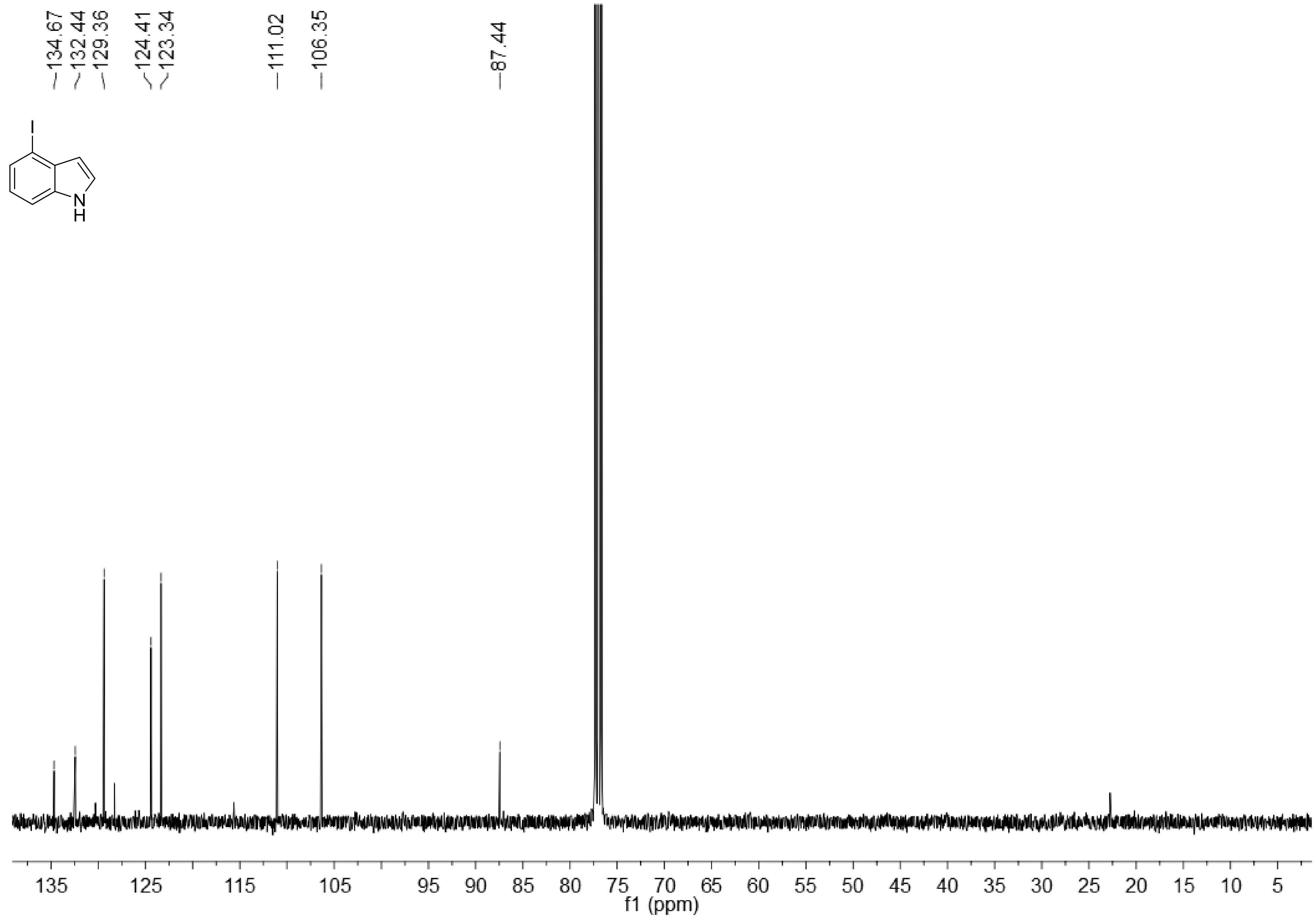

^{1}H NMR ($CDCl_3$, 400 MHz)

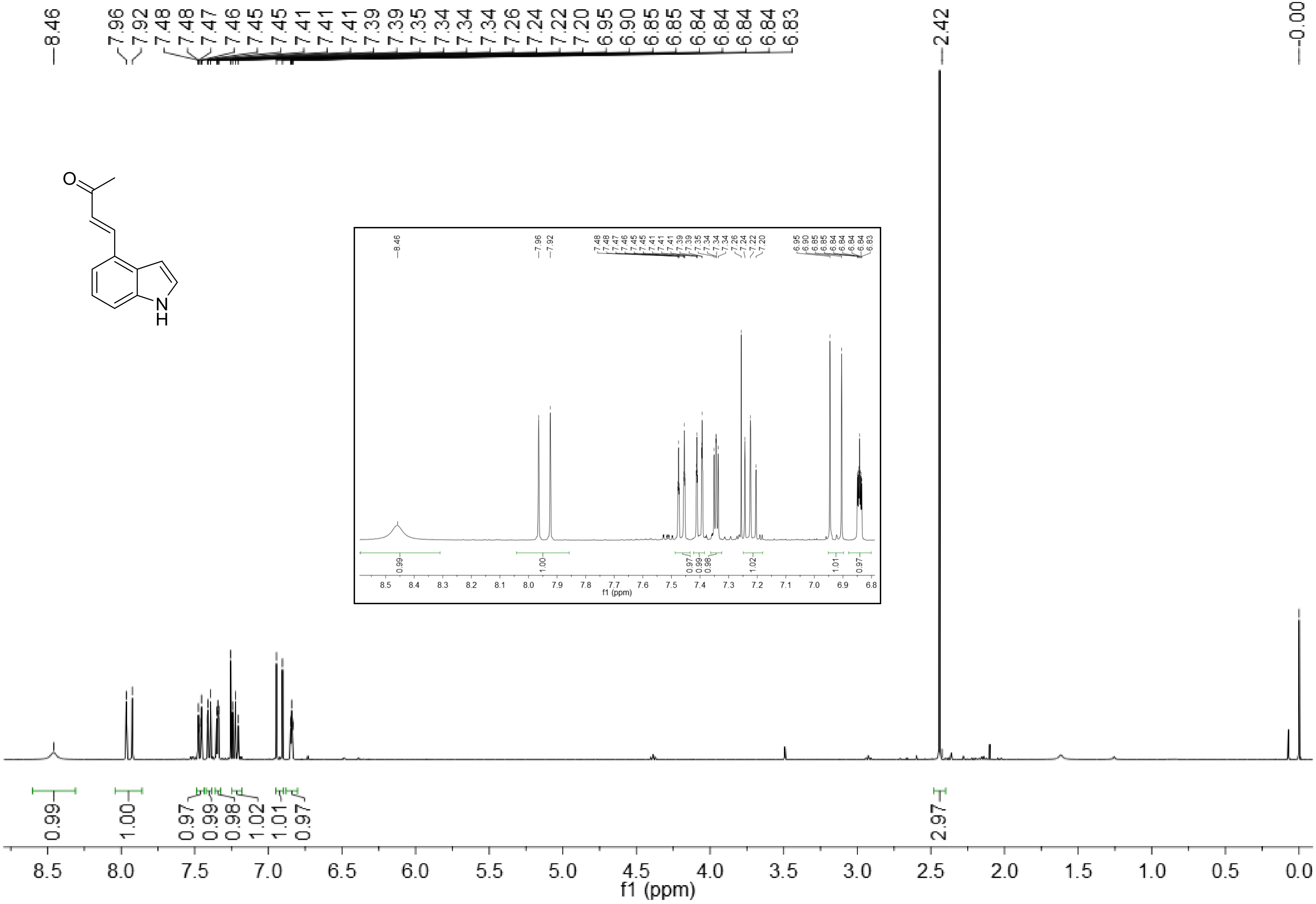

^{13}C NMR ($CDCl_3$, 100 MHz)

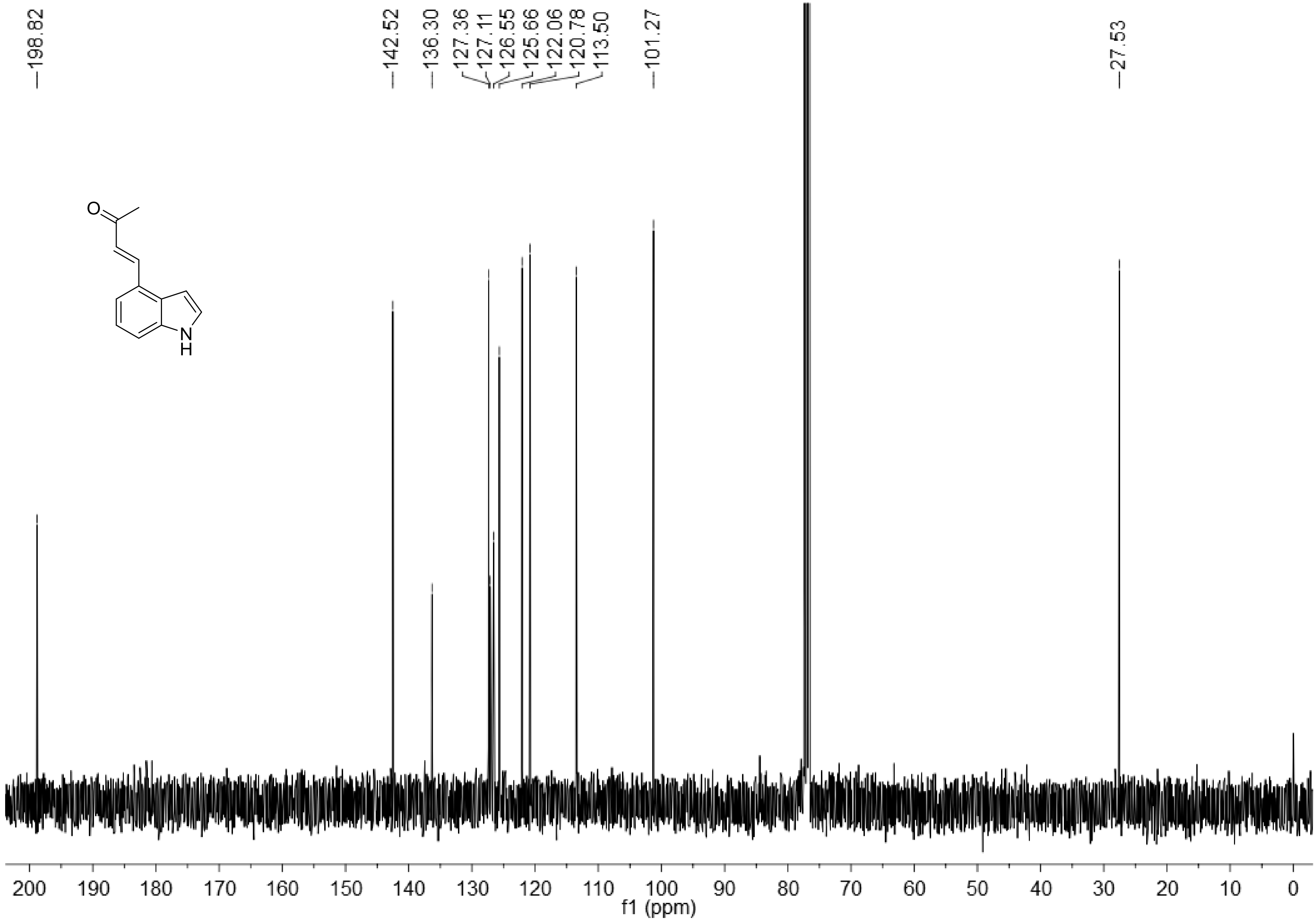

^{1}H NMR (acetone-d_6, 600 MHz)

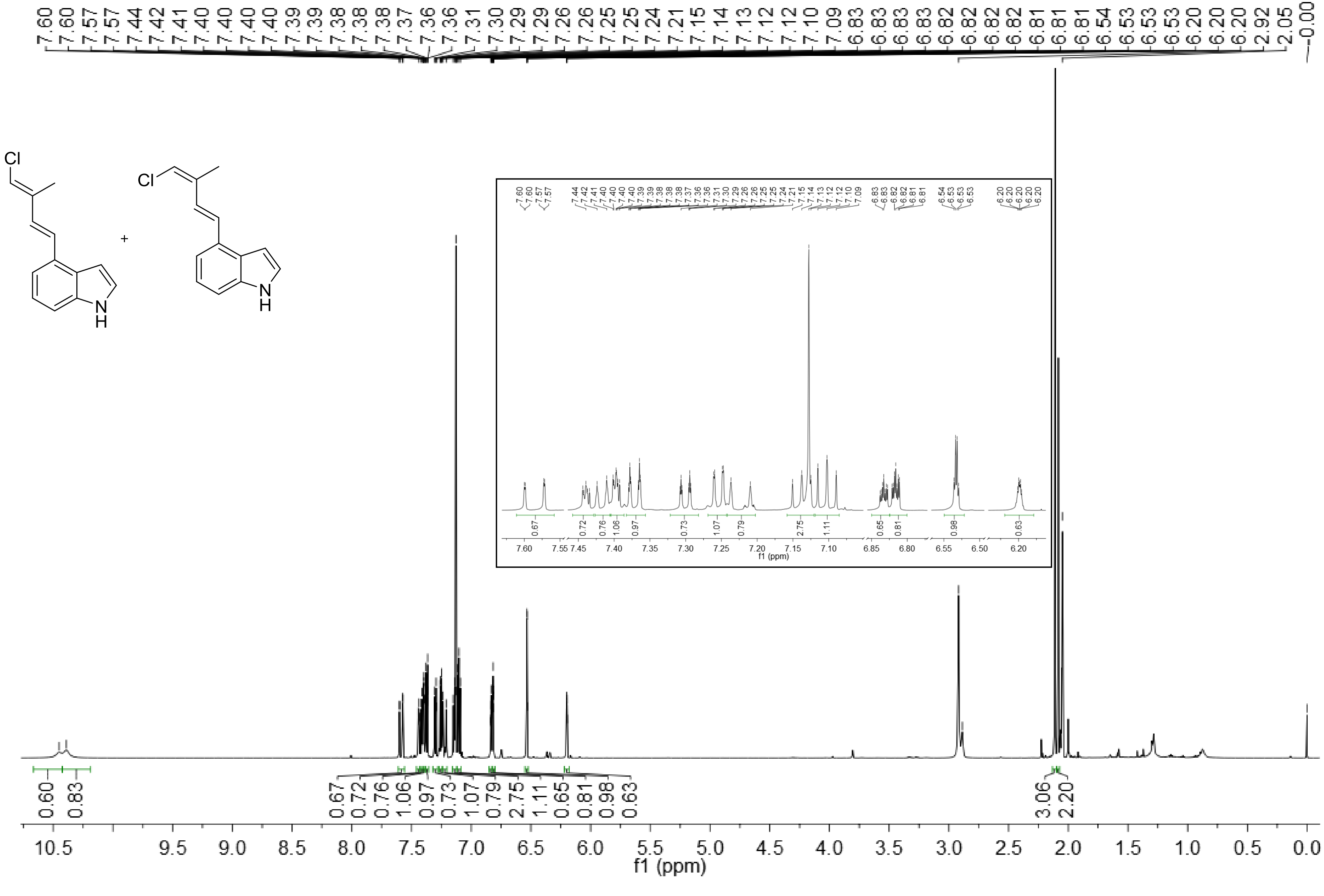

^{13}C NMR (acetone-d_6, 150 MHz)

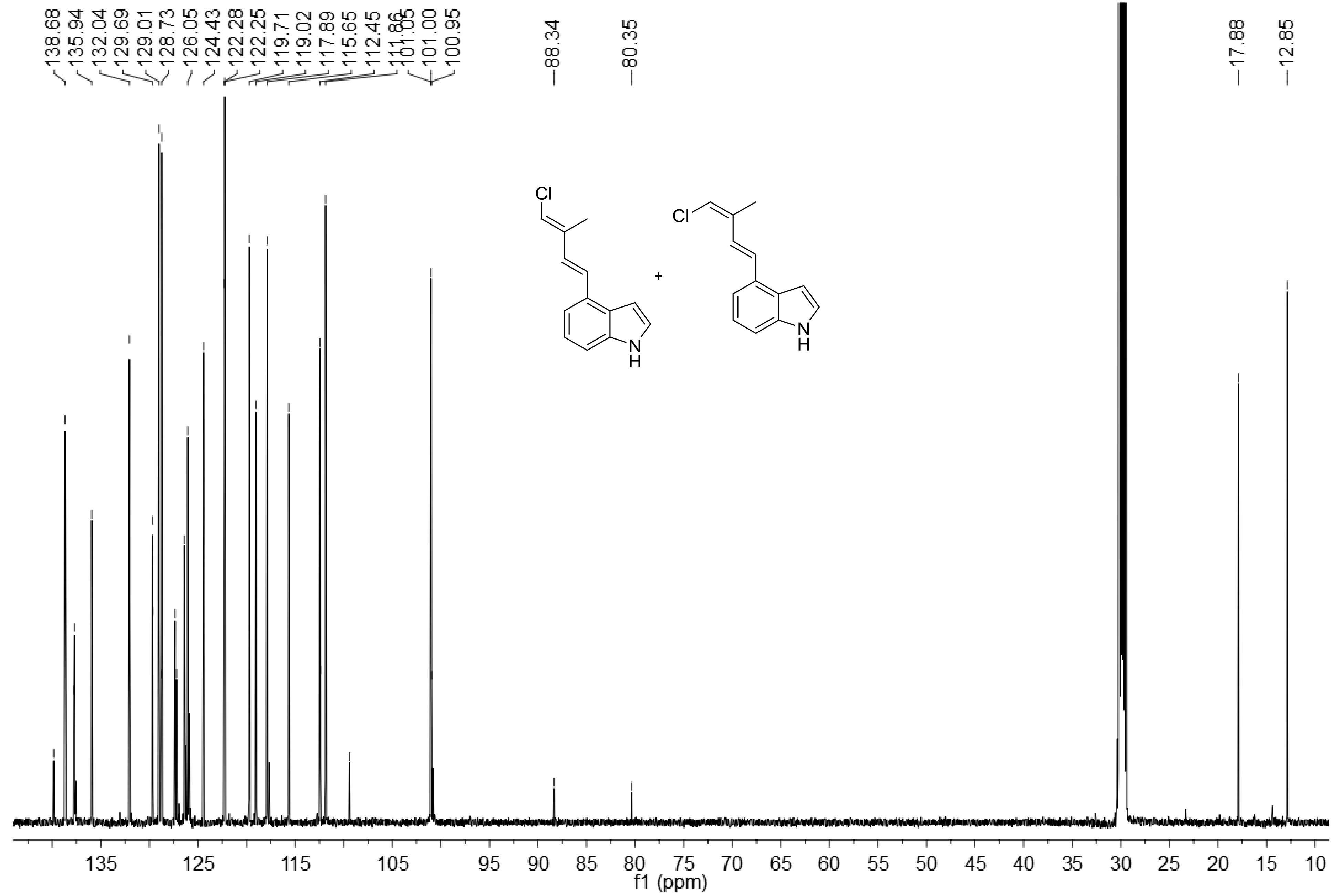

HPLC chromatogram of the separation of *E*- and *Z*-indiacen B

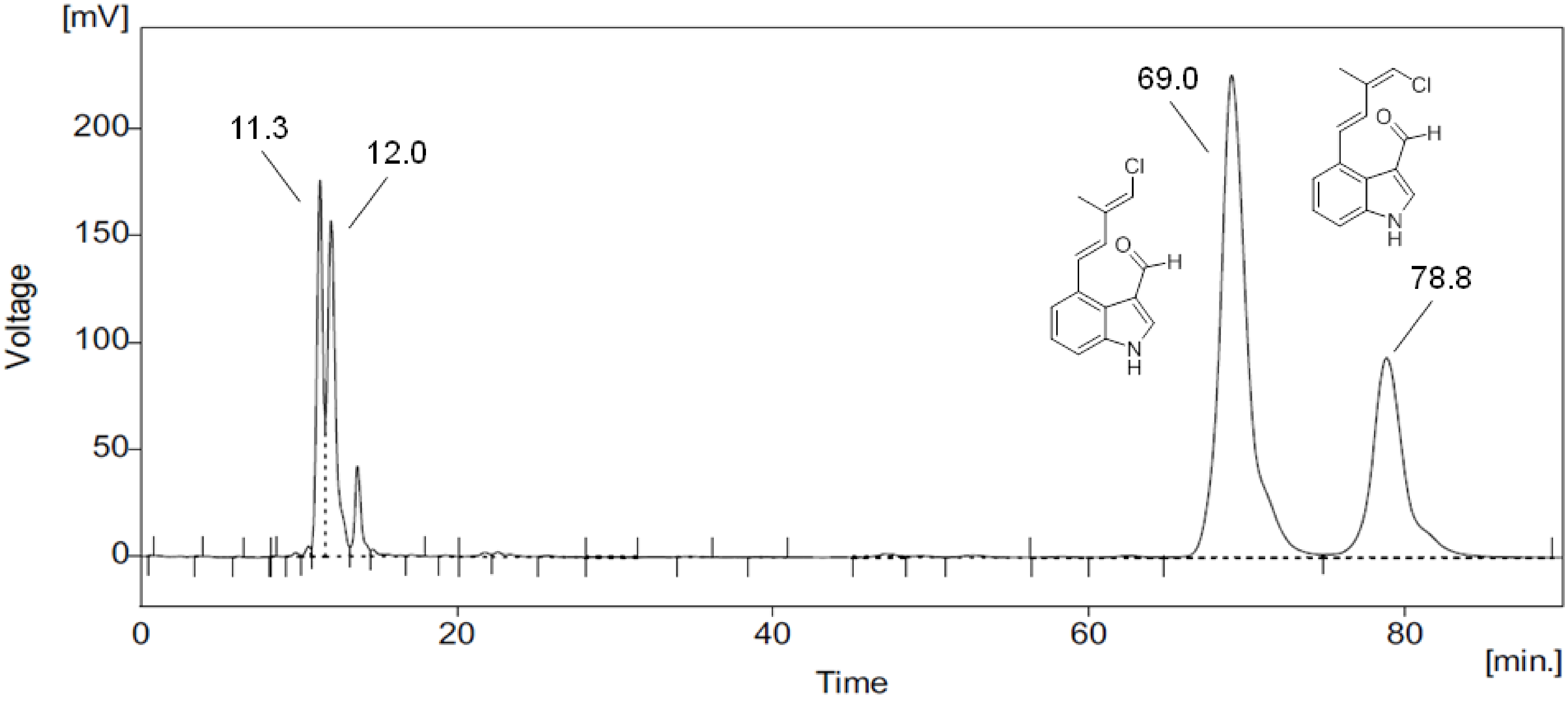

^{1}H NMR (acetone-d_6, 600 MHz)

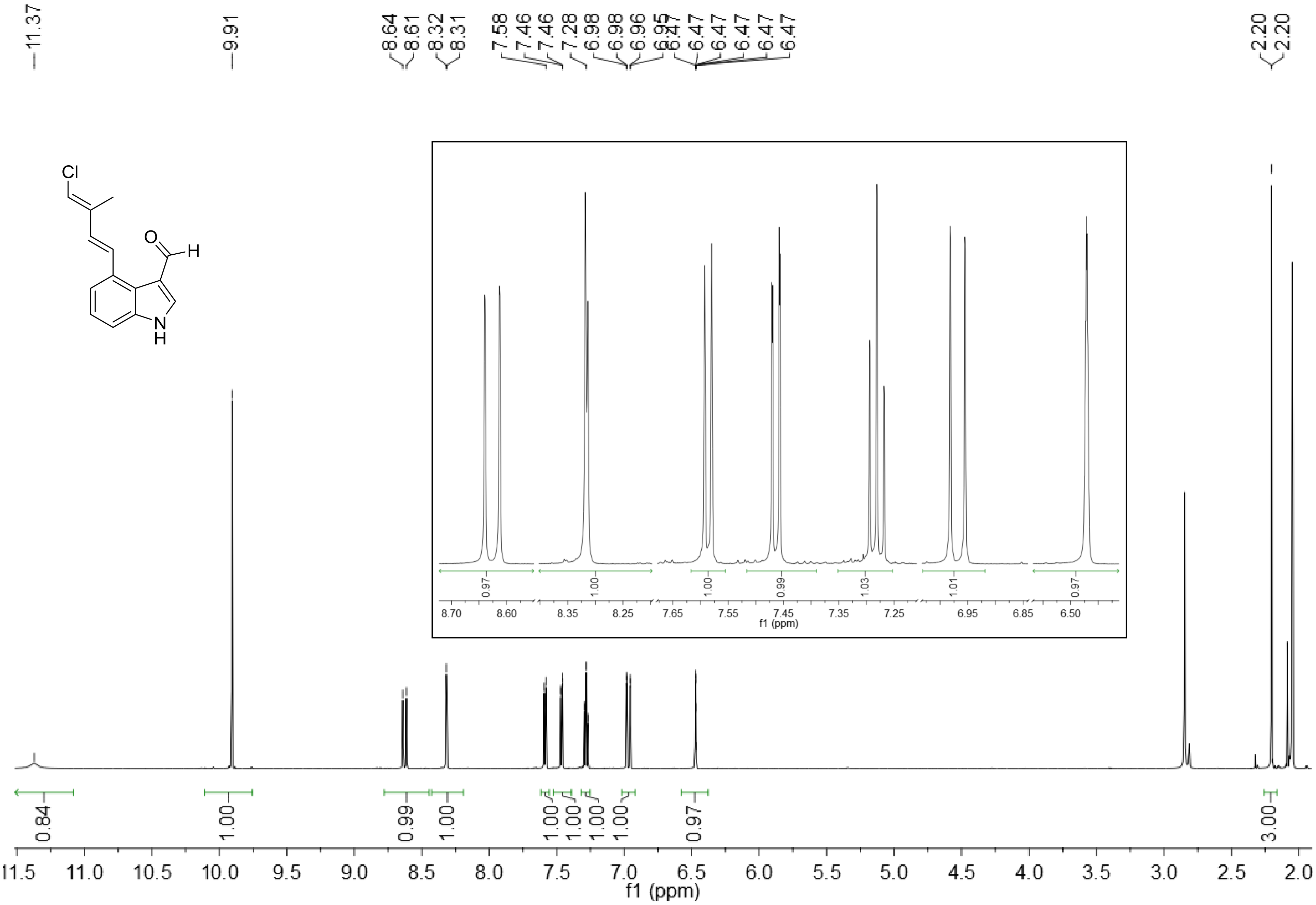

^{13}C NMR (acetone-d_6, 150 MHz)

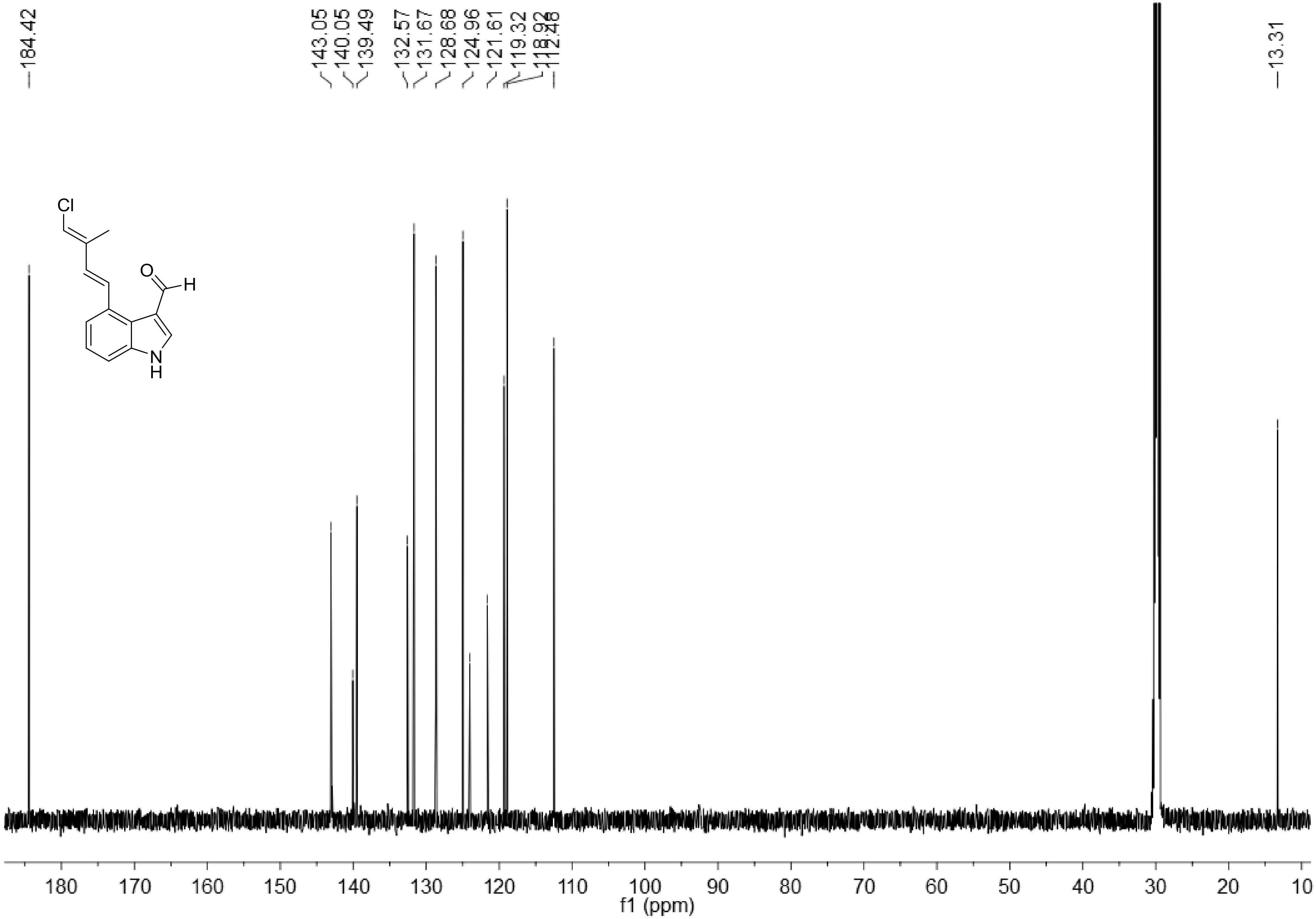

^{1}H NMR (acetone-d_6, 600 MHz)

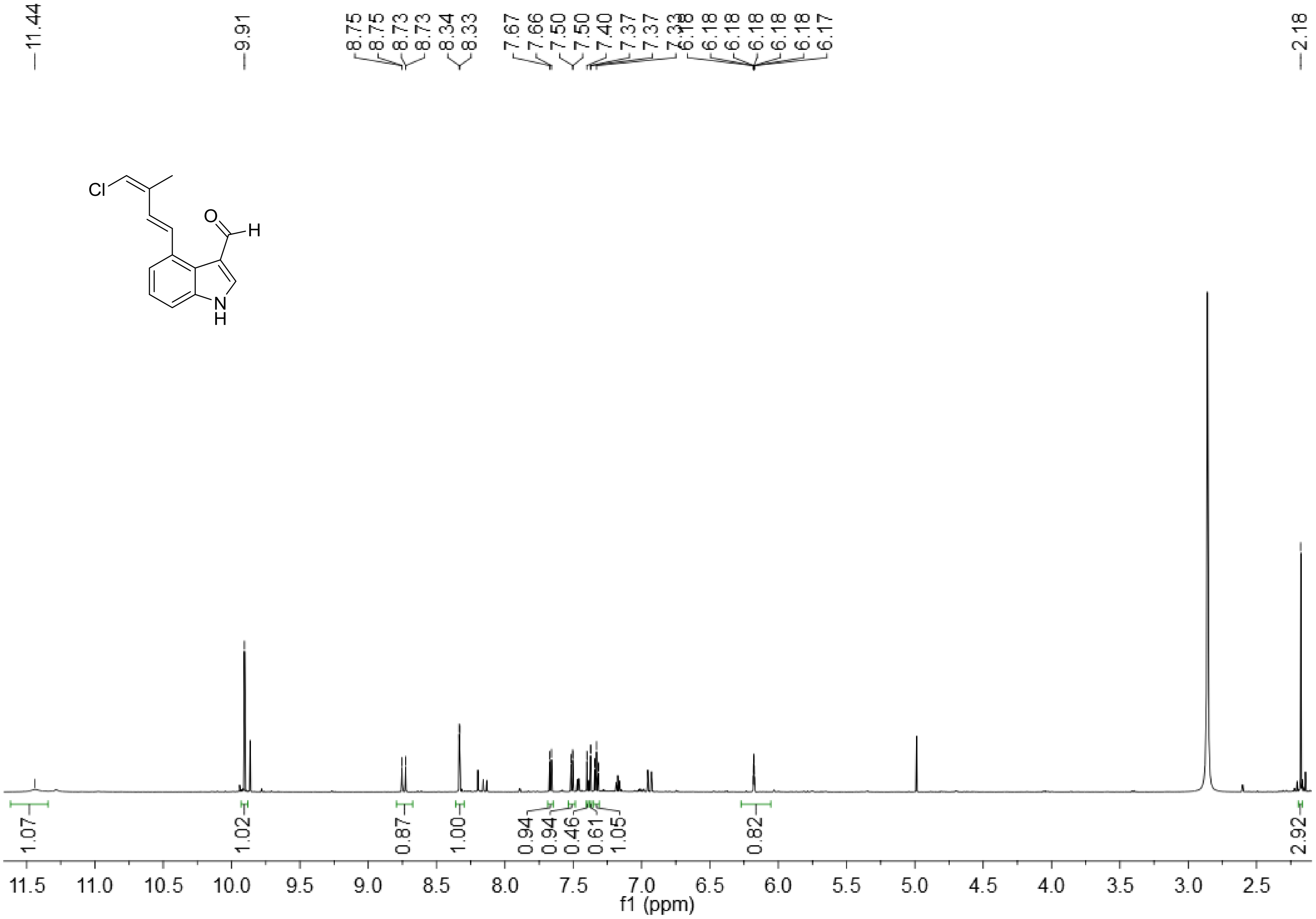

^{13}C NMR (acetone-d_6, 150 MHz)

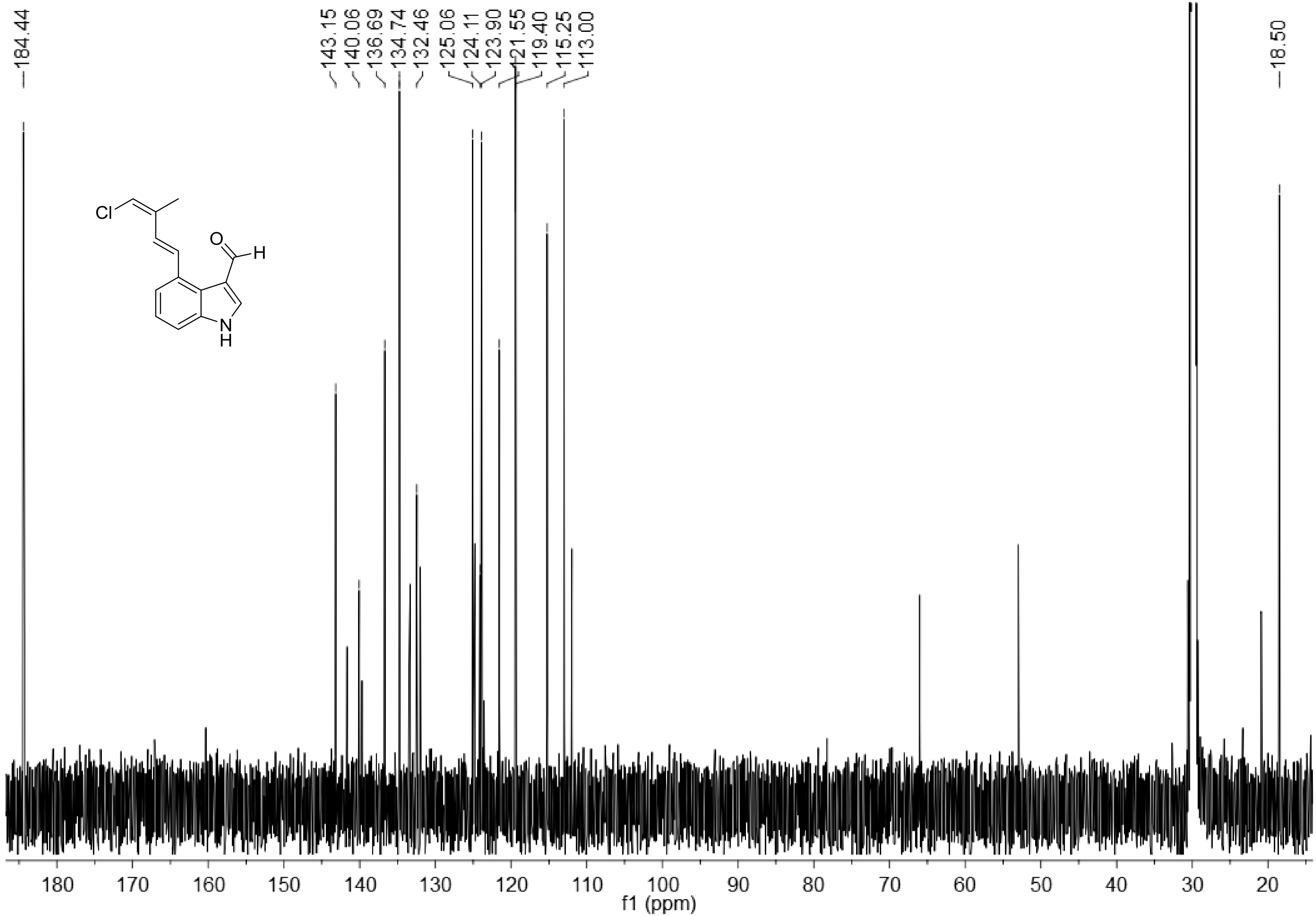

Supplement: File 1 — Experimental procedures, bioactivity tables, X-ray figures and tables, HPLC chromatograms and NMR spectra for all compounds, NOESY analyses. [file Beilstein_J_Org_Chem-11-1700-s001.pdf]
